# *In vitro* modelling of anterior primitive streak patterning with human pluripotent stem cells identifies the path to notochord progenitors

**DOI:** 10.1242/dev.202983

**Published:** 2024-12-12

**Authors:** Miguel Robles-Garcia, Chloë Thimonier, Konstantina Angoura, Ewa Ozga, Heather MacPherson, Guillaume Blin

**Affiliations:** ^1^ Centre for Regenerative Medicine, Institute for Regeneration and Repair, The University of Edinburgh, Edinburgh, EH16 4UU, UK; ^2^ Institute for Stem Cell Research, School of Biological Sciences, The University of Edinburgh, Edinburgh, EH16 4UU, UK

**Keywords:** Gastrulation, Human, Micropatterns, Notochord, Pluripotent stem cells

## Abstract

Notochord progenitors (NotoPs) represent a scarce yet crucial embryonic cell population, playing important roles in embryo patterning and eventually giving rise to the cells that form and maintain intervertebral discs. The mechanisms regulating NotoPs emergence are unclear. This knowledge gap persists due to the inherent complexity of cell fate patterning during gastrulation, particularly within the anterior primitive streak (APS), where NotoPs first arise alongside neuro-mesoderm and endoderm. To gain insights into this process, we use micropatterning together with FGF and the WNT pathway activator CHIR9901 to guide the development of human embryonic stem cells into reproducible patterns of APS cell fates. We show that CHIR9901 dosage dictates the downstream dynamics of endogenous TGFβ signalling, which in turn controls cell fate decisions. While sustained NODAL signalling defines endoderm and NODAL inhibition is imperative for neuro-mesoderm emergence, timely inhibition of NODAL signalling with spatial confinement potentiates WNT activity and enables us to generate NotoPs efficiently. Our work elucidates the signalling regimes underpinning NotoP emergence and provides insights into the regulatory mechanisms controlling the balance of APS cell fates during gastrulation.

## Teaser

**Summary:** Culture of human embryonic stem cells on a micropattern system reveals that timely NODAL inhibition potentiates WNT signalling and directs anterior primitive streak progenitors to the notochord lineage.

## INTRODUCTION

**Fig. 1.**
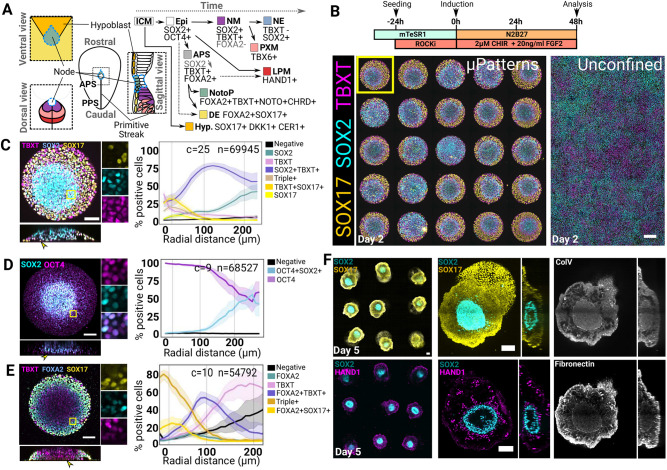
**Geometrical confinement elicits radial patterning of endoderm- and mesoderm-biased cell fates.** (A) Schematic showing the putative organisation and hierarchies of cell fates around the node in a human embryo at the end of gastrulation. The sagittal view shows the domain surrounding the node along the midline. Extra-embryonic structures are omitted (apart from the hypoblast). (B) Top: protocol used for the data shown in C-E. Bottom: confocal maximum projections showing unconstrained and micropatterned (μPatterns) cultures. Notice some variability between colonies, likely due to initial differences in seeding density across the well. The colony shown in C is indicated with a yellow outline. Scale bar: 200 µm. (C-E) Maximum projections of micropatterned colonies stained at 48 h (left) and radial profiles of cell type abundance (right). Insets show single confocal planes at higher magnification. Scale bars: 100 µm. Yellow arrowheads show SOX17^+^ or SOX2^−^ cells in the *z*-axis projections. Number of analysed colonies (c) and number of analysed nuclei (n) are shown at the top. Shaded area represents 95% confidence interval. Plots show one experiment representative of at least two independent experiments. (F) Colonies stained at day 5 of differentiation. Images are maximum projections except for the selected colony stained for HAND1, collagen V (ColV) and fibronectin for which a *z*-slice was selected to better show the localisation of the positive signal. Scale bars: 100 µm. APS, anterior primitive streak; DE, definitive endoderm; Epi, epiblast; Hyp., hypoblast; ICM, inner cell mass; LPM, lateral plate mesoderm; NE, neurectoderm; NM, neuro-mesoderm; NotoP, notochord progenitors; PPS, posterior primitive streak; PXM, paraxial mesoderm.

In vertebrate embryos, the tissues of the posterior axis, including the spinal cord, cartilage, bones and muscles of the spine, as well as the gut, are all laid down progressively in an anterior-to-posterior direction. This evolutionarily conserved process, termed axial elongation (reviewed by Henrique et al., 2015; Martins-Costa et al., 2024; Neijts et al., 2014; Wymeersch et al., 2021), is governed by lineage-restricted progenitors emerging during gastrulation in the anterior primitive streak (APS) (Fig. 1A).

Among these, notochord progenitors (NotoPs) generate the notochord, a cylindrical structure forming along the ventral midline in chordate embryos (Balmer et al., 2016; Stemple, 2005). The notochord ensures essential functions during development, both as a source of localised signalling molecules necessary for the proper patterning of adjacent tissues (Streit and Stern, 1999) and as a mechanical structure contributing to the straightness of the rostro-caudal axis as the embryo elongates (Bagnat and Gray, 2020; McLaren and Steventon, 2021). Eventually, notochord cells contribute to the nucleus pulposus (Choi et al., 2008; McCann et al., 2012), the central region of intervertebral discs ensuring the homeostasis of the surrounding fibro-cartilagenous tissue (reviewed by Wise et al., 2020).

Previous work in mouse embryos has shown that notochord specification requires the cooperation of WNT and NODAL signalling (Dunn et al., 2004; Lickert et al., 2002; Merrill et al., 2004; Vincent et al., 2003; Yamamoto et al., 2001). However, despite previous efforts (Colombier et al., 2020; Diaz-Hernandez et al., 2020; Winzi et al., 2011; Zhang et al., 2020), the derivation of NotoPs from pluripotent stem cells remains inefficient unless transcription factors regulating the notochord fate are overexpressed, and even these conditions lead to mixed population of cells (Schifferl et al., 2023; Warin et al., 2024). Furthermore, apart from one exception (Xu et al., 2021), 3D organoids that mimic axial elongation also lack notochord even though WNT and NODAL signalling are both active in these *in vitro* models (Beccari et al., 2018; Cermola et al., 2021; Moris et al., 2020; Turner et al., 2017), suggesting that unknown cues are needed for notochord specification.

This gap in understanding stems from the relative rarity of NotoPs in the embryo and from the complexity and rapidity of the cell fate decisions during gastrulation. In mouse embryos, NotoPs emerge in close proximity to both the definitive endoderm (Beddington and Robertson, 1989; Scheibner et al., 2021), and neuro-mesoderm (NM)-fated cells (Forlani et al., 2003; Tzouanacou et al., 2009). All three populations, endoderm, NotoPs and NM-fated cells, eventually form a progenitor zone, organised along the antero-posterior axis of the embryo (Fig. 1A). On the anterior-most region of the primitive streak, endoderm progenitors undergo partial epithelial-to-mesenchymal transition (EMT) and intercalate into the underlying visceral endoderm/hypoblast to form the gut endoderm (Kwon et al., 2008; Scheibner et al., 2021; Simpson et al., 2024; Viotti et al., 2014). NotoPs establish the ventral epithelial layer of the node, demarcating the rostral and caudal regions of the embryo (de Bakker et al., 2016; de Bree et al., 2018; Kinder et al., 2001). NM-fated cells populate the epithelial space directly adjacent and posterior to the node and together with NotoPs form the progenitor growth zone that fuels axial elongation (Abdelkhalek et al., 2004; Wymeersch et al., 2019). How the patterning and balanced proportion of these populations is established is not understood.

Here, we set out to identify the signalling requirements distinguishing NotoPs from other APS cell fates using human embryonic stem cells (hESCs) confined on micropatterns as a model system. Starting with a NM-inducing medium containing FGF and the WNT pathway activator CHIR99021 (CHIR) (Cohen and Goedert, 2004), we show that CHIR dosage dictates endogenous NODAL signalling dynamics, which in turn biases cells to endoderm and mesoderm lineages. We show that NODAL inhibition potentiates WNT signalling and, while continuous NODAL inhibition is imperative for NM emergence, delayed NODAL inhibition efficiently specifies endoderm-competent progenitors to NotoPs. Our study uncovers signalling cross-talk and dynamics that correlate with key lineage restrictions in the APS and identifies the path to the notochord lineage.

## RESULTS

### hESC colony confinement directs patterning of mesendoderm-biased cell fates

In mice, NotoPs emerge near NM-fated cells during gastrulation within a small, confined region of the embryo (Forlani et al., 2003). Furthermore, single-cell RNA-sequencing analysis has suggested that rare node-like cells arise in pluripotent cell-derived NM progenitors (NMPs) (Edri et al., 2019). Therefore, we hypothesised that combining an NMP derivation medium (Frith and Tsakiridis, 2019; Gouti et al., 2014) with geometrical confinement (Blin, 2021) could elicit the self-organisation of hESCs into distinct domains of APS cell fates, including NotoPs.

To test this idea, we stained the cells 48 h post-CHIR and FGF induction for the NMP markers TBXT and SOX2, and included the endodermal marker SOX17 (Kanai-Azuma et al., 2002; Viotti et al., 2014) as a proxy for other APS cell fates that do not normally appear in NMP differentiation monolayers (Frith and Tsakiridis, 2019) (Fig. 1B). In line with previous reports (Frith et al., 2018; Gouti et al., 2014), cells cultured in conventional 2D dishes co-expressed TBXT and SOX2 and remained negative for SOX17 (Fig. 1B). In sharp contrast, cells grown on micropatterns consistently formed radially organised domains of marker expression (Fig. 1B,C, Fig. S1A): SOX17 was preferentially expressed at the periphery, SOX2-only cells were found in the centre and at the top of the colony and SOX2^+^TBXT^+^ cells were most abundant around 100 µm from the colony border. We confirmed that this phenomenon was reproducible across several human pluripotent cell lines with some degree of inter-line variability (Fig. S1B).

We next tested whether the proportion of cell fates on micropatterns is affected by colony size (Fig. S2). Using our established quantitative immunofluorescence (qIF) pipeline (Blin et al., 2019; Wisniewski et al., 2019), we measured the proportion of each individual population (Fig. S2B) and plotted these proportions as a function of the radial distance from the colony edge (Fig. S2C). We found that all three main domains, i.e. SOX17^+^, TBXT^+^SOX2^+^ and SOX2-only domains, remained located at a consistent distance from the colony edge across all diameters except for colonies smaller than 320 µm for which this rule did not apply as strictly. Incidentally, the central domain of SOX2-expressing cells increased in size proportionally with colony diameter, effectively increasing the percentage of SOX2-only cells and suggesting that the radial organisation of cell fates may be boundary driven as reported in other micropatterned colony systems (Etoc et al., 2016; Martyn et al., 2019; Warmflash et al., 2014). We decided to use 500 µm colonies thereafter because this diameter offered a good compromise for analysis and imaging.

We next investigated the developmental state of the cells forming the central SOX2 domain. In mouse embryos, SOX2 is initially co-expressed with OCT4 (POU5F1) in the pluripotent epiblast and remains expressed in the developing neurectoderm while OCT4 becomes progressively lost as the cells exit pluripotency (Avilion et al., 2003; Osorno et al., 2012). In our colonies, OCT4 was still expressed in the central SOX2 domain indicating that the cells had not yet exited pluripotency (Fig. 1D).

Next, we tested for the presence of NotoPs. We first looked for the co-expression of FOXA2 and TBXT, which are both essential for the development of the notochord (Ang and Rossant, 1994; Lolas et al., 2014; Tamplin et al., 2011; Yamanaka et al., 2007). FOXA2^+^ cells were found all around the colony spanning a domain of approximately 100 µm from the edge (Fig. 1E, Fig. S3). This domain could be divided into a SOX17^+^ outer domain indicative of the endodermal fate and a FOXA2^+^TBXT^+^ inner domain colocalising with the SOX2^+^TBXT^+^ domain shown in Fig. 1C. Since these markers are also transiently co-expressed in the nascent mesendoderm during gastrulation (Burtscher and Lickert, 2009), we performed fluorescence *in situ* hybridisation (FISH) against the NotoP-specific marker *NOTO* (Abdelkhalek et al., 2004; Plouhinec et al., 2004) but did not observe any positive cells for this marker (see Fig. 6A), suggesting that the TBXT^+^FOXA2^+^ cells in these colonies likely represent an early mesendoderm population at 48 h.

To characterise further the lineages emerging in micropatterned colonies, we repeated the protocol from Fig. 1B and cultured the cells for an additional 3 days in unsupplemented N2B27 (Fig. 1F). After 5 days, the colonies established a 3D structure composed of a cavity-comprising SOX2^+^ core surrounded by a mass of SOX17^+^ endodermal cells. We also found clusters of HAND1^+^ cells, indicative of lateral plate mesoderm (LPM) or extra-embryonic mesoderm (ExM) (Pham et al., 2022). However, we were unable to find evidence of notochord-like cells using TBXT, FOXA2 and SOX9, which are normally co-expressed in the notochord (Bagheri-Fam et al., 2006), confirming that the TBXT^+^FOXA2^+^ population identified at 48 h failed to engage in the notochord lineage. We did not find TBXT^+^SOX2^+^ cells in these structures either, suggesting that NMP-like cells were also absent.

The 3D multi-tissue architecture observed here was remarkably consistent across colonies (Fig. 1F). Colonies adopted a 3D organisation as early as 48 h with the formation of a dome-like structure in which SOX2^+^ cells were elevated in comparison to other cell types (Fig. 1C,D, Figs S1A and S2A). We consistently observed that SOX17^+^ cells, abundant at the periphery, also lined the bottom of the colony (arrowheads in Fig. 1C,E and Figs S1A and S2A), perhaps reflecting the behaviour of the nascent endoderm *in vivo*, which undergoes partial EMT to form the gut endoderm epithelium during gastrulation (Kwon et al., 2008; Scheibner et al., 2021; Viotti et al., 2014). Staining for collagen V and fibronectin at day 5 (Fig. 1F), two extracellular matrix proteins expressed in the endoderm and mesoderm in human embryos (Zhao et al., 2024 preprint), revealed that the cells secreted their own extracellular matrix. Both proteins formed a basal membrane surrounding the SOX2^+^ domain while fibronectin also formed a complex network inside the endodermal domain. These observations indicate that the cells organised their own extracellular matrix, which likely contributed to the overall morphogenetic process observed in the colonies.

Altogether, these initial experiments allowed us to establish an *in vitro* system in which hESCs become organised into patterns of cell fates undergoing complex morphogenesis over time. Although this system provides a good starting point, neither NMP-like cells nor NotoPs emerged on micropatterns despite the use of an NMP-inducing medium, raising the question of what endogenous cues may deflect the cells from these axial cell fates.

### NODAL signalling precedes the loss of axial cell fate markers and the emergence of mesoderm and endoderm

**Fig. 2.**
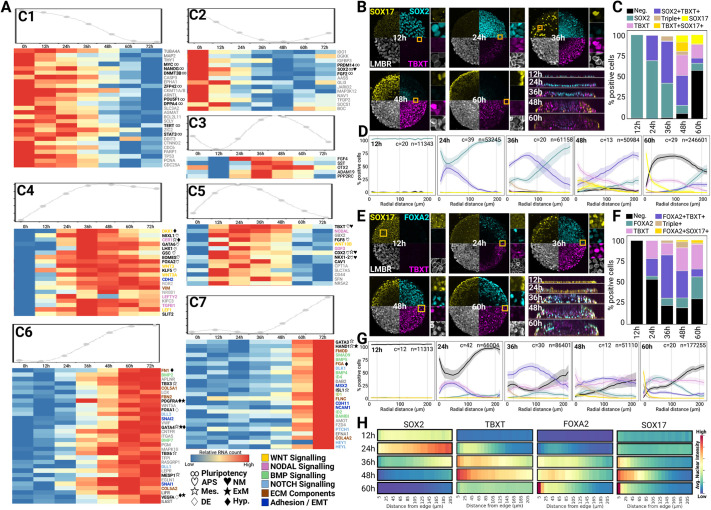
**Dynamics of TGFβ signalling correlate with the emergence of endoderm and mesoderm.** (A) NanoString time course analysis. Cells were treated as in Fig. 1B. The model spline is shown at the top of each heatmap. Genes are ordered from top to bottom in decreasing order of maximum differential expression. Heatmap colours represent mRNA counts normalised on a per gene basis. Coloured text and symbols relate to involvement in processes and tissues, as indicated in the key. (B-H) Time course analysis of 500 µm colonies by quantitative immunofluorescence. (B,E) Montage of confocal maximum projections with insets showing a single confocal plane at higher magnification. *z*-projections are also shown. (C,D,F,G) Stacked bar charts (C,F) and radial profiles (D,G) of cell type abundance. Number of analysed colonies (c) and number of analysed nuclei (n) are shown at the top of the profiles, and shaded area represents 95% confidence interval. (H) Average nuclear intensity along the radial distance. The data show a representative experiment from two independent replicates. APS, anterior primitive streak; DE, definitive endoderm; ECM, extracellular matrix; EMT, epithelial-to-mesenchymal transition; ExM, extra-embryonic mesoderm; Hyp., hypoblast; NM, neuro-mesoderm; Mes., mesoderm.

To gain insights into the mechanisms of cell fate patterning in our colonies, we used NanoString to record gene expression over time (Fig. 2A). We used a panel of probes consisting of the 780 genes included in the standard hESC gene panel together with 30 additional custom probes (listed in Table S1). These 810 probes covered a wide array of genes involved in differentiation, metabolism, signalling pathways and the cell cycle. We used the Bioconductor package moanin (Varoquaux and Purdom, 2020) to group individual genes based on their temporal profile (see Materials and Methods) and identified seven clusters (Fig. 2A). Genes in clusters 1 and 2 were progressively downregulated and included pluripotency markers such as *MYC*, *OCT4*, *DPPA4*, *DNMT3B* and *ZFP42*. We found *NANOG* to be highest at around 36 h and then lost rapidly, consistent with the fact that *NANOG* is re-expressed in the posterior epiblast at the onset of gastrulation (Mulas et al., 2018; Hart et al., 2004; Osorno et al., 2012; Simpson et al., 2024). Genes in cluster 4 and 5 initiated expression as early as 12 h post-induction and either plateaued (cluster 4) or peaked at 36 h (cluster 5). These clusters comprised genes associated with the APS in mice, including *MIXL1* (Hart et al., 2002), *LHX1* (Costello et al., 2015), *EOMES* (Costello et al., 2011), *GSC*, *CDX2*, *FOXA2* (Burtscher and Lickert, 2009), *KLF5* (Aksoy et al., 2014) as well as the APS and NM fate-associated gene *NKX1-2* (Albors et al., 2018). Remarkably, we found a peak of expression of *SLIT2* and *CAV1* at 36 h, recently reported as markers of human embryonic notochord (Paillat et al., 2023; Warin et al., 2024). However, NM-fate- and NotoP-associated genes decreased over time, confirming that axial progenitors failed to emerge.

We confirmed this using qIF (Fig. 2B-F, Fig. S4A,B). While TBXT^+^SOX2^+^ cells (Fig. 2B-D) and FOXA2^+^TBXT^+^ cells (Fig. 2E-G) appeared at 24 h at the periphery of the colonies and increased in proportion until 36 h, these populations were progressively lost at later time points in favour of cells expressing other marker combinations. Notably, we observed the emergence of SOX2^+^TBXT^+^SOX17^+^ and FOXA2^+^TBXT^+^SOX17^+^ triple-positive cells at 36 h, indicating that TBXT^+^SOX2^+^ and FOXA2^+^TBXT^+^ cells found before 36 h are neither NM-fated nor notochord but are instead likely to represent mesoderm and endoderm precursors. Indeed, in the NanoString experiment, evidence of endoderm and mesoderm differentiation was visible at late time points in clusters 6 and 7 (Fig. 2A) with upregulation of the definitive endoderm (DE) gene *FOXA1* (Ang and Rossant, 1994), the mesodermal markers *TBX3* and *GATA3* (Gunne-Braden et al., 2020; Mackinlay et al., 2021), the anterior mesoderm markers *MESP1* (Lescroart et al., 2018; Probst et al., 2021; Saga et al., 1999) and *HAND1* (Barnes et al., 2010) as well as the EMT markers *SLUG* (*SNAI2*), *SNAIL* (*SNAI1*) and *MSX2*. Several genes expressed at 72 h also mark ExM in primates *in vitro* and *in vivo*, such as *GATA4*, *HAND1*, *PDGFRA* (Bergmann et al., 2022; Oldak et al., 2023; Pham et al., 2022) and *VEGFA* (Zhao et al., 2024 preprint). We also found genes reported as hypoblast markers, such as *PDGFRA*, *FN1* and *FGA* (Mackinlay et al., 2021; Zhao et al., 2024 preprint), indicating that extra-embryonic lineages may be arising on micropatterns.

Overall, these data showed that cells on micropatterns initially followed the route towards axial cell fates (i.e. expressing APS markers) but eventually differentiate towards alternative lineages including DE/hypoblast and LPM/ExM.

We thus turned our focus towards endogenous signalling pathways that may explain why NotoPs and NMP-like cells fail to emerge in our colonies (Fig. 2A). As expected, the WNT target gene *LEF1* (Cadigan and Waterman, 2012) was upregulated as early as 12 h and remained highly expressed thereafter (cluster 4). BMP signalling components were upregulated at late time points (clusters 6 and 7) together with the BMP-responsive genes *TBX3* and *GATA3* (Gunne-Braden et al., 2020). More importantly, *NODAL*, a known driver of mesendodermal specification (Robertson, 2014) peaked at 24 h, and then decreased progressively (Fig. 2A). FISH analysis confirmed *NODAL* expression alongside the genes encoding its antagonists, *LEFTY2* and *CER1* (Fig. S4C). Interestingly, *CER1* and the WNT antagonist *DKK1*, both expressed in the hypoblast of primate embryos (Bergmann et al., 2022) were expressed in a domain that overlapped spatially and temporally with the SOX17 domain (Fig. 2B, Fig. S4C), further indicating that at least a fraction of the endoderm emerging in micropattern colonies may adopt a hypoblast identity.

Altogether, our data show that differentiation on micropattern follows a coherent developmental programme governed by an endogenous network of NODAL/TGFβ signalling. The fact that NODAL peaks at a time that shortly precedes the loss of axial markers places NODAL as a candidate pathway that may prevent NM and notochord emergence.

### Constant NODAL inhibition safeguards NMP-like cells but fails to drive NotoPs

**Fig. 3.**
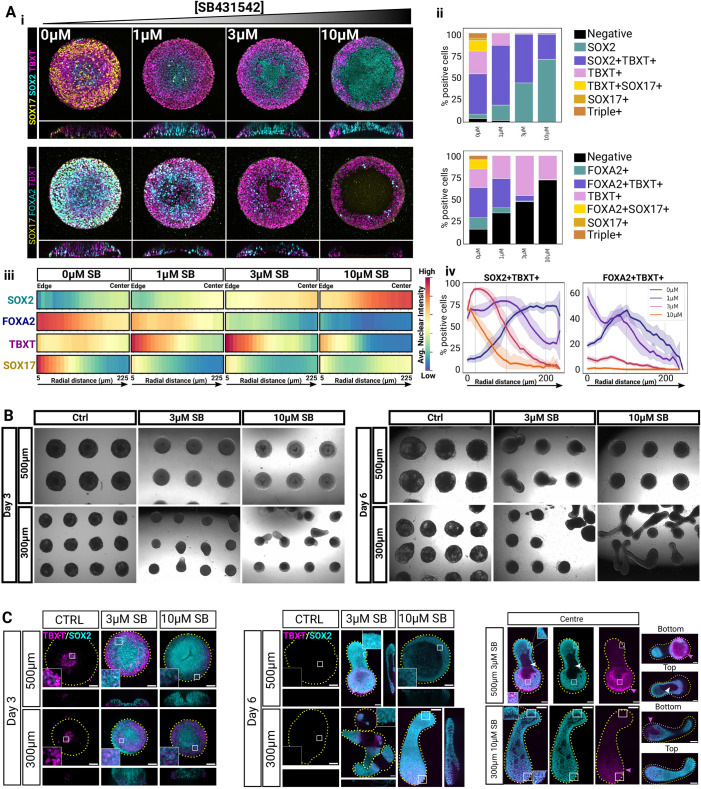
**Nodal inhibition safeguards NMP-like cells and tissue elongation.** (A) SB dose response in 500 µm colonies fixed at 48 h. (i) Maximum projections of confocal *z*-stacks (top) and *z*-projection (bottom). (ii) Stacked bar plots of cell type abundance. (iii) Radial profiles of average nuclear marker intensity scaled between 0 and 1 on a per-marker basis. (iv) Radial profiles of cell type abundance. Note that we sometimes observed detachment in the centre of the pattern during fixation (see 10 µM SB condition). This does not reflect colony morphology prior to fixation. (B) Brightfield images of hESC colonies grown on 500 µm or 300 µm micropatterns treated with CHIR/FGF and varying doses of SB431542. (C) Maximum projections of confocal *z*-stacks showing representative micropatterned colonies stained for TBXT and SOX2 (left and middle panels). The panel on the right shows selected single confocal planes of elongated structures to better show the cavity (white arrowhead) and the location of the TBXT domain (magenta arrowhead). Insets show boxed areas at higher magnification. Scale bars: 100 µm. Images are representative of three independent experiments.

Our results raise the possibility that endogenous NODAL signalling deflects cell fates towards mesoderm and endoderm lineages. To confirm this hypothesis, we titrated NODAL activity using increasing doses of the NODAL receptor inhibitor SB431542 (SB) (Inman et al., 2002) (Fig. 3A). We observed that even a low amount of SB was sufficient to strongly reduce the proportion of SOX17^+^FOXA2^+^ cells, confirming that endogenous NODAL signalling activity is indeed required for endoderm specification (Fig. 3A, 1 µM). However, FOXA2^+^TBXT^+^ and SOX17^−^ cells present in this condition remained negative for NOTO (see Fig. 6A), indicating that constant NODAL inhibition did not support NotoP emergence. By contrast, a high percentage of SOX2^+^TBXT^+^ and FOXA2^−^ cells was induced at the periphery upon NODAL inhibition (Fig. 3A, 3 µM and 10 µM). We thus hypothesised that these cells may now be engaging in the NMP fate and could therefore drive elongation.

To test this, we cultured the cells for 3 days in CHIR/FGF and an additional 3 days in unsupplemented N2B27 with and without SB added throughout (Fig. 3B,C). Since previous work with 3D models of axial elongation showed that the starting number of cells is crucial for elongation (Beccari et al., 2018; Martins et al., 2020; Moris et al., 2020; Olmsted and Paluh, 2021; Sanaki-Matsumiya et al., 2022; Turner et al., 2017; Veenvliet et al., 2020), we also tested two different colony diameters (500 µm and 300 µm). To our surprise, competence to elongate was a function of both colony diameter and SB concentration, with elongation occurring consistently in two conditions: 79% of colonies elongated at 3 µM SB on 500 µm micropatterns and 96% elongated at 10 µM SB on 300 µm micropatterns (Fig. 3B, Fig. S5). We showed earlier that SOX2^+^TBXT^+^ cells were almost completely lost without SB treatment (Fig. 2C). We thus checked whether SOX2^+^TBXT^+^ cells could be maintained with NODAL inhibition. Indeed, elongation correlated with the conditions favouring the highest abundance of SOX2^+^TBXT^+^ at day 3 (Fig. 3C), indicating that adequate NODAL suppression and colony size combinatorially define NMP-like cell emergence and uniaxial growth on micropatterns. Interestingly, SOX2^+^TBXT^+^ cells were located where the cells initially touched the pattern (Fig. 3C). The predictable location of TBXT-expressing cells in this system is consistent with previous work showing that cell–substrate contact dictates symmetry breaking in 3D aggregates (Sagy et al., 2019).

Together, these results demonstrate that continuous suppression of NODAL signalling is necessary for the specification of NMP-like cells but insufficient for NotoP emergence.

### Small variations in CHIR dosage lead to radically distinct cell fate patterning outcomes

**Fig. 4.**
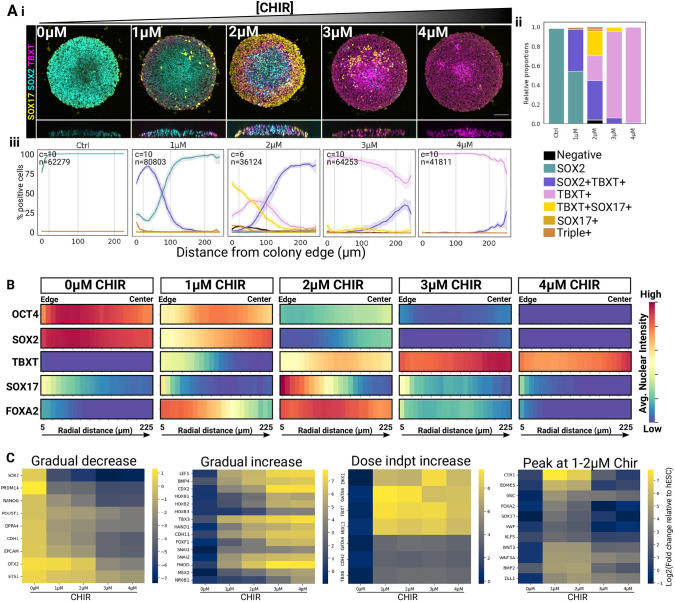
**Cell fate patterning does not correlate linearly with CHIR dosage.** (A) CHIR dose response in 500 µm colonies fixed at 48 h. (i) Maximum projections of confocal *z*-stacks and *z*-projections. (ii,iii) Stacked bar plot (ii) and radial profiles (iii) of cell type abundance for each CHIR concentration. Number of analysed colonies (c) and number of analysed nuclei (n) are shown. Shaded area represents 95% confidence interval. (B) Heatmaps showing the radial profile of average nuclear marker intensity scaled between 0 and 1 on a per-marker basis. Quantifications show a representative experiment from two independent biological replicates. (C) NanoString analysis of 500 µm colonies treated with increasing doses of CHIR for 48 h. Colour scale represents log2 ratio of mRNA count in the sample relative to hESCs. indpt, independent.

None of the tested conditions so far allowed us to find NOTO^+^ cells using FISH staining (not shown) raising the question of what other cues would be needed for NotoP emergence. To gain further insights, we next tested a range of CHIR concentrations and monitored markers of cell fates 48 h post-induction by qIF (Fig. 4A,B) and NanoString analysis (Fig. 4C). Without CHIR, all the cells remained OCT4^+^SOX2^+^ and negative for TBXT, SOX17 and FOXA2 (Fig. 4A,B, Fig. S6A,B) showing that exogenous stimulation of the WNT pathway is required to initiate differentiation. More importantly, we found a clear dose-dependent effect of CHIR on the levels and spatial distributions of cell fate markers: the percentage of SOX2^+^ cells was negatively correlated with CHIR concentration while the percentage of TBXT^+^ cells increased proportionally, in line with the fact that TBXT is a known direct target of the WNT/β-catenin pathway (Arnold et al., 2000). Conversely, SOX17 and FOXA2 behaved non-monotonically, being expressed at intermediate CHIR concentrations and rare or absent at higher concentrations (Fig. 4A, Fig. S6B). High doses of CHIR induced instead the expression of the LPM/ExM marker HAND1 (Fig. S6C). Importantly, the non-monotonic effect of CHIR dosage on cell fate markers was valid across four different cell lines (Fig. S7). Our NanoString analysis supported these results with a peak of endodermal markers at 1 and 2 µM CHIR while colonies treated with the highest doses of CHIR expressed the LPM marker HAND1 (Fig. 4C). These data suggest that endodermal cells are replaced by more posterior mesodermal fates at high CHIR concentration.

Together, our data show a complex, non-linear dose-dependent action of CHIR on cell fates, with intermediate doses of CHIR inducing endodermal cell fates predominantly, and higher doses inducing more posterior (TBXT^+^ and FOXA2^−^) mesodermal fates.

### CHIR dosage correlates with distinct downstream NODAL signalling dynamics

**Fig. 5.**
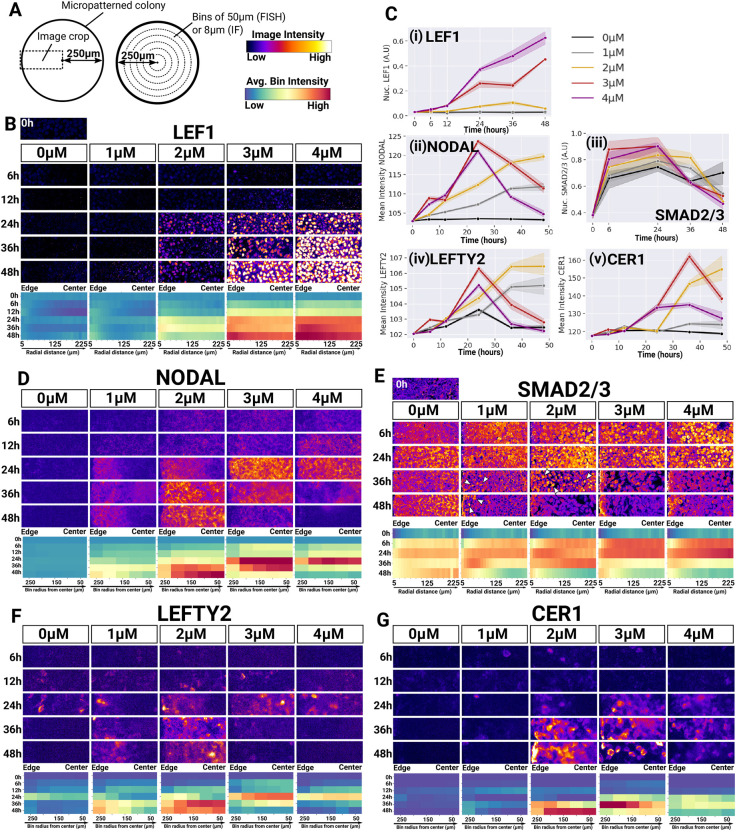
**CHIR dosage defines downstream dynamics of NODAL signalling.** (A) Diagrams illustrating the position of the image crops shown in B and D-G (left) and the organisation of the bins shown in heatmaps (middle). (B-G) Time course analysis of WNT and NODAL signalling in 500 µm colonies across CHIR concentrations. B and E show immunofluorescence analysis of nuclear LEF1 and nuclear SMAD2/3 levels, respectively. Images show selected *z*-slices across a confocal *z*-stack. D, F and G show FISH analysis of *NODAL*, *LEFTY2* and *CER1* transcript levels. Images are 2D widefield images. For B,D-G, the radial profiles of signal intensities over time is shown as heatmaps. (C) Temporal profiles of protein (LEF1 and SMAD2/3) and transcript (NODAL, LEFTY2 and CER1) levels averaged across entire colonies. Lines indicate the mean average expression across colonies, and the shaded area indicates the 95% confidence interval. The figure is representative of two independent experiments. A.U., arbitrary unit.

Our previous results raise the possibility of a non-linear effect of CHIR concentration on downstream signalling activity, which would in turn define cell fate outcomes on micropatterns. We first checked the spatiotemporal dynamics of the canonical WNT pathway using the target gene *LEF1* (Fig. 5A-C). LEF1 expression increased monotonically over time at a rate proportional to CHIR concentration without any clear pattern of expression along the colony radius. This showed that the cells were all equally competent to respond proportionally to CHIR dosage.

**Fig. 6.**
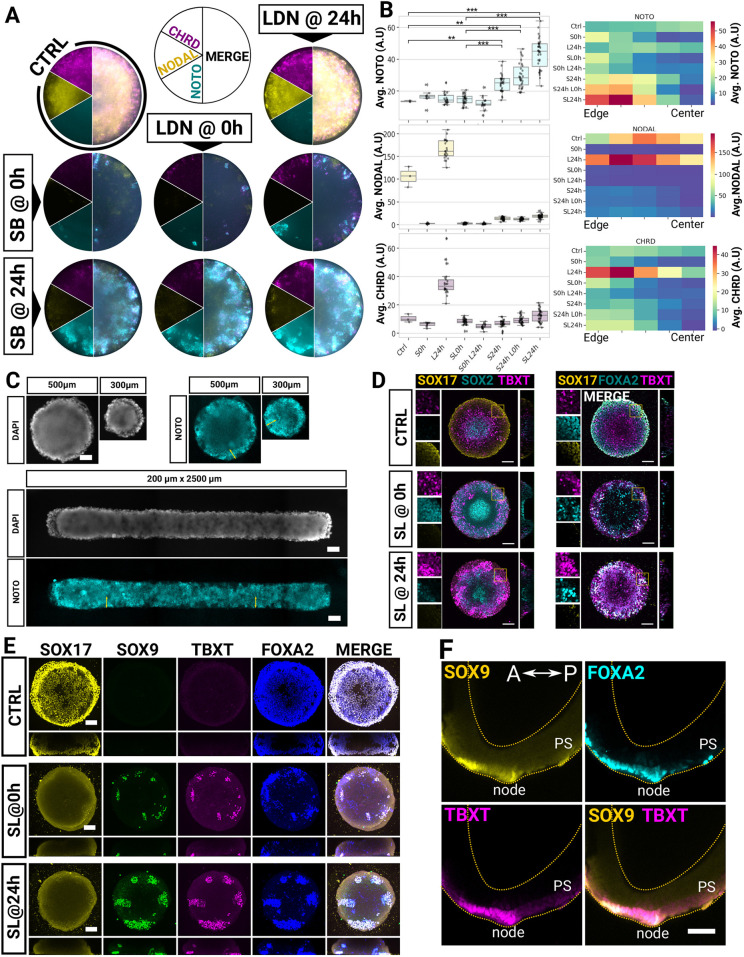
**Timely TGFβ inhibition directs NotoPs on micropatterns.** (A) FISH staining for *NODAL*, *CHRD* and *NOTO* in 500 µm micropattern colonies induced with 2 µM CHIR and 20 ng/ml FGF2 and fixed at 48 h post-induction. Timed treatment with the NODAL or BMP inhibitors is indicated. (B) Box plots of average signal intensities in each colony (left) and heatmaps of binned signal intensities along the radial distance (right). **P<0.05; ***P<0.001 (unpaired *t*-tests). Box limits indicate the 1st and 3rd quartiles of the distribution, horizontal bars indicate the median and whiskers show 1.5 times the interquartile range (delimited by the box). Points outside this range are considered outliers (diamonds). (C) Widefield images of micropatterned colonies (NAS2) stained by FISH at 48 h with SL added at 24 h. Yellow arrows indicate a 100 µm domain from the edge. (D) *z*-stack maximum projections of colonies stained 2 days post-CHIR/FGF induction and SB and LDN (SL) treatment as indicated. Insets show boxed areas at higher magnification. (E) *z*-stack maximum projections of colonies grown for 3 days in 2 µM CHIR and 20 ng/ml FGF-containing medium and an additional 3 days in unsupplemented N2B27 with SL treatment as indicated. (F) Confocal sagittal plane of a whole-mount immunostained early bud mouse embryo. Notice the SOX9 expression in the crown cells of the node and the nascent notochord. Scale bars: 100 µm. A.U., arbitrary unit.

We thus investigated the spatiotemporal dynamics of NODAL signalling across CHIR concentrations (Fig. 5C-E). During the first 24 h, the rate of increase in *NODAL* transcripts correlated with the dose of CHIR. However, whereas *NODAL* kept increasing after 24 h at 1 and 2 µM CHIR, *NODAL* expression rapidly decreased with 3 µM CHIR and dropped even faster with 4 µM (Fig. 5Cii,D). Nuclear SMAD2/3 levels (the downstream signalling effector of the pathway) were consistent with the temporal profiles of *NODAL* expression: nuclear SMAD2/3 first increased proportionally to CHIR concentrations at early time points and then dropped earlier at 3 and 4 µM CHIR than at 1 and 2 µM CHIR (Fig. 5Ciii,E) confirming the non-linear dependence of NODAL signalling to CHIR dosage.

When looking closely at the spatiotemporal pattern of NODAL signalling, we observed that *NODAL* expression was evenly distributed during the first 12 h at all CHIR concentrations (Fig. 5D), consistent with the notion that all the cells responded to CHIR by activating NODAL expression. However, at 1 and 2 µM CHIR, a radial gradient of *NODAL* expression, highest at the periphery, became apparent at 24 h in 1 and 2 µM CHIR. This domain expanded only slightly towards the centre at 1 µM CHIR whereas it reached the centre as soon as 36 h at 2 µM CHIR. Importantly, the spatiotemporal pattern of *NODAL* expression in 2 µM colonies correlated well with the pattern of FOXA2 and TBXT expression found earlier (Fig. 2E-H), further reinforcing the notion that endogenous NODAL dictates cell fate patterning in micropatterned colonies. Of note, a wave of *NODAL* expression was not apparent at 3 µM and 4 µM CHIR, perhaps because radial expression occurred too rapidly between 12 h and 24 h for our experiment to capture this process.

Somewhat consistently with the spatiotemporal dynamics of NODAL expression, some cells with nuclear SMAD2/3 persisted until 36 h at 2 µM CHIR and until 48 h at the periphery of 1 µM-treated colonies (Fig. 5E). However, nuclear SMAD2/3 did not strictly follow *NODAL* expression. For example, nuclear SMAD2/3 increased sharply as early as 6 h post-CHIR treatment – before the peak of *NODAL* expression (Fig. 5C-E) – and was heterogeneous inside the domain of *NODAL* expression (Fig. 5D,E). These observations may reflect the complex cell-autonomous regulation layers of the pathway (Bohn et al., 2023; Heemskerk et al., 2019; Varelas et al., 2008). Furthermore, *NODAL* expression dynamics (Fig. 5Cii) indicated the existence of a negative feedback that occurs faster with higher doses of CHIR. Inhibitors of the pathway include LEFTY2 and CER1 (Aykul et al., 2015). *LEFTY2* expression followed closely the spatiotemporal dynamic of *NODAL* expression (Fig. 5F), and *CER1* harboured a similar pattern albeit in a temporally delayed manner (Fig. 5G) supporting the idea that LEFTY2 and CER1 may indeed contribute to the temporal profile of NODAL signalling. However, the decoupling between the level of these inhibitors and the downregulation kinetic of NODAL (with 2 µM CHIR for example) implies that other mechanisms are also at play and it will be interesting to elucidate this in the future.

Together, our results demonstrate that small variations in CHIR concentration induce distinct spatiotemporal dynamics of endogenous NODAL signalling in micropatterned colonies. At high CHIR concentration (3 and 4 µM), *NODAL* expression is rapidly extinguished and a strong WNT signalling activity induces mesodermal TBXT^+^ cells across the entire colony. Treatment with 1 and 2 µM CHIR induces low to moderate levels of LEF1 and an inward wave of *NODAL* expression initiated from the boundary that persists until 48 h. The existence of such a wave indicates that cells located at the periphery experience an earlier, and therefore prolonged, exposure to NODAL signalling compared to the cells at the centre. This spatiotemporal pattern correlates with the positioning of FOXA2^+^TBXT^+^ cells at 24 h and the later emergence of endodermal SOX17^+^ cells (Fig. 2E-G). SOX17 expression is likely the result of continuous exposure of FOXA2^+^TBXT^+^ cells to NODAL after 24 h. These observations raise the possibility that redirecting FOXA2^+^TBXT^+^ cells towards the notochord could be achieved if continuous exposure to NODAL is prevented.

### Abrupt NODAL and BMP inhibition is required for NotoP specification

We next turned to the question of how to modify signalling further in order to achieve NotoP differentiation. Specification of NotoPs requires the cooperation of WNT and NODAL signals in embryos (Lickert et al., 2002; Simpson et al., 2024; Vincent et al., 2003; Yamamoto et al., 2001). However, our previous results show that varying WNT activity alone is not sufficient to elicit NotoPs despite the consequences of WNT activity on downstream NODAL signalling. Since BMP signalling inhibits notochord specification (Yasuo and Lemaire, 2001) and our NanoString (Figs 2 and 4C) and FISH (Fig. S8) data showed that BMP is indeed expressed in our system (Figs 2 and 4C), most likely downstream of NODAL (Chhabra et al., 2019; Repina et al., 2023), we hypothesised that the NODAL and BMP signalling dynamics established spontaneously within our colonies is inadequate for NotoP emergence and that a tight exogenous control of these signals is instead necessary.

We thus inhibited NODAL and BMP signalling using small molecule inhibitors added either throughout differentiation or at 24 h when a peak of *NODAL* expression was observed and when putative precursors of NotoPs may be present (when TBXT^+^FOXA2^+^ cells were already present and SOX17^+^ cells were still absent). We monitored NotoP emergence using FISH against the NotoP marker *NOTO* and *CHRD*, a BMP inhibitor expressed in the APS and rapidly restricted to the node in mice (Bachiller et al., 2000). As expected, we found high levels of *NODAL* expression but no *NOTO* signal in the control (Fig. 6A,B). We also found a broad domain of *CHRD* transcripts across the colony, probably reflecting their APS identity. Treatment with 0.1 µM of the BMP inhibitor LDN 193189 (LDN) added at 24 h had no effect on *NOTO* but increased *CHRD* and *NODAL* expression, indicating that BMP signalling may be one of the factors negatively regulating *NODAL* expression. More importantly, NODAL and BMP inhibition from 0 h abolished *NODAL* expression and resulted in the presence of rare *NOTO*^+^*CHRD*^+^ cells at the colony periphery, indicating that some NotoPs can be specified in this condition. Even more importantly, NODAL inhibition from 24 h onwards induced a large domain of strong *NOTO* and *CHRD* co-expression localised within a 150 µm distance from the colony edge. Addition of LDN at 24 h further potentiated this effect, confirming that BMP signalling inhibits NotoP specification in human cells as well. NotoP emergence was reproducible across all colonies within the experiment (Fig. 6B) as well as with other cell lines (Fig. S9), confirming the robustness of these results. Furthermore, manipulating colony shape to maximise the proportion of cells experiencing edge effects, such as lines of 200 µm width, enabled us to generate colonies covered almost entirely with *NOTO*^+^ cells (Fig. 6C).

Immunofluorescence staining for SOX17, SOX2, TBXT and FOXA2 in these conditions at 48 h (Fig. 6D) showed that endoderm (FOXA2^+^SOX17^+^ cells) was eliminated from colonies treated with SB and LDN (SL) added at 24 h and that instead, colonies contained a large amount of TBXT^+^FOXA2^+^ and SOX17^−^ cells. These results support the notion that FOXA2^+^TBXT^+^ cells formed at 24 h are competent to form either endoderm or NotoPs depending on whether these cells experience sustained NODAL signalling or an abrupt inhibition respectively.

Finally, to determine whether the NOTO^+^ cells found at 48 h were able to form notochord, we cultured the cells for 3 days with CHIR and FGF and then three more days in N2B27 alone with and without SL added at 0 h or 24 h (Fig. 6E). We stained the cells for FOXA2, TBXT and SOX9, which are co-expressed in the node and the nascent notochord in early bud mouse embryos (Fig. 6F), and for SOX17 to distinguish cells that may have formed endoderm. Whereas SOX9 and TBXT were absent from FOXA2^+^SOX17^+^ cells in the control, we found a significant number of FOXA2, TBXT and SOX9 co-expressing cells when SL was added at 24 h.

Together, our data provide evidence that delayed NODAL inhibition in micropattern colonies efficiently produces notochord-competent NotoPs.

### NODAL signalling inhibition potentiates the WNT signalling response

**Fig. 7.**
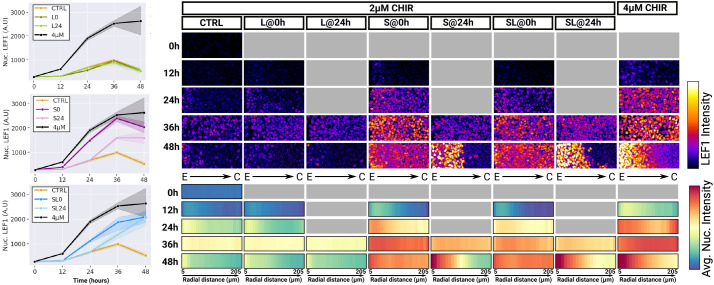
**NODAL inhibition potentiates WNT signalling responsiveness.** Quantitative immunofluorescence time course analysis of LEF1 expression in 500 µm hESC colonies. Line plots show the temporal profiles or average nuclear LEF1 intensity (left). Shaded area represents 95% confidence interval. Images on the right show representative image crops as indicated in Fig. 5A. Radial profiles of signal intensities over time are provided as heatmaps below images. C, centre; E, edge.

Finally, we set out to understand the changes in signalling downstream of TGFβ inhibition that might explain the specification of NotoPs. We used NanoString to find differentially expressed genes in 48 h colonies with and without SB added at 24 h (Fig. S10). When NODAL was inhibited at 24 h, several NOTCH signalling-related genes were upregulated as well as genes associated with anterior identity, such as *GBX2* and *HOXB2*. Importantly, we also found a strong increase in the WNT target gene *LEF1*. To further elucidate how WNT signalling is impacted by TGFβ inhibition and how this may be associated with NotoP emergence, we performed a time course analysis of LEF1 and SMAD2/3 expression in colonies treated with 2 µM CHIR with and without SB alone, with LDN alone, or with a combination of both added at 0 h or 24 h (Fig. 7, Fig. S11). We included a 4 µM CHIR condition to get a comparative measure of LEF1 levels in these colonies. Strikingly, SB alone or SL treatment at 0 h with 2 µM CHIR induced the same increase in LEF1 expression as 4 µM CHIR without TGFβ inhibition. Consistent with this, when SB alone or SL were added at 24 h to 2 µM CHIR-treated colonies, LEF1 expression increased sharply to reach the levels observed in colonies treated with 4 µM CHIR. LDN alone had no noticeable effect on either LEF1 or SMAD2/3 expression (Fig. 7, Fig. S11). These results demonstrate that NODAL inhibition strongly potentiates the cells responsiveness to canonical WNT signalling while BMP inhibition does not. Importantly, LEF1 intensity was the highest at the periphery of the colonies treated with SL added at 24 h in a domain overlapping with the *NOTO* signal seen in Fig. 6A,B, suggesting that derepression of WNT activity when NODAL is inhibited may drive NotoP emergence. Given that 4 µM CHIR treatment generates a high level of WNT activity and a transient peak of *NODAL* expression (Fig. 5), we wondered whether NotoPs could be obtained by inhibiting BMP signalling in 4 µM colonies (Fig. S12). This did not result in any *NOTO*-positive staining and neither did the addition of SL at 24 h in these colonies. These results indicate that a tight control of the temporal profiles of WNT and NODAL signalling is needed for NotoP emergence.

Together, our data clarify how WNT and NODAL signalling cooperate in order to specify the notochordal lineage. WNT and NODAL signalling are initially necessary to induce an early APS cell state, but a timely and abrupt inhibition of NODAL signalling is necessary to maximise WNT signalling response and define NotoPs.

## DISCUSSION

**Fig. 8.**
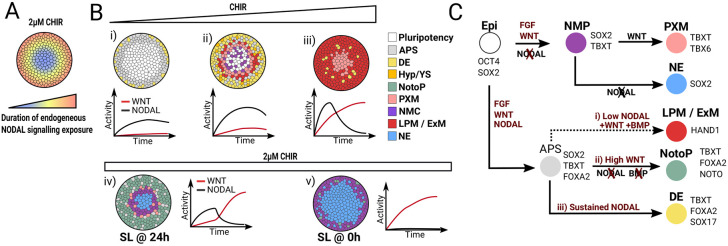
**Graphical summary of cell fate outcomes and associated signalling dynamics.** (A) Schematic illustrating the relative duration of endogenous NODAL during 48 h of differentiation under 2 µM CHIR and 20 ng/ml FGF stimulation. (B) Diagram summarising the spatial organisation of cell fates at 48 h on micropatterns when treated with 20 ng/ml FGF and with 1 µM CHIR (i), 2 µM CHIR (ii, iv, v), 3 µM CHIR (iii) and SB and LDN (SL) added at 24 h (iv) or 0 h (v). The corresponding WNT and NODAL signalling profiles are indicated as line plots alongside each colony. (C) Proposed hierarchy of cell fate lineages and associated signalling requirements. APS, anterior primitive streak; DE, definitive endoderm; Epi, epiblast; ExM, extra-embryonic mesoderm; Hyp., hypoblast; LPM, lateral plate mesoderm; NE, neurectoderm; NMC, neuromesoderm competent; NMP, neuromesodermal progenitor; NotoP, notochord progenitor; PXM, paraxial mesoderm; YS, yolk sac.

The series of lineage restrictions taking place in the APS remain challenging to investigate *in vivo* and this is especially true in a human context. Using spatially confined hESCs, we uncovered signalling cross-talk and dynamics that correlate with key lineage restrictions in the APS and identified *in vitro* conditions for efficient derivation of NotoPs (Fig. 8).

Our first experiment showed that cells on micropatterns treated with 2 µM CHIR and 20 ng/ml FGF2 formed a domain of endodermal differentiation (Fig. 1B). This was at first surprising to us given that the same treatment in 2D monolayer cultures led to homogeneous NMP-like differentiation (Fig. 1B). This raised the question of what makes the cells on micropatterns adopt a distinct fate to that adopted in monolayer cultures.

Previous work using BMP and WNT ligands rather than CHIR revealed that epithelial integrity is compromised at the colony boundary, leading peripheral cells to be more responsive to signalling molecules than cells at the centre (Etoc et al., 2016; Legier et al., 2023; Martyn et al., 2018, 2019; Warmflash et al., 2014). Our data indicate that a similar boundary-driven mechanism is likely taking place here as well given that cell fate patterning did not scale with colony sizes (Fig. S2). CHIR is a cell-permeable molecule that bypasses ligand/receptor interactions and is therefore not affected by epithelial integrity (Fig. 5A). Nevertheless, our data support the idea that differential responsiveness to secondary signals downstream of CHIR exist in the colony. An inward wave of *NODAL* expression became apparent at 24 h after low levels of *NODAL* was initiated throughout the colony at earlier time points (Fig. 5D). Such a wave, also described in other systems (Heemskerk et al., 2019; Liu et al., 2022; Martyn et al., 2019), likely requires a boundary to be initiated and sufficient cell packing whereby NODAL signalling spreads from neighbour to neighbour via a relay mechanism (Liu et al., 2022). This mechanism may explain the difference in cell fate outcomes between micropatterns and monolayer cultures.

Importantly, existence of the NODAL wave means that the cells are subjected to distinct timing and duration of NODAL depending on their position along the colony radius (Fig. 8A). Early and sustained production of *NODAL* at the periphery (Fig. 5D) defined a domain of endodermal differentiation (Figs 1B, 2B-H and 4). Our data agree with recent work showing that WNT3A and FGF8 induce a similar spatiotemporal pattern of NODAL expression on micropatterns (figure 5J in Ortiz-Salazar et al., 2024 preprint). Both systems ultimately generate reproducible and self-organised 3D structures composed of endoderm with a core of SOX2^+^ cells as shown in Fig. 1F. These micropattern systems provide a powerful platform – complementary to other 3D human *in vitro* models (Moris et al., 2020; Vianello and Lutolf, 2020 preprint) – to further understand endoderm specification and morphogenesis in a human context. For example, although SOX17^+^ cells emerged preferentially in a domain overlapping the 24 h NODAL-expressing domain, they did so in a scattered manner at 36 h before regrouping together at the periphery (Fig. 2B,E). This apparent stochastic emergence followed by a sorting behaviour is reminiscent of observations in mouse ESC colonies (Pour et al., 2022), human gastruloids (Moris et al., 2020; Vianello and Lutolf, 2020 preprint), and mouse (Burtscher and Lickert, 2009; Scheibner et al., 2021) and pig (Simpson et al., 2024) embryos. Micropatterned colonies will be helpful to determine the mechanisms that ensure the robust outcome of this process.

Interestingly, we found that endoderm only emerges on micropatterns in a very small CHIR concentration range around 2 µM. Our CHIR dose-response experiment (Fig. 4) was particularly revealing and helped us to delineate the signalling sequences segregating individual APS cell fates from one another (summarised in Fig. 8). We show that even small variations in CHIR concentrations radically changed the balance of endodermal and mesodermal fates (Figs 4 and 8) and that this effect was mediated by a non-linear relationship between CHIR concentration and the downstream dynamics of NODAL expression (Figs 5C-E and 8). Interestingly, Ortiz and colleagues showed that a tenfold increase in WNT3A ligand concentration does not alter endoderm emergence (Ortiz-Salazar et al., 2024 preprint). Our complementary data identify the CHIR concentration range that relates to the physiological, ligand-based activation of the pathway and has important implications for the numerous studies using CHIR as a means to direct pluripotent stem cells differentiation.

CHIR induced LPM and ExM markers (Figs 2A, 4C and Fig. S6C), most likely under the influence of endogenous BMP signalling (Fig. S8) (Camacho-Aguilar et al., 2024; Heemskerk et al., 2019). Fate-mapping experiments have shown that cardiac mesoderm arises directly adjacent and posterior to the definitive endoderm (Lawson et al., 1991; Tam et al., 1997) and lineage tracing using FOXA2-cre mouse lines has demonstrated that a proportion of FOXA2-expressing cells in the streak are fated to form cardiac ventricles (Bardot et al., 2017). It will be interesting in the future to confirm the lineage hierarchy and identity of the HAND1^+^ cells emerging in our system. Remarkably, we also noted the presence of FOXA2-only cells emerging at 24 h around the central region of the colony (Fig. 2G). While their identity is unclear, these cells may form DE or hypoblast-like cells in the colonies based on our observations that hypoblast markers arise in our cultures (Fig. 2A, Fig. S4C). This would align with recent reports in mouse and pig embryos showing that FOXA2-only cells emerging anterior to the FOXA2^+^TBXT^+^ domain contribute to the DE/hypoblast layer (Scheibner et al., 2021; Simpson et al., 2024).

Most importantly, our data shed new light on how distinct signalling regimes are established and associated with individual cell fates (Fig. 8B,C): (1) We found that NM specification requires an environment in which NODAL activity is maintained at a minimum (Figs 3 and 8), in agreement with recent work showing that the maintenance of NMPs *in vitro* requires NODAL inhibition (Kelle et al., 2024 preprint). This result is also compatible with *in vivo* work showing that NM-fated cells appear later than endoderm, when NODAL signalling activity starts to decrease (Lawson et al., 1991). Furthermore, neighbouring NotoPs – as a source of NODAL inhibitors – may protect NMPs from advert differentiation during axial elongation and a release of NODAL inhibition might explain why NotoP ablation results in early termination of axial elongation (Abdelkhalek et al., 2004; McLaren and Steventon, 2021; Saunders et al., 2024 preprint; Wymeersch et al., 2019). (2) Our manipulations of NODAL and BMP signalling allowed us to identify the signalling that defines the notochord lineage (Figs 6A and 8B) and unveiled a signalling cross-talk whereby NODAL signalling dampens WNT activity (Fig. 7). We propose a model in which initial WNT and NODAL signalling combinatorially specify epiblast cells to an early FOXA2^+^TBXT^+^ APS state that is competent to form both endoderm and notochord. Whereas prolonged NODAL exposure keeps WNT activity low and drives endoderm differentiation, a sharp inhibition of NODAL signalling potentiates WNT activity and directs the cells to the notochord fate (Fig. 8B). Our model is consistent with observations in *Xenopus* whereby a sudden drop of p-Smad2 correlates with notochord emergence (Schohl and Fagotto, 2002) and with recent observations in pig embryos (Simpson et al., 2024). It will be important to test our model in an *in vivo* setting and determine the mechanisms that are evolutionarily conserved.

Reassuringly, our data are reproducible across multiple cell lines (Figs S7 and S9) and are consistent with a recent study reporting a similar requirement for delayed TGFβ signalling inhibition for the derivation of NotoPs on micropatterns (Rito et al., 2023 preprint). These authors also report cross-talk between hippo signalling and FGF activity in a domain overlapping with the domain of NotoP emergence. Our data showing that TGFβ inhibition potentiates WNT activity in this domain complement these findings. It will be important in the future to elucidate how the specific mechanical and biochemical environment defined at the colony boundaries *in vitro* reflects the *in vivo* environment of NotoPs in the embryo.

### Conclusion

NotoPs are regarded as a promising cell type for clinical applications and much remains to be learned about the healthy and pathological development of the notochord. Encouragingly, NotoPs persist as a small, transcriptionally stable population throughout axial elongation (Wymeersch et al., 2019), making it likely that NotoPs may be expandable in culture if the correct conditions can be identified. Here, we show that NotoPs can be efficiently generated from hESCs in the absence of genetic manipulation, via biochemical and mechanical control of the microenvironment. Our work, together with the recent work of others (Rito et al., 2023 preprint), will unlock new avenues for the study of the notochord lineage in human.

## MATERIALS AND METHODS

### Cell culture

Experiments were conducted with the MasterShef7 hESC line obtained from the University of Sheffield, UK, the RC17 hESC line from the University of Edinburgh, UK (De Sousa et al., 2016), the NAS2 hiPSC line from the Kunath lab (Devine et al., 2011) and the RUES-UFO triple reporter line derived in the Brivanlou lab (Martyn et al., 2018) from Benoit Sorre (University of Paris and Sorbonne University, Paris, France). Approval for the use of hESCs was granted by the Steering Committee for the UK Stem Cell Bank and for the Use of Stem Cell Lines. Cell lines were propagated at 37°C and 5% CO_2_ in mTSER Plus medium (100-0276, STEMCELL Technologies) on Geltrex (A1413302, Life Technologies)-coated 6-well plates (3516, Corning Incorporated). Wells were coated for 30 min at 37°C using a 100 µg/ml Geltrex solution diluted in magnesium- and calcium-containing DPBS (14080-048, Gibco; hereafter DPBS++). Passaging was performed every 2-3 days using Accutase (00-4555-56, Thermo Fisher Scientific) for all cell lines except for RUES-UFO cells for which ReLeSR (STEMCELL Technologies) was used instead. The cells were *Mycoplasma* tested prior to running experiments. Only cells with a passage number below 30 were used for all experiments.

### Micropattern fabrication

Micropatterning was carried out in ibidi 8-well µSlides (IB-80826, ibidi) with a PRIMO bioengineering platform (Alveole) mounted on a Nikon Ti2 widefield microscope using a protocol adapted from Alveole. The surface of ibidi slides was first passivated as follows: slides were plasma treated (Harrick Plasma Cleaner) for 1 min 30 s at high intensity in a 200 mTorr vacuum and then incubated at room temperature for 1 h with 200 µl/well of 200 µg/ml poly-D-lysine (A-003-E, Merck Millipore) diluted in 0.1 M HEPES pH 8.4 (H3784-25G, Sigma-Aldrich). The wells were washed twice with ddH_2_O and once with 0.1 M HEPES pH 8.4 and then incubated for 1 h in the dark with 125 µl/well of 80 mg/ml mPEG-SVA (MPEG-SVA-5000, Laysan Bio) freshly dissolved in 0.1 M HEPES pH 8.4. Slides were then washed profusely with ddH_2_O, air-dried and stored at 4°C until further processing (1 week maximum). The PRIMO insolation step was next performed less than 1 day prior to plating the cells: passivated wells were covered with 8 µl PLPP gel (Cairn Research; 1 µl PLPP gel/well diluted with 7 µl 70% ethanol) in the dark and left to dry for ∼30 min at room temperature. Slides were then insolated with PRIMO through a 20× lens with a dose of 50 mJ/cm^2^. All micropattern shapes were designed in Inkscape and converted to binary tiff files in ImageJ prior to loading in the Alveole Leonardo software. After insolation, PLPP gel was removed with three ddH2O washes and the slides were air-dried and stored at 4°C until use.

### Culture on micropatterns

Micropatterned ibidi slides were first rehydrated for 5 min in DPBS++. Matrix coating was then performed by incubating the wells at room temperature for 30 min on a rocker with a mixture of 40 µg/ml rhVitronectin-N (A14700, Thermo Fisher Scientific) and 10 µg/ml rhLaminin521 (A29249, Gibco) diluted in DPBS++. Wells were washed three times with DPBS++ and left in the last wash whilst preparing the cells for seeding to ensure that the wells were not left to dry. For seeding, 80% confluent cells were dissociated to single cells with Accutase, and resuspended in seeding medium composed of mTESR Plus supplemented with 10 µM Y-27632 (1254, Tocris Bio-Techne) and 1:100 penicillin/streptomycin (10,000 U/ml penicillin, 10,000 mg/ml streptomycin; 15140-122, Invitrogen). Cells were plated onto micropatterns at a density of 150,000 cells/well in 250 µl of seeding medium. The cells were left to adhere for 3 h at 37°C. After attachment, excess cells were removed by gentle pipetting and applying fresh seeding medium. The cells were left to settle and cover the patterns overnight until induction of differentiation the next morning. Cells were washed once in N2B27 to remove traces of growth factors present in seeding medium. Differentiation was then induced using N2B27 medium supplemented with penicillin/streptomycin (1:100), CHIR99021 (4423/10, Tocris Bio-Techne) at the concentrations indicated in the figure legends and 20 ng/ml human basic fibroblast growth factor (PHG6015, Thermo Fisher Scientific). SB431542 (1614, Tocris Bio-Techne) was used at 10 µM unless specified otherwise and LDN 193189 (72147, STEMCELL Technologies) at 0.1 µM.

### Immunofluorescence

Mouse embryos were staged according to Downs and Davies (1993) and stained as described previously by Wong (2021). Cells grown on micropatterns were fixed with 4% paraformaldehyde (CHE2036, Scientific Laboratory Supplies), washed three times with a solution of PBS and 0.1% Triton X-100 (A16046, Alfa Aesar; hereafter PBST) and left overnight at 4°C in blocking solution containing 3% donkey serum (D9663, Merck) and 0.03% sodium azide (40-2000-01, Severn Biotech Ltd.) in PBST. All primary antibodies (Table S2) were incubated overnight at 4°C and secondary antibodies (Table S3) at room temperature for 3 h. All washing steps were performed in PBST. Some co-staining required the use of primary antibodies raised in the same species. In these cases, the staining was performed with either pre-conjugated antibodies only, or sequentially using a non-conjugated antibody first, its corresponding secondary antibody next, followed by a blocking step using species-specific serum [3% goat serum (G9023-10ML, Sigma-Aldrich) or 3% rabbit serum (R9133, Merck)] and finally applying the conjugated antibody. Slide were washed three times in PBST and kept sealed at 4°C until imaging. For thick specimens, such as the elongated colonies in Fig. 3C, the samples were immersed in 1% low melting agarose and RapiClear 1.47 (RC147001, SunJin Lab).

### FISH

Branched DNA FISH was performed using the viewRNA Cell Plus assay from Thermo Fisher Scientific (88-19000-99) according to the manufacturer's protocol. FISH probes used in this study are listed in Table S4. Slides were imaged on the same day with a Nikon TiE widefield inverted microscope and a dry 20× lens.

### Immunofluorescence imaging and image analysis

Embryos were imaged in PBST using a Leica TCS SP8 confocal microscope with a 25× water immersion lens. All images were annotated and contrast-adjusted using Fiji (Schindelin et al., 2012). Micropatterned colonies were imaged with an Opera Phenix Plus imaging system (Perkin Elmer). ibidi slides contained around 64 colonies per well, out of which ∼20 colonies were selected for analysis in each experiment. To ensure an unbiased sampling of the colonies, the slides were first fully scanned with a 10× lens to generate overview images of the LMBR (nuclear envelope marker) signal only. These images were then processed with an automated pipeline in Harmony software (Perkin Elmer). This pipeline rejected colonies with an unexpected area or roundness and then randomly sampled 20 colonies from the pool of valid colonies. Sampled colonies were next imaged with a 20× lens to generate 3D multichannel *z*-stacks with voxel size of 0.59×0.59×1 µm. Opera images were then exported as Tiff files for further analysis. Nuclear segmentation was performed on the LMBR signal as described previously (Blin et al., 2019). Raw images and nuclear masks were imported into PickCells (https://pickcellslab.frama.io/docs/) to compute nuclear features, including 3D spatial coordinates and average intensities in all fluorescence channels. The tsv file created in PickCells was then analysed in Python. Our Jupyter Notebooks and data files are available in our Gitlab repository (https://framagit.org/pickcellslab/data/2023_haxioms).

### FISH imaging and image analysis

Micropatterned colonies were imaged on an inverted widefield microscope (Nikon Eclipse Ti) with a long distance 10× lens to image entire wells (8 mm×8 mm). Images were then segmented using Cellpose 2.0 (Stringer et al., 2021) to identify individual colonies. We used the live-cell model as a starting point and manually adjusted the model according to the Cellpose tutorial using at least five different images to ensure accurate segmentation across our conditions. Images were scaled down for the segmentation process to save on computational resources and scaled back up to their original sizes using ImageJ prior to loading into PickCells for further analysis. PickCells was used to compute average intensities in each channel within each colony, as well as background intensity in the immediate vicinity of the colony (bounding box intensity – colony intensity) and radial intensities as described in Fig. 5. Data were next analysed in Python. The colonies were selected based on their compactness and surface area, to avoid including abnormally sized and shaped colonies (e.g. partially broken colonies). The background intensity was subtracted from the average and radial colony intensities and plotted in Python.

### NanoString analysis

RNA samples were prepared using an Absolutely RNA microprep kit (400805, Agilent Technologies) and NanoString profiling was performed using nCounter technology as per the manufacturer's instructions. We used a panel of probes consisting of the 780 genes included in the standard human embryonic stem cell gene panel together with 30 additional custom probes (all genes are listed in Table S1). Normalisation of raw data was accomplished in the NanoString dedicated nCounter software. Next, raw counts were imported in R (R Core Team, 2013) and analysed with the Bioconductor package moanin (Varoquaux and Purdom, 2020). We first applied an initial cut-off to filter out all the genes for which the max count was below 100. The data was then log2 transformed. We kept only the top 50% most variable genes based on the median absolute deviation (mad) metric over time. A spline was then fitted onto each individual gene profile and we grouped genes into seven clusters using kmeans clustering on the parameters of the fitted splines to obtain the heatmaps shown in Fig. 2A. R scripts and data are available in our Gitlab repository (https://framagit.org/pickcellslab/data/2023_haxioms).

### Mouse husbandry

Mouse work was carried out under the UK Home Office project license PPL PEEC9E359, approved by the University of Edinburgh Animal Welfare and Ethical Review Panel and in compliance with the Animals (Scientific Procedures) Act 1986. Mice used were of C57Bl6/J strain background. The mice were kept under a 12 h light/dark cycle, and the embryo age was denoted day 0 on the midpoint of the dark cycle the day the plug was found.

## Supplementary Material



10.1242/develop.202983_sup1 Supplementary information

Table S1. List of NanoString probes

## References

[DEV202983C1] Abdelkhalek, H. B., Beckers, A., Schuster-Gossler, K., Pavlova, M. N., Burkhardt, H., Lickert, H., Rossant, J., Reinhardt, R., Schalkwyk, L. C., Müller, I. et al. (2004). The mouse homeobox gene Not is required for caudal notochord development and affected by the truncate mutation. *Genes Dev.* 18, 1725-1736. 10.1101/gad.303504 15231714 PMC478193

[DEV202983C2] Aksoy, I., Giudice, V., Delahaye, E., Wianny, F., Aubry, M., Mure, M., Chen, J., Jauch, R., Bogu, G. K., Nolden, T. et al. (2014). Klf4 and Klf5 differentially inhibit mesoderm and endoderm differentiation in embryonic stem cells. *Nat. Commun.* 5, 3719. 10.1038/ncomms4719 24770696

[DEV202983C3] Albors, A. R., Halley, P. A. and Storey, K. G. (2018). Lineage tracing of axial progenitors using Nkx1-2CreERT2 mice defines their trunk and tail contributions. *Development* 145, dev164319. 10.1242/dev.164319 30201686 PMC6198475

[DEV202983C4] Ang, S. L. and Rossant, J. (1994). HNF-3β is essential for node and notochord formation in mouse development. *Cell* 78, 561-574. 10.1016/0092-8674(94)90522-3 8069909

[DEV202983C5] Arnold, S. J., Stappert, J., Bauer, A., Kispert, A., Herrmann, B. G. and Kemler, R. (2000). Brachyury is a target gene of the Wnt/beta-catenin signaling pathway. *Mech. Dev.* 91, 249-258. 10.1016/S0925-4773(99)00309-3 10704849

[DEV202983C6] Avilion, A. A., Nicolis, S. K., Pevny, L. H., Perez, L., Vivian, N. and Lovell-Badge, R. (2003). Multipotent cell lineages in early mouse development depend on SOX2 function. *Genes Dev.* 17, 126-140. 10.1101/gad.224503 12514105 PMC195970

[DEV202983C7] Aykul, S., Ni, W., Mutatu, W. and Martinez-Hackert, E. (2015). Human cerberus prevents nodal-receptor binding, inhibits nodal signaling, and suppresses nodal-mediated phenotypes. *PLoS ONE* 10, e0114954. 10.1371/journal.pone.0114954 25603319 PMC4300205

[DEV202983C8] Bachiller, D., Klingensmith, J., Kemp, C., Belo, J. A., Anderson, R. M., May, S. R., McMahon, J. A., McMahon, A. P., Harland, R. M., Rossant, J. et al. (2000). The organizer factors chordin and noggin are required for mouse forebrain development. *Nature* 403, 658-661. 10.1038/35001072 10688202

[DEV202983C9] Bagheri-Fam, S., Barrionuevo, F., Dohrmann, U., Günther, T., Schüle, R., Kemler, R., Mallo, M., Kanzler, B. and Scherer, G. (2006). Long-range upstream and downstream enhancers control distinct subsets of the complex spatiotemporal Sox9 expression pattern. *Dev. Biol.* 291, 382-397. 10.1016/j.ydbio.2005.11.013 16458883

[DEV202983C10] Bagnat, M. and Gray, R. S. (2020). Development of a straight vertebrate body axis. *Development* 147, dev175794. 10.1242/dev.175794 33023886 PMC7561478

[DEV202983C11] Balmer, S., Nowotschin, S. and Hadjantonakis, A.-K. (2016). Notochord morphogenesis in mice: current understanding & open questions. *Dev. Dyn.* 245, 547-557. 10.1002/dvdy.24392 26845388 PMC4844759

[DEV202983C12] Bardot, E., Calderon, D., Santoriello, F., Han, S., Cheung, K., Jadhav, B., Burtscher, I., Artap, S., Jain, R., Epstein, J. et al. (2017). Foxa2 identifies a cardiac progenitor population with ventricular differentiation potential. *Nat. Commun.* 8, 14428. 10.1038/ncomms14428 28195173 PMC5316866

[DEV202983C13] Barnes, R. M., Firulli, B. A., Conway, S. J., Vincentz, J. W. and Firulli, A. B. (2010). Analysis of the Hand1 cell lineage reveals novel contributions to cardiovascular, neural crest, extra-embryonic, and lateral mesoderm derivatives. *Dev. Dyn.* 239, 3086-3097. 10.1002/dvdy.22428 20882677 PMC2965316

[DEV202983C14] Beccari, L., Moris, N., Girgin, M., Turner, D. A., Baillie-Johnson, P., Cossy, A.-C., Lutolf, M. P., Duboule, D. and Arias, A. M. (2018). Multi-axial self-organization properties of mouse embryonic stem cells into gastruloids. *Nature* 562, 272-276. 10.1038/s41586-018-0578-0 30283134

[DEV202983C15] Beddington, R. S. and Robertson, E. J. (1989). An assessment of the developmental potential of embryonic stem cells in the midgestation mouse embryo. *Development* 105, 733-737. 10.1242/dev.105.4.733 2598811

[DEV202983C16] Bergmann, S., Penfold, C. A., Slatery, E., Siriwardena, D., Drummer, C., Clark, S., Strawbridge, S. E., Kishimoto, K., Vickers, A., Tewary, M. et al. (2022). Spatial profiling of early primate gastrulation in utero. *Nature* 609, 136-143. 10.1038/s41586-022-04953-1 35709828 PMC7614364

[DEV202983C17] Blin, G. (2021). Quantitative developmental biology in vitro using micropatterning. *Development* 148, dev186387. 10.1242/dev.186387 34345912 PMC8353268

[DEV202983C18] Blin, G., Sadurska, D., Migueles, R. P., Chen, N., Watson, J. A. and Lowell, S. (2019). Nessys: a new set of tools for the automated detection of nuclei within intact tissues and dense 3D cultures. *PLoS Biol.* 17, e3000388. 10.1371/journal.pbio.3000388 31398189 PMC6703695

[DEV202983C19] Bohn, S., Hexemer, L., Huang, Z., Strohmaier, L., Lenhardt, S., Legewie, S. and Loewer, A. (2023). State- and stimulus-specific dynamics of SMAD signaling determine fate decisions in individual cells. *Proc. Natl Acad. Sci. USA* 120, e2210891120. 10.1073/pnas.2210891120 36857347 PMC10013741

[DEV202983C20] Burtscher, I. and Lickert, H. (2009). Foxa2 regulates polarity and epithelialization in the endoderm germ layer of the mouse embryo. *Development* 136, 1029-1038. 10.1242/dev.028415 19234065

[DEV202983C21] Cadigan, K. M. and Waterman, M. L. (2012). TCF/LEFs and Wnt signaling in the nucleus. *Cold Spring Harb. Perspect. Biol.* 4, a007906. 10.1101/cshperspect.a007906 23024173 PMC3536346

[DEV202983C22] Camacho-Aguilar, E., Yoon, S. T., Ortiz-Salazar, M. A., Du, S., Guerra, M. C. and Warmflash, A. (2024). Combinatorial interpretation of BMP and WNT controls the decision between primitive streak and extraembryonic fates. *Cell Syst.* 15, 445-461.e4. 10.1016/j.cels.2024.04.001 38692274 PMC11231731

[DEV202983C23] Cermola, F., D'Aniello, C., Tatè, R., Cesare, D. D., Martinez-Arias, A., Minchiotti, G. and Patriarca, E. J. (2021). Gastruloid development competence discriminates different states of pluripotency. *Stem Cell Rep.* 16, 354-369. 10.1016/j.stemcr.2020.12.013 PMC7878839 33482102

[DEV202983C24] Chhabra, S., Liu, L., Goh, R., Kong, X. and Warmflash, A. (2019). Dissecting the dynamics of signaling events in the BMP, WNT, and NODAL cascade during self-organized fate patterning in human gastruloids. *PLoS Biol.* 17, e3000498. 10.1371/journal.pbio.3000498 31613879 PMC6814242

[DEV202983C25] Choi, K.-S., Cohn, M. J. and Harfe, B. D. (2008). Identification of nucleus pulposus precursor cells and notochordal remnants in the mouse: implications for disk degeneration and chordoma formation. *Dev. Dyn.* 237, 3953-3958. 10.1002/dvdy.21805 19035356 PMC2646501

[DEV202983C26] Cohen, P. and Goedert, M. (2004). GSK3 inhibitors: development and therapeutic potential. *Nat. Rev. Drug Discov.* 3, 479-487. 10.1038/nrd1415 15173837

[DEV202983C27] Colombier, P., Halgand, B., Chédeville, C., Chariau, C., François-Campion, V., Kilens, S., Vedrenne, N., Clouet, J., David, L., Guicheux, J. et al. (2020). NOTO transcription factor directs human induced pluripotent stem cell-derived mesendoderm progenitors to a notochordal fate. *Cells* 9, 509. 10.3390/cells9020509 32102328 PMC7072849

[DEV202983C28] Costello, I., Pimeisl, I.-M., Dräger, S., Bikoff, E. K., Robertson, E. J. and Arnold, S. J. (2011). The T-box transcription factor Eomesodermin acts upstream of Mesp1 to specify cardiac mesoderm during mouse gastrulation. *Nat. Cell Biol.* 13, 1084-1091. 10.1038/ncb2304 21822279 PMC4531310

[DEV202983C29] Costello, I., Nowotschin, S., Sun, X., Mould, A. W., Hadjantonakis, A.-K., Bikoff, E. K. and Robertson, E. J. (2015). Lhx1 functions together with Otx2, Foxa2, and Ldb1 to govern anterior mesendoderm, node, and midline development. *Genes Dev.* 29, 2108-2122. 10.1101/gad.268979.115 26494787 PMC4617976

[DEV202983C30] De Bakker, B. S., De Jong, K. H., Hagoort, J., De Bree, K., Besselink, C. T., De Kanter, F. E. C., Veldhuis, T., Bais, B., Schildmeijer, R., Ruijter, J. M. et al. (2016). An interactive three-dimensional digital atlas and quantitative database of human development. *Science* 354, aag0053. 10.1126/science.aag0053 27884980

[DEV202983C31] De Bree, K., De Bakker, B. S. and Oostra, R.-J. (2018). The development of the human notochord. *PLoS ONE* 13, e0205752. 10.1371/journal.pone.0205752 30346967 PMC6197658

[DEV202983C32] De Sousa, P. A., Tye, B. J., Bruce, K., Dand, P., Russell, G., Collins, D. M., Greenshields, A., McDonald, K., Bradburn, H., Allan, D. et al. (2016). Derivation of the clinical grade human embryonic stem cell line RCe021-A (RC-17). *Stem Cell Res.* 17, 1-5. 10.1016/j.scr.2016.04.019 27558596

[DEV202983C33] Devine, M. J., Ryten, M., Vodicka, P., Thomson, A. J., Burdon, T., Houlden, H., Cavaleri, F., Nagano, M., Drummond, N. J., Taanman, J.-W. et al. (2011). Parkinson's disease induced pluripotent stem cells with triplication of the α-synuclein locus. *Nat. Commun.* 2, 440. 10.1038/ncomms1453 21863007 PMC3265381

[DEV202983C34] Diaz-Hernandez, M. E., Khan, N. M., Trochez, C. M., Yoon, T., Maye, P., Presciutti, S. M., Gibson, G. and Drissi, H. (2020). Derivation of notochordal cells from human embryonic stem cells reveals unique regulatory networks by single cell-transcriptomics. *J. Cell. Physiol.* 235, 5241-5255. 10.1002/jcp.29411 31840817 PMC7056550

[DEV202983C117] Downs, K. M. and Davies, T. (1993). Staging of gastrulating mouse embryos by morphological landmarks in the dissecting microscope. *Development* 118, 1255-1266. 10.1242/dev.118.4.1255 8269852

[DEV202983C35] Dunn, N. R., Vincent, S. D., Oxburgh, L., Robertson, E. J. and Bikoff, E. K. (2004). Combinatorial activities of Smad2 and Smad3 regulate mesoderm formation and patterning in the mouse embryo. *Development* 131, 1717-1728. 10.1242/dev.01072 15084457

[DEV202983C36] Edri, S., Hayward, P., Jawaid, W. and Arias, A. M. (2019). Neuro-mesodermal progenitors (NMPs): a comparative study between pluripotent stem cells and embryo-derived populations. *Development* 146, dev180190. 10.1242/dev.180190 31152001 PMC6602346

[DEV202983C37] Etoc, F., Metzger, J., Ruzo, A., Kirst, C., Yoney, A., Ozair, M. Z., Brivanlou, A. H. and Siggia, E. D. (2016). A balance between secreted inhibitors and edge sensing controls gastruloid self-organization. *Dev. Cell* 39, 302-315. 10.1016/j.devcel.2016.09.016 27746044 PMC5113147

[DEV202983C38] Forlani, S., Lawson, K. A. and Deschamps, J. (2003). Acquisition of Hox codes during gastrulation and axial elongation in the mouse embryo. *Development* 130, 3807-3819. 10.1242/dev.00573 12835396

[DEV202983C39] Frith, T. J. R. and Tsakiridis, A. (2019). Efficient generation of trunk neural crest and sympathetic neurons from human pluripotent stem cells via a neuromesodermal axial progenitor intermediate. *Curr. Protoc. Stem Cell Biol.* 49, e81. 10.1002/cpsc.81 30688409 PMC6520238

[DEV202983C40] Frith, T. J., Granata, I., Wind, M., Stout, E., Thompson, O., Neumann, K., Stavish, D., Heath, P. R., Ortmann, D., Hackland, J. O. et al. (2018). Human axial progenitors generate trunk neural crest cells in vitro. *eLife* 7, e35786. 10.7554/eLife.35786 30095409 PMC6101942

[DEV202983C41] Gouti, M., Tsakiridis, A., Wymeersch, F. J., Huang, Y., Kleinjung, J., Wilson, V. and Briscoe, J. (2014). In vitro generation of neuromesodermal progenitors reveals distinct roles for Wnt signalling in the specification of spinal cord and paraxial mesoderm identity. *PLoS Biol.* 12, e1001937. 10.1371/journal.pbio.1001937 25157815 PMC4144800

[DEV202983C42] Gunne-Braden, A., Sullivan, A., Gharibi, B., Sheriff, R. S. M., Maity, A., Wang, Y.-F., Edwards, A., Jiang, M., Howell, M., Goldstone, R. et al. (2020). GATA3 mediates a fast, irreversible commitment to BMP4-driven differentiation in human embryonic stem cells. *Cell Stem Cell* 26, 693-706.e9. 10.1016/j.stem.2020.03.005 32302522 PMC7487786

[DEV202983C43] Hart, A. H., Hartley, L., Sourris, K., Stadler, E. S., Li, R., Stanley, E. G., Tam, P. P. L., Elefanty, A. G. and Robb, L. (2002). Mixl1 is required for axial mesendoderm morphogenesis and patterning in the murine embryo. *Development* 129, 3597-3608. 10.1242/dev.129.15.3597 12117810

[DEV202983C44] Hart, A. H., Hartley, L., Ibrahim, M. and Robb, L. (2004). Identification, cloning and expression analysis of the pluripotency promoting Nanog genes in mouse and human. *Dev. Dyn.* 230, 187-198. 10.1002/dvdy.20034 15108323

[DEV202983C45] Heemskerk, I., Burt, K., Miller, M., Chhabra, S., Guerra, M. C., Liu, L. and Warmflash, A. (2019). Rapid changes in morphogen concentration control self-organized patterning in human embryonic stem cells. *eLife* 8, e40526. 10.7554/eLife.40526 30829572 PMC6398983

[DEV202983C46] Henrique, D., Abranches, E., Verrier, L. and Storey, K. G. (2015). Neuromesodermal progenitors and the making of the spinal cord. *Development* 142, 2864-2875. 10.1242/dev.119768 26329597 PMC4958456

[DEV202983C47] Inman, G. J., Nicolás, F. J., Callahan, J. F., Harling, J. D., Gaster, L. M., Reith, A. D., Laping, N. J. and Hill, C. S. (2002). SB-431542 is a potent and specific inhibitor of transforming growth factor-beta superfamily type I activin receptor-like kinase (ALK) receptors ALK4, ALK5, and ALK7. *Mol. Pharmacol.* 62, 65-74. 10.1124/mol.62.1.65 12065756

[DEV202983C48] Kanai-Azuma, M., Kanai, Y., Gad, J. M., Tajima, Y., Taya, C., Kurohmaru, M., Sanai, Y., Yonekawa, H., Yazaki, K., Tam, P. P. L. et al. (2002). Depletion of definitive gut endoderm in Sox17-null mutant mice. *Development* 129, 2367-2379. 10.1242/dev.129.10.2367 11973269

[DEV202983C49] Kelle, D., Ugur, E., Rusha, E., Shaposhnikov, D., Livigni, A., Horschitz, S., Davoudi, M., Blutke, A., Bushe, J., Sterr, M. et al. (2024). Capture of human neuromesodermal and posterior neural tube axial stem cells. *bioRxiv* 2024.03.26.586760. 10.1101/2024.03.26.586760

[DEV202983C50] Kinder, S. J., Tsang, T. E., Wakamiya, M., Sasaki, H., Behringer, R. R., Nagy, A. and Tam, P. P. L. (2001). The organizer of the mouse gastrula is composed of a dynamic population of progenitor cells for the axial mesoderm. *Development* 128, 3623-3634. 10.1242/dev.128.18.3623 11566865

[DEV202983C51] Kwon, G. S., Viotti, M. and Hadjantonakis, A.-K. (2008). The endoderm of the mouse embryo arises by dynamic widespread intercalation of embryonic and extraembryonic lineages. *Dev. Cell* 15, 509-520. 10.1016/j.devcel.2008.07.017 18854136 PMC2677989

[DEV202983C52] Lawson, K. A., Meneses, J. J. and Pedersen, R. A. (1991). Clonal analysis of epiblast fate during germ layer formation in the mouse embryo. *Development* 113, 891-911. 10.1242/dev.113.3.891 1821858

[DEV202983C53] Legier, T., Rattier, D., Llewellyn, J., Vannier, T., Sorre, B., Maina, F. and Dono, R. (2023). Epithelial disruption drives mesendoderm differentiation in human pluripotent stem cells by enabling TGF-β protein sensing. *Nat. Commun.* 14, 349. 10.1038/s41467-023-35965-8 36681697 PMC9867713

[DEV202983C54] Lescroart, F., Wang, X., Lin, X., Swedlund, B., Gargouri, S., Sànchez-Dànes, A., Moignard, V., Dubois, C., Paulissen, C., Kinston, S. et al. (2018). Defining the earliest step of cardiovascular lineage segregation by single-cell RNA-seq. *Science* 359, 1177-1181. 10.1126/science.aao4174 29371425 PMC6556615

[DEV202983C55] Lickert, H., Kutsch, S., Kanzler, B., Tamai, Y., Taketo, M. M. and Kemler, R. (2002). Formation of multiple hearts in mice following deletion of beta-catenin in the embryonic endoderm. *Dev. Cell* 3, 171-181. 10.1016/S1534-5807(02)00206-X 12194849

[DEV202983C56] Liu, L., Nemashkalo, A., Rezende, L., Jung, J. Y., Chhabra, S., Guerra, M. C., Heemskerk, I. and Warmflash, A. (2022). Nodal is a short-range morphogen with activity that spreads through a relay mechanism in human gastruloids. *Nat. Commun.* 13, 497. 10.1038/s41467-022-28149-3 35079017 PMC8789905

[DEV202983C57] Lolas, M., Valenzuela, P. D. T., Tjian, R. and Liu, Z. (2014). Charting Brachyury-mediated developmental pathways during early mouse embryogenesis. *Proc. Natl. Acad Sci. USA* 111, 4478-4483. 10.1073/pnas.1402612111 24616493 PMC3970479

[DEV202983C58] MacKinlay, K. M., Weatherbee, B. A., Souza Rosa, V., Handford, C. E., Hudson, G., Coorens, T., Pereira, L. V., Behjati, S., Vallier, L., Shahbazi, M. N. et al. (2021). An in vitro stem cell model of human epiblast and yolk sac interaction. *eLife* 10, e63930. 10.7554/eLife.63930 34403333 PMC8370770

[DEV202983C59] Martins, J.-M. F., Fischer, C., Urzi, A., Vidal, R., Kunz, S., Ruffault, P.-L., Kabuss, L., Hube, I., Gazzerro, E., Birchmeier, C. et al. (2020). Self-organizing 3D human trunk neuromuscular organoids. *Cell Stem Cell* 26, 172-186.e6. 10.1016/j.stem.2019.12.007 31956040

[DEV202983C60] Martins-Costa, C., Wilson, V. and Binagui-Casas, A. (2024). Chapter six - neuromesodermal specification during head-to-tail body axis formation. In: *Current Topics in Developmental Biology* (ed. M. Mallo), pp. 232-271. Academic Press. 10.1016/bs.ctdb.2024.02.012 38729677

[DEV202983C61] Martyn, I., Kanno, T. Y., Ruzo, A., Siggia, E. D. and Brivanlou, A. H. (2018). Self-organization of a human organizer by combined Wnt and Nodal signalling. *Nature* 558, 132-135. 10.1038/s41586-018-0150-y 29795348 PMC6077985

[DEV202983C62] Martyn, I., Brivanlou, A. H. and Siggia, E. D. (2019). A wave of WNT signaling balanced by secreted inhibitors controls primitive streak formation in micropattern colonies of human embryonic stem cells. *Development* 146, dev172791. 10.1242/dev.172791 30814117 PMC6451321

[DEV202983C63] McCann, M. R., Tamplin, O. J., Rossant, J. and Séguin, C. A. (2012). Tracing notochord-derived cells using a Noto-cre mouse: implications for intervertebral disc development. *Dis. Model. Mech.* 5, 73-82. 10.1242/dmm.008128 22028328 PMC3255545

[DEV202983C64] McLaren, S. B. P. and Steventon, B. J. (2021). Anterior expansion and posterior addition to the notochord mechanically coordinate zebrafish embryo axis elongation. *Development* 148, dev199459. 10.1242/dev.199459 34086031 PMC8327291

[DEV202983C65] Merrill, B. J., Pasolli, H. A., Polak, L., Rendl, M., García-García, M. J., Anderson, K. V. and Fuchs, E. (2004). Tcf3: a transcriptional regulator of axis induction in the early embryo. *Development* 131, 263-274. 10.1242/dev.00935 14668413

[DEV202983C66] Moris, N., Anlas, K., Van Den Brink, S. C., Alemany, A., Schröder, J., Ghimire, S., Balayo, T., Van Oudenaarden, A. and Martinez Arias, A. (2020). An in vitro model of early anteroposterior organization during human development. *Nature* 582, 410-415. 10.1038/s41586-020-2383-9 32528178

[DEV202983C67] Mulas, C., Chia, G., Jones, K. A., Hodgson, A. C., Stirparo, G. G. and Nichols, J. (2018). Oct4 regulates the embryonic axis and coordinates exit from pluripotency and germ layer specification in the mouse embryo. *Development* 145, dev159103. 10.1242/dev.159103 29915126 PMC6031404

[DEV202983C68] Neijts, R., Simmini, S., Giuliani, F., Van Rooijen, C. and Deschamps, J. (2014). Region-specific regulation of posterior axial elongation during vertebrate embryogenesis. *Dev. Dyn.* 243, 88-98. 10.1002/dvdy.24027 23913366

[DEV202983C69] Oldak, B., Wildschutz, E., Bondarenko, V., Comar, M.-Y., Zhao, C., Aguilera-Castrejon, A., Tarazi, S., Viukov, S., Pham, T. X. A., Ashouokhi, S. et al. (2023). Complete human day 14 post-implantation embryo models from naive ES cells. *Nature* 622, 562-573. 10.1038/s41586-023-06604-5 37673118 PMC10584686

[DEV202983C70] Olmsted, Z. T. and Paluh, J. L. (2021). Co-development of central and peripheral neurons with trunk mesendoderm in human elongating multi-lineage organized gastruloids. *Nat. Commun.* 12, 3020. 10.1038/s41467-021-23294-7 34021144 PMC8140076

[DEV202983C71] Ortiz-Salazar, M. A., Camacho-Aguilar, E. and Warmflash, A. (2024). Endogenous Nodal switches Wnt interpretation from posteriorization to germ layer differentiation in geometrically constrained human pluripotent cells. *bioRxiv* 2024.03.13.584912. 10.1101/2024.03.13.584912

[DEV202983C72] Osorno, R., Tsakiridis, A., Wong, F., Cambray, N., Economou, C., Wilkie, R., Blin, G., Scotting, P. J., Chambers, I. and Wilson, V. (2012). The developmental dismantling of pluripotency is reversed by ectopic Oct4 expression. *Development* 139, 2288-2298. 10.1242/dev.078071 22669820 PMC3367440

[DEV202983C73] Paillat, L., Coutant, K., Dutilleul, M., Le Lay, S. and Camus, A. (2023). Three-dimensional culture model to study the biology of vacuolated notochordal cells from mouse nucleus pulposus explants. *Eur. Cell Mater.* 45, 72-87. 10.22203/eCM.v045a06 36866514

[DEV202983C74] Pham, T. X. A., Panda, A., Kagawa, H., To, S. K., Ertekin, C., Georgolopoulos, G., Van Knippenberg, S. S. F. A., Allsop, R. N., Bruneau, A., Chui, J. S.-H. et al. (2022). Modeling human extraembryonic mesoderm cells using naive pluripotent stem cells. *Cell Stem Cell* 29, 1346-1365.e10. 10.1016/j.stem.2022.08.001 36055191 PMC9438972

[DEV202983C75] Plouhinec, J.-L., Granier, C., Le Mentec, C., Lawson, K. A., Sabéran-Djoneidi, D., Aghion, J., Shi, D. L., Collignon, J. and Mazan, S. (2004). Identification of the mammalian Not gene via a phylogenomic approach. *Gene Expr. Patterns* 5, 11-22. 10.1016/j.modgep.2004.06.010 15533813

[DEV202983C76] Pour, M., Kumar, A. S., Farag, N., Bolondi, A., Kretzmer, H., Walther, M., Wittler, L., Meissner, A. and Nachman, I. (2022). Emergence and patterning dynamics of mouse-definitive endoderm. *iScience* 25, 103556. 10.1016/j.isci.2021.103556 34988400 PMC8693470

[DEV202983C77] Probst, S., Sagar, Tosic, J., Schwan, C., Grün, D. and Arnold, S. J. (2021). Spatiotemporal sequence of mesoderm and endoderm lineage segregation during mouse gastrulation. *Development* 148, dev193789. 10.1242/dev.193789 33199445

[DEV202983C78] R Core Team (2013). *R: A Language and Environment for Statistical Computing*. Vienna, Austria: R Foundation for Statistical Computing.

[DEV202983C79] Repina, N. A., Johnson, H. J., Bao, X., Zimmermann, J. A., Joy, D. A., Bi, S. Z., Kane, R. S. and Schaffer, D. V. (2023). Optogenetic control of Wnt signaling models cell-intrinsic embryogenic patterning using 2D human pluripotent stem cell culture. *Development* 150, dev201386. 10.1242/dev.201386 37401411 PMC10399980

[DEV202983C80] Rito, T., Libby, A. R. G., Demuth, M. and Briscoe, J. (2023). Notochord and axial progenitor generation by timely BMP and NODAL inhibition during vertebrate trunk formation. *bioRxiv* 2023.02.27.530267. 10.1101/2023.02.27.530267

[DEV202983C81] Robertson, E. J. (2014). Dose-dependent Nodal/Smad signals pattern the early mouse embryo. *Semin. Cell Dev. Biol.* 32, 73-79. 10.1016/j.semcdb.2014.03.028 24704361

[DEV202983C82] Saga, Y., Miyagawa-Tomita, S., Takagi, A., Kitajima, S., Miyazaki, J. I. and Inoue, T. (1999). MesP1 is expressed in the heart precursor cells and required for the formation of a single heart tube. *Development* 126, 3437-3447. 10.1242/dev.126.15.3437 10393122

[DEV202983C83] Sagy, N., Slovin, S., Allalouf, M., Pour, M., Savyon, G., Boxman, J. and Nachman, I. (2019). Prediction and control of symmetry breaking in embryoid bodies by environment and signal integration. *Development* 146, dev181917. 10.1242/dev.181917 31575644

[DEV202983C84] Sanaki-Matsumiya, M., Matsuda, M., Gritti, N., Nakaki, F., Sharpe, J., Trivedi, V. and Ebisuya, M. (2022). Periodic formation of epithelial somites from human pluripotent stem cells. *Nat. Commun.* 13, 2325. 10.1038/s41467-022-29967-1 35484123 PMC9050736

[DEV202983C85] Saunders, D., Camacho, C. and Steventon, B. (2024). Spinal cord elongation enables proportional regulation of the zebrafish posterior body. *bioRxiv* 2024.04.02.587732. 10.1101/2024.04.02.587732 PMC11829759 39745249

[DEV202983C86] Scheibner, K., Schirge, S., Burtscher, I., Büttner, M., Sterr, M., Yang, D., Böttcher, A., Ansarullah, Irmler, M., Beckers, J. et al. (2021). Epithelial cell plasticity drives endoderm formation during gastrulation. *Nat. Cell Biol.* 23, 692-703. 10.1038/s41556-021-00694-x 34168324 PMC8277579

[DEV202983C87] Schifferl, D., Scholze-Wittler, M., Luque, A. V., Pustet, M., Wittler, L., Veenvliet, J. V., Koch, F. and Herrmann, B. G. (2023). Genome-wide identification of notochord enhancers comprising the regulatory landscape of the Brachyury (T) locus in mouse. *Development* 150, dev202111. 10.1242/dev.202111 37882764 PMC10651091

[DEV202983C88] Schindelin, J., Arganda-Carreras, I., Frise, E., Kaynig, V., Longair, M., Pietzsch, T., Preibisch, S., Rueden, C., Saalfeld, S., Schmid, B. et al. (2012). Fiji: an open-source platform for biological-image analysis. *Nat. Methods* 9, 676-682. 10.1038/nmeth.2019 22743772 PMC3855844

[DEV202983C89] Schohl, A. and Fagotto, F. (2002). Beta-catenin, MAPK and Smad signaling during early Xenopus development. *Development* 129, 37-52. 10.1242/dev.129.1.37 11782399

[DEV202983C90] Simpson, L., Strange, A., Klisch, D., Kraunsoe, S., Azami, T., Goszczynski, D., Le Minh, T., Planells, B., Holmes, N., Sang, F. et al. (2024). A single-cell atlas of pig gastrulation as a resource for comparative embryology. *Nat. Commun.* 15, 5210. 10.1038/s41467-024-49407-6 38890321 PMC11189408

[DEV202983C91] Stemple, D. L. (2005). Structure and function of the notochord: an essential organ for chordate development. *Development* 132, 2503-2512. 10.1242/dev.01812 15890825

[DEV202983C92] Streit, A. and Stern, C. D. (1999). Mesoderm patterning and somite formation during node regression: differential effects of chordin and noggin. *Mech. Dev.* 85, 85-96. 10.1016/S0925-4773(99)00085-4 10415349

[DEV202983C93] Stringer, C., Wang, T., Michaelos, M. and Pachitariu, M. (2021). Cellpose: a generalist algorithm for cellular segmentation. *Nat. Methods* 18, 100-106. 10.1038/s41592-020-01018-x 33318659

[DEV202983C94] Tam, P. P., Parameswaran, M., Kinder, S. J. and Weinberger, R. P. (1997). The allocation of epiblast cells to the embryonic heart and other mesodermal lineages: the role of ingression and tissue movement during gastrulation. *Development* 124, 1631-1642. 10.1242/dev.124.9.1631 9165112

[DEV202983C95] Tamplin, O. J., Cox, B. J. and Rossant, J. (2011). Integrated microarray and ChIP analysis identifies multiple Foxa2 dependent target genes in the notochord. *Dev. Biol.* 360, 415-425. 10.1016/j.ydbio.2011.10.002 22008794

[DEV202983C96] Turner, D. A., Girgin, M., Alonso-Crisostomo, L., Trivedi, V., Baillie-Johnson, P., Glodowski, C. R., Hayward, P. C., Collignon, J., Gustavsen, C., Serup, P. et al. (2017). Anteroposterior polarity and elongation in the absence of extra-embryonic tissues and of spatially localised signalling in gastruloids: mammalian embryonic organoids. *Development* 144, 3894-3906. 10.1242/dev.150391 28951435 PMC5702072

[DEV202983C97] Tzouanacou, E., Wegener, A., Wymeersch, F. J., Wilson, V. and Nicolas, J.-F. (2009). Redefining the progression of lineage segregations during mammalian embryogenesis by clonal analysis. *Dev. Cell* 17, 365-376. 10.1016/j.devcel.2009.08.002 19758561

[DEV202983C98] Varelas, X., Sakuma, R., Samavarchi-Tehrani, P., Peerani, R., Rao, B. M., Dembowy, J., Yaffe, M. B., Zandstra, P. W. and Wrana, J. L. (2008). TAZ controls Smad nucleocytoplasmic shuttling and regulates human embryonic stem-cell self-renewal. *Nat. Cell Biol.* 10, 837-848. 10.1038/ncb1748 18568018

[DEV202983C99] Varoquaux, N. and Purdom, E. (2020). A pipeline to analyse time-course gene expression data. *F1000Res.* 9, 1447. 10.12688/f1000research.27262.1

[DEV202983C100] Veenvliet, J. V., Bolondi, A., Kretzmer, H., Haut, L., Scholze-Wittler, M., Schifferl, D., Koch, F., Guignard, L., Kumar, A. S., Pustet, M. et al. (2020). Mouse embryonic stem cells self-organize into trunk-like structures with neural tube and somites. *Science* 370, eaba4937. 10.1126/science.aba4937 33303587

[DEV202983C101] Vianello, S. and Lutolf, M. P. (2020). In vitro endoderm emergence and self-organisation in the absence of extraembryonic tissues and embryonic architecture. *bioRxiv* 2020.06.07.138883. 10.1101/2020.06.07.138883

[DEV202983C102] Vincent, S. D., Dunn, N. R., Hayashi, S., Norris, D. P. and Robertson, E. J. (2003). Cell fate decisions within the mouse organizer are governed by graded Nodal signals. *Genes Dev.* 17, 1646-1662. 10.1101/gad.1100503 12842913 PMC196136

[DEV202983C103] Viotti, M., Nowotschin, S. and Hadjantonakis, A.-K. (2014). SOX17 links gut endoderm morphogenesis and germ layer segregation. *Nat. Cell Biol.* 16, 1146-1156. 10.1038/ncb3070 25419850 PMC4250291

[DEV202983C104] Warin, J., Vedrenne, N., Tam, V., Zhu, M., Yin, D., Lin, X., Guidoux-D'Halluin, B., Humeau, A., Roseiro, L., Paillat, L. et al. (2024). In vitro and in vivo models define a molecular signature reference for human embryonic notochordal cells. *iScience* 27, 109018. 10.1016/j.isci.2024.109018 38357665 PMC10865399

[DEV202983C105] Warmflash, A., Sorre, B., Etoc, F., Siggia, E. D. and Brivanlou, A. H. (2014). A method to recapitulate early embryonic spatial patterning in human embryonic stem cells. *Nat. Methods* 11, 847-854. 10.1038/nmeth.3016 24973948 PMC4341966

[DEV202983C106] Winzi, M. K., Hyttel, P., Dale, J. K. and Serup, P. (2011). Isolation and characterization of node/notochord-like cells from mouse embryonic stem cells. *Stem Cells Dev.* 20, 1817-1827. 10.1089/scd.2011.0042 21351873 PMC3928718

[DEV202983C107] Wise, C. A., Sepich, D., Ushiki, A., Khanshour, A. M., Kidane, Y. H., Makki, N., Gurnett, C. A., Gray, R. S., Rios, J. J., Ahituv, N. et al. (2020). The cartilage matrisome in adolescent idiopathic scoliosis. *Bone Res.* 8, 13. 10.1038/s41413-020-0089-0 32195011 PMC7062733

[DEV202983C108] Wisniewski, D., Lowell, S. and Blin, G. (2019). Mapping the emergent spatial organization of mammalian cells using micropatterns and quantitative imaging. *J. Vis. Exp.* 146. 10.3791/59634 31107437

[DEV202983C118] Wong, F. C. K. (2021). Whole-mount immunofluorescence staining of early mouse embryos. In: *Epigenetic Reprogramming During Mouse Embryogenesis. Methods in Molecular Biology* (K. Ancelin and M. Borensztein eds), Vol. 2214, pp. 143-155. Springer. 10.1007/978-1-0716-0958-3_10 32944908

[DEV202983C109] Wymeersch, F. J., Skylaki, S., Huang, Y., Watson, J. A., Economou, C., Marek-Johnston, C., Tomlinson, S. R. and Wilson, V. (2019). Transcriptionally dynamic progenitor populations organised around a stable niche drive axial patterning. *Development* 146, dev168161. 10.1242/dev.168161 30559277 PMC6340148

[DEV202983C110] Wymeersch, F. J., Wilson, V. and Tsakiridis, A. (2021). Understanding axial progenitor biology in vivo and in vitro. *Development* 148, dev180612. 10.1242/dev.180612 33593754

[DEV202983C111] Xu, P.-F., Borges, R. M., Fillatre, J., De Oliveira-Melo, M., Cheng, T., Thisse, B. and Thisse, C. (2021). Construction of a mammalian embryo model from stem cells organized by a morphogen signalling centre. *Nat. Commun.* 12, 3277. 10.1038/s41467-021-23653-4 34078907 PMC8172561

[DEV202983C112] Yamamoto, M., Meno, C., Sakai, Y., Shiratori, H., Mochida, K., Ikawa, Y., Saijoh, Y. and Hamada, H. (2001). The transcription factor FoxH1 (FAST) mediates Nodal signaling during anterior-posterior patterning and node formation in the mouse. *Genes Dev.* 15, 1242-1256. 10.1101/gad.883901 11358868 PMC313795

[DEV202983C113] Yamanaka, Y., Tamplin, O. J., Beckers, A., Gossler, A. and Rossant, J. (2007). Live imaging and genetic analysis of mouse notochord formation reveals regional morphogenetic mechanisms. *Dev. Cell* 13, 884-896. 10.1016/j.devcel.2007.10.016 18061569

[DEV202983C114] Yasuo, H. and Lemaire, P. (2001). Role of Goosecoid, Xnot and Wnt antagonists in the maintenance of the notochord genetic programme in Xenopus gastrulae. *Development* 128, 3783-3793. 10.1242/dev.128.19.3783 11585804

[DEV202983C115] Zhang, Y., Zhang, Z., Chen, P., Ma, C. Y., Li, C., Au, T. Y. K., Tam, V., Peng, Y., Wu, R., Cheung, K. M. C. et al. (2020). Directed differentiation of notochord-like and nucleus pulposus-like cells using human pluripotent stem cells. *Cell Rep.* 30, 2791-2806.e5. 10.1016/j.celrep.2020.01.100 32101752

[DEV202983C116] Zhao, C., Plaza Reyes, A., Schell, J. P., Weltner, J., Ortega, N. M., Zheng, Y., Björklund, Å. K., Baqué-Vidal, L., Sokka, J., Torokovic, R. et al. (2024). A comprehensive human embryogenesis reference tool using single-cell RNA-sequencing data. *Nat. Methods* 10.1038/s41592-024-02493-2 PMC11725501 39543283

