# Supplementary material for: In vitro modelling of anterior primitive streak patterning with human pluripotent stem cells identifies the path to notochord progenitors
Source: Development. 2024 Dec 12;151(24):dev202983. doi: 10.1242/dev.202983 (PMC11664173; doi:10.1242/dev.202983)
Supplement: Supplementary information [file develop-151-202983-s1.pdf]

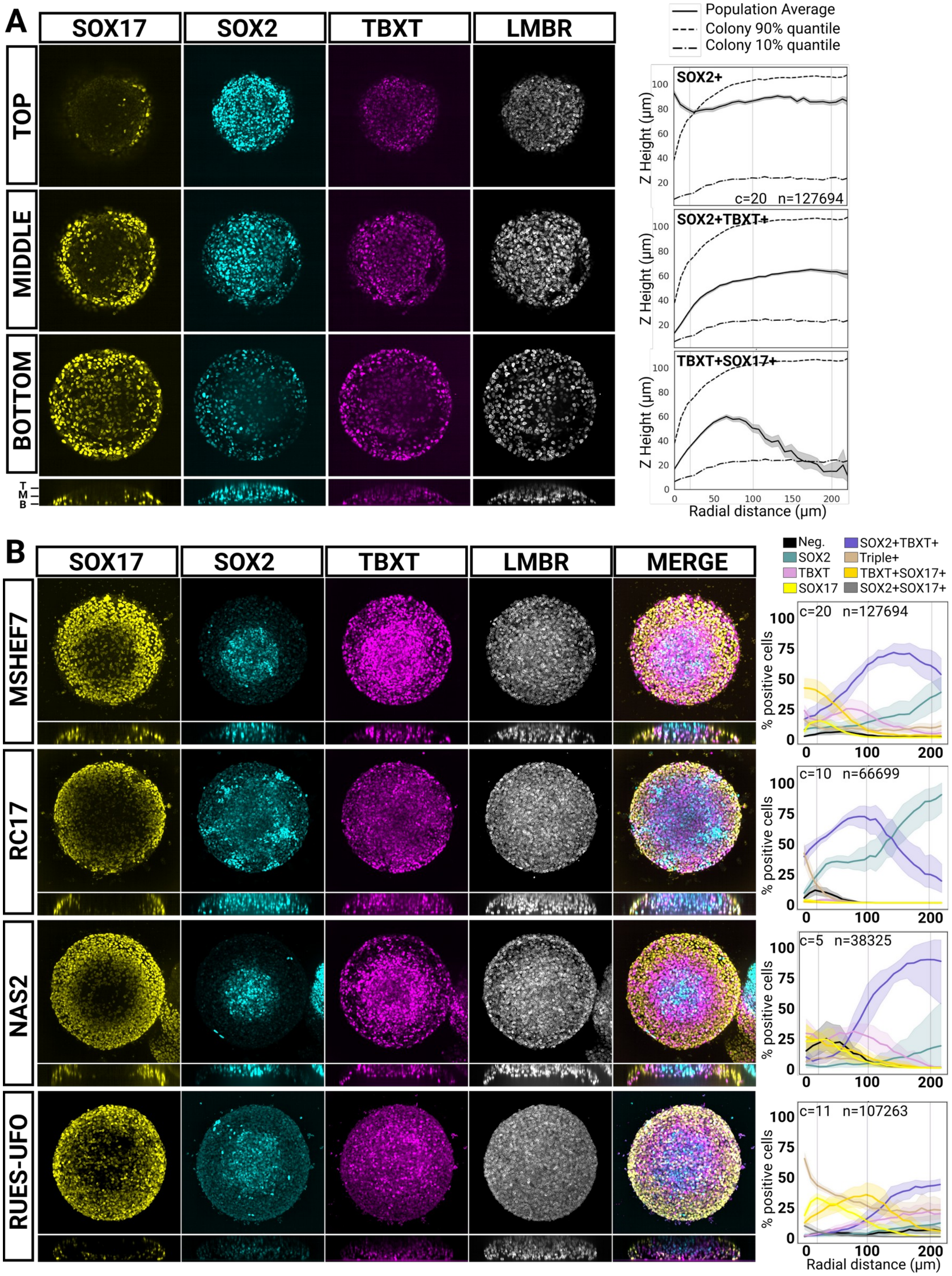

Fig. S1. Cell fate patterning variability across multiple pluripotent cell lines. A Representative example of a 500µm micropatterned hESC colony (MasterShef 7 cell line) treated for 48h with 2µM CHIR and 20ng/ml FGF2. Individual confocal planes at 3 different depths are provided for each staining. Images show how SOX2+ cells are positioned at the top of the colony while SOX17+ cells are most abundant at the periphery but can also

Development • Supplementary information

be found more centrally at the bottom of the colony. Quantifications of the average position of individual cell populations along the radial distance from the colony edge are shown on the right. **B** SOX17, SOX2 and TBXT distributions in 500µm colonies at 48h post CHIR/FGF treatment in different human pluripotent cell lines. Note that MShef7, RC17 and NAS2 cells were stained via immunofluorescence for all markers while RUES-UFO cells were immunostained only for LMBR. The other channels show the fluorescence of the reporter (SOX17-tdTomato, SOX2-mCitrine, TBXT-mCerulean). Images are max intensity projections and representative of at least 2 independent experiments. Quantifications of the radial profiles of the percentage of cell types are shown on the right. c: number of analysed colonies, n: number of analysed nuclei, shaded area: 95% confidence interval

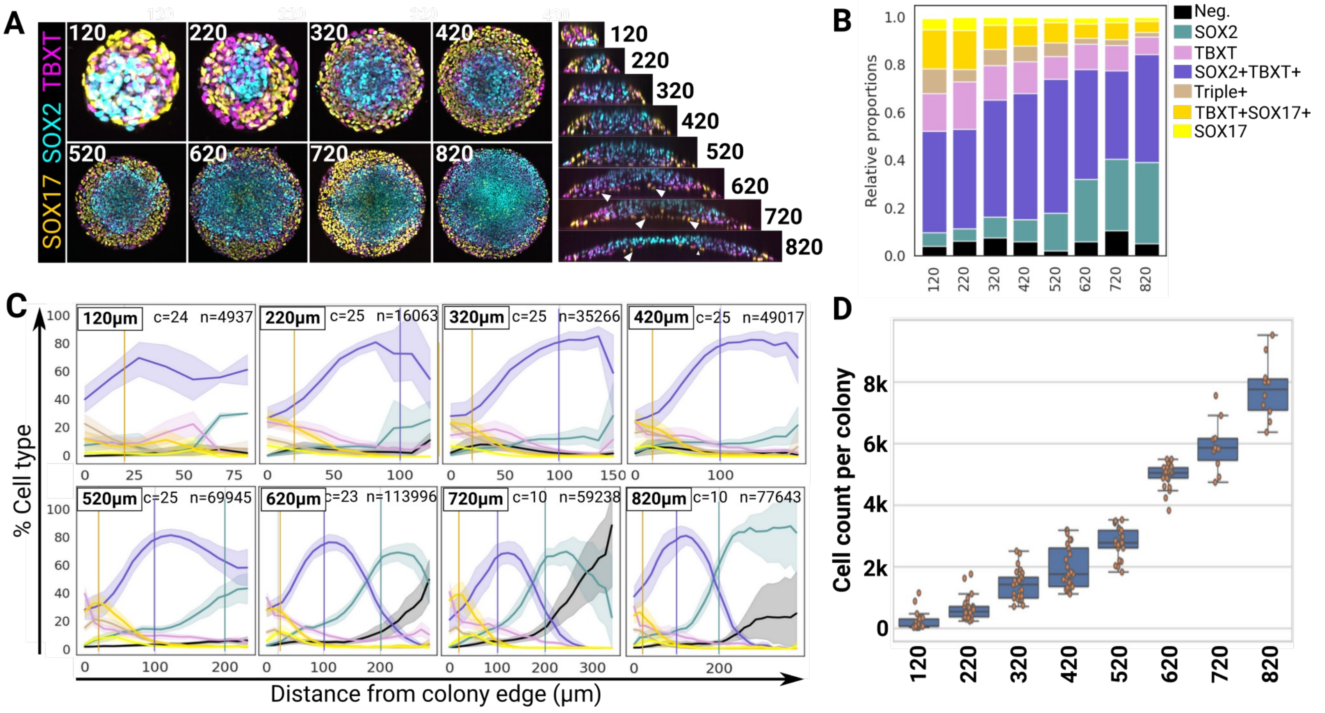

Fig. S2. Cell fate patterning does not scale with colony size. A Confocal max projections of micropatterned colonies of increasing diameters (indicated in the top left corner in µm) and stained for SOX17, SOX2 and TBXT. The corresponding Z projections are shown on the right with white arrowheads pointing at SOX17+ cells lining the central domain at the bottom of the colony. **B** Stacked bar plot showing the relative proportions of individual cell populations found for each colony diameter. The number of colonies and nuclei analysed for this plot are the same as in iii. **C** Line plots showing the mean proportion of each cell population as a function of the radial distance from the edge of the colonies. c: number of analysed colonies, n: number of analysed nuclei, shaded area: 95% confidence interval. **D** Beeswarm box plot showing the number of cells per colony. Each dot represent one colony. Colony diameters are indicated on the x axis in µm.

Development • Supplementary information

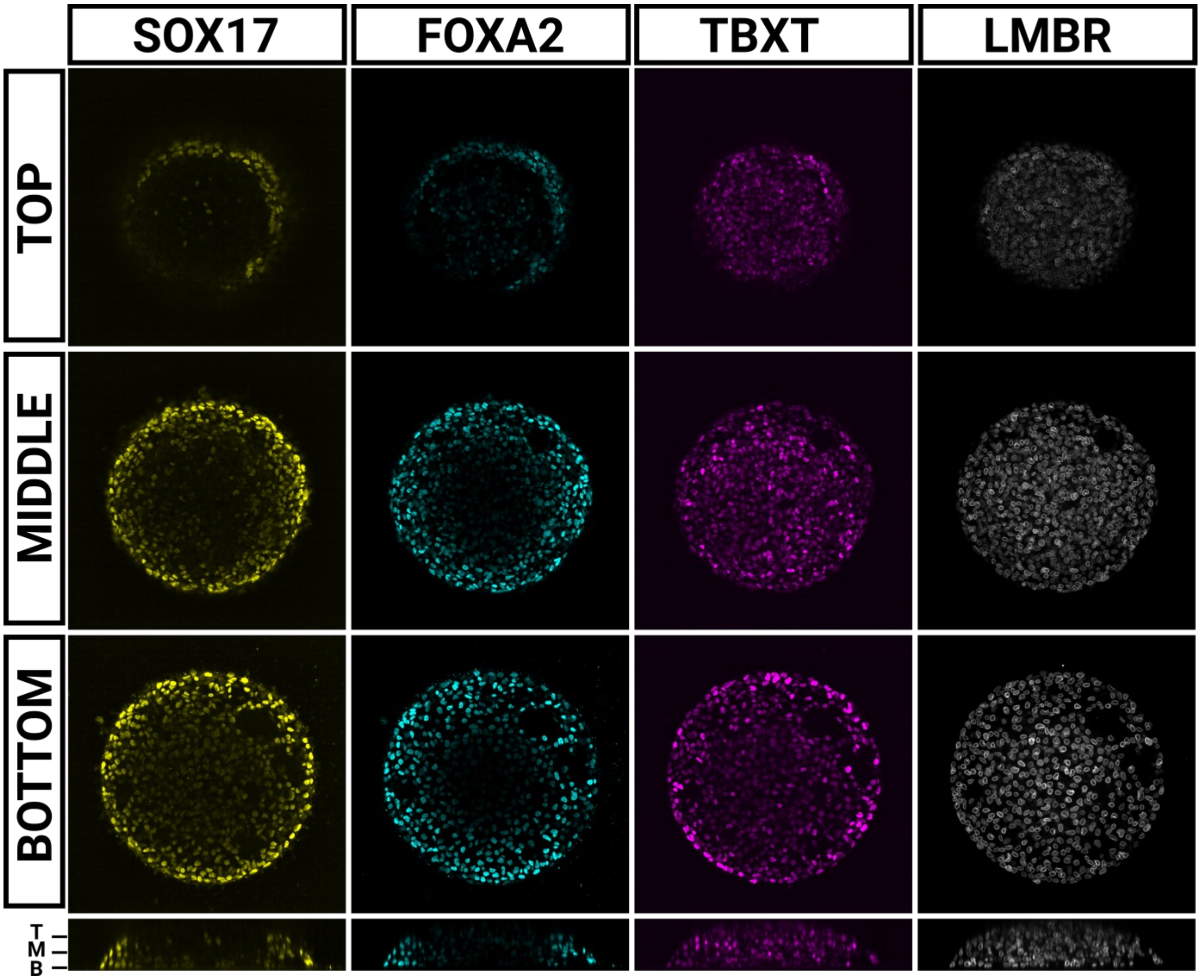

Fig. S3. Representative example of a 500µm micropatterned hESC colony (MasterShef 7 cell line) treated for 48h with 2µM CHIR and 20ng/ml FGF2. Individual confocal planes at 3 different depths are provided for each staining as well as a z-projection (bottom row)

Development • Supplementary information

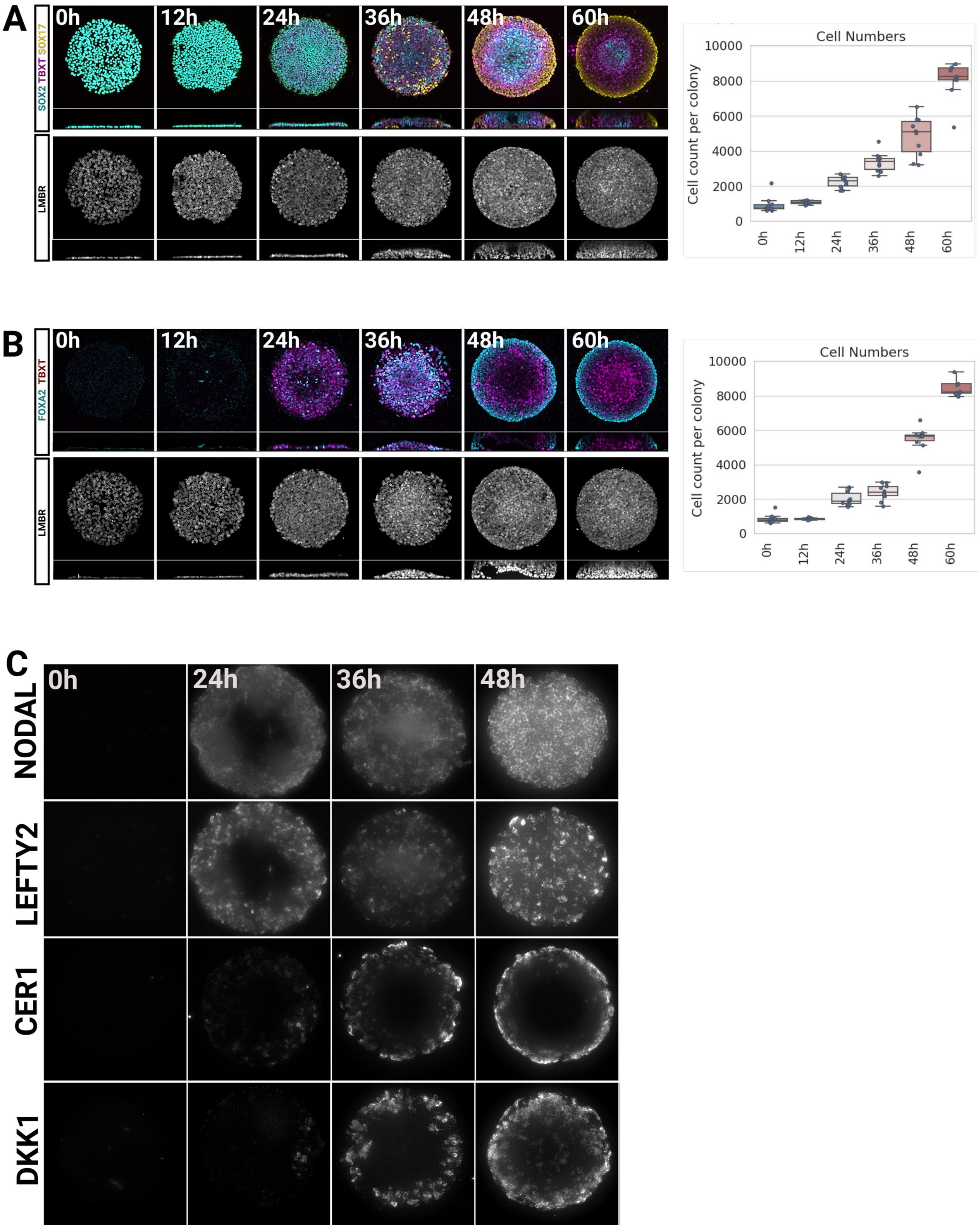
A
0h
12h
24h
36h
48h
60h
SOX2 TBXT SOX17
LMBR
Cell Numbers
Cell count per colony
10000
8000
6000
4000
2000
0
0h
12h
24h
36h
48h
60h
B
0h
12h
24h
36h
48h
60h
FOXA2 TBXT
LMBR
Cell Numbers
Cell count per colony
10000
8000
6000
4000
2000
0
0h
12h
24h
36h
48h
60h
C
0h
24h
36h
48h
NODAL
LEFTY2
CER1
DKK1

Fig. S4. Time course analysis of cell fate markers and signalling molecules expression. A and B Time course analysis of 500µm colonies via immunofluorescence. Images are max intensity projections of 3D confocal z-stacks and were taken from a distinct experimental replicate than in Fig 2 B and E. A nuclear envelope counterstaining is also included together with a z-axis projection underneath each main image. Notice how colonies form a monolayer until 24h and then start forming a dome like structure with multiple cell layers on top of one another from 36h onwards. We sometimes observed that the middle of the colonies lifted slightly off the pattern (see the z-axis projection for the TBXT/FOXA2/LMBR staining at 48h). This phenomenon happened during the staining procedure. A boxplot representing the cell number per colony is shown on the right. **C** Widefield images of 500µm colonies stained via branched DNA FISH at selected time points.

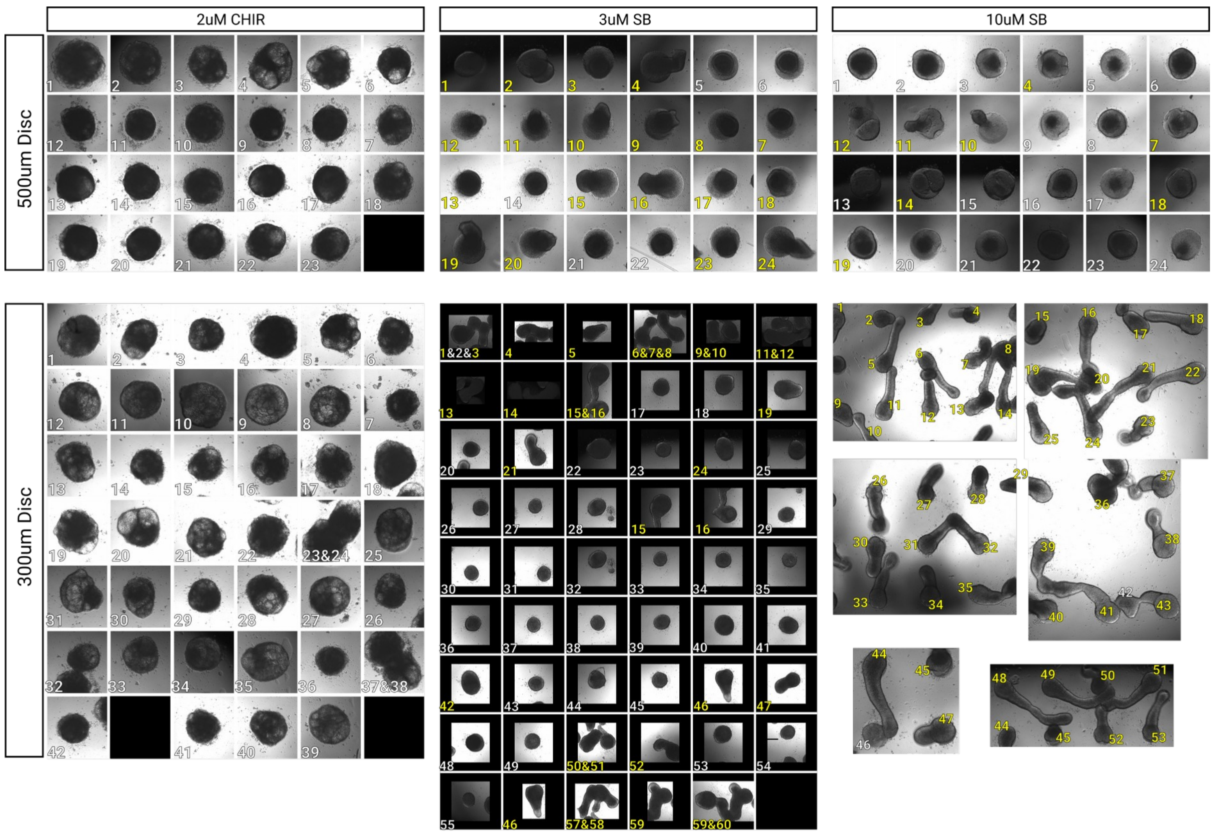

Colony Diameter : 500 µm			
**SB Concentration**	**0 µM**	**3µM**	**10 µM**
**Total number of colonies**	23	24	24
**Number of elongated colonies**	0	19	8
**% elongated colonies**	0.00%	79.17%	33.33%

Colony Diameter : 300 µm			
**SB Concentration**	**0 µM**	**3 µM**	**10 µM**
**Total number of colonies**	42	60	53
**Number of elongated colonies**	0	28	51
**% elongated colonies**	0.00%	46.67%	96.23%

Fig. S5. Manual quantification of the percentage of elongated colonies 6 days post-seeding on micropatterns. Cells were treated for 3 days with 2µM CHIR and 20ng/ml FGF2 in N2B27 medium and then for 3 more days in unsupplemented medium with or without the NODAL inhibitor SB added throughout the experiment. Bright field images show different colonies grown on micropatterns of either 300µm or 500µm diameter and treated with different doses of SB as indicated. A yellow number indicate that the colony was counted as “elongated” and a white number as “non-elongated”. The number of analysed colonies and the percentage of elongated colonies is shown in the tables below for each condition.

Development • Supplementary information

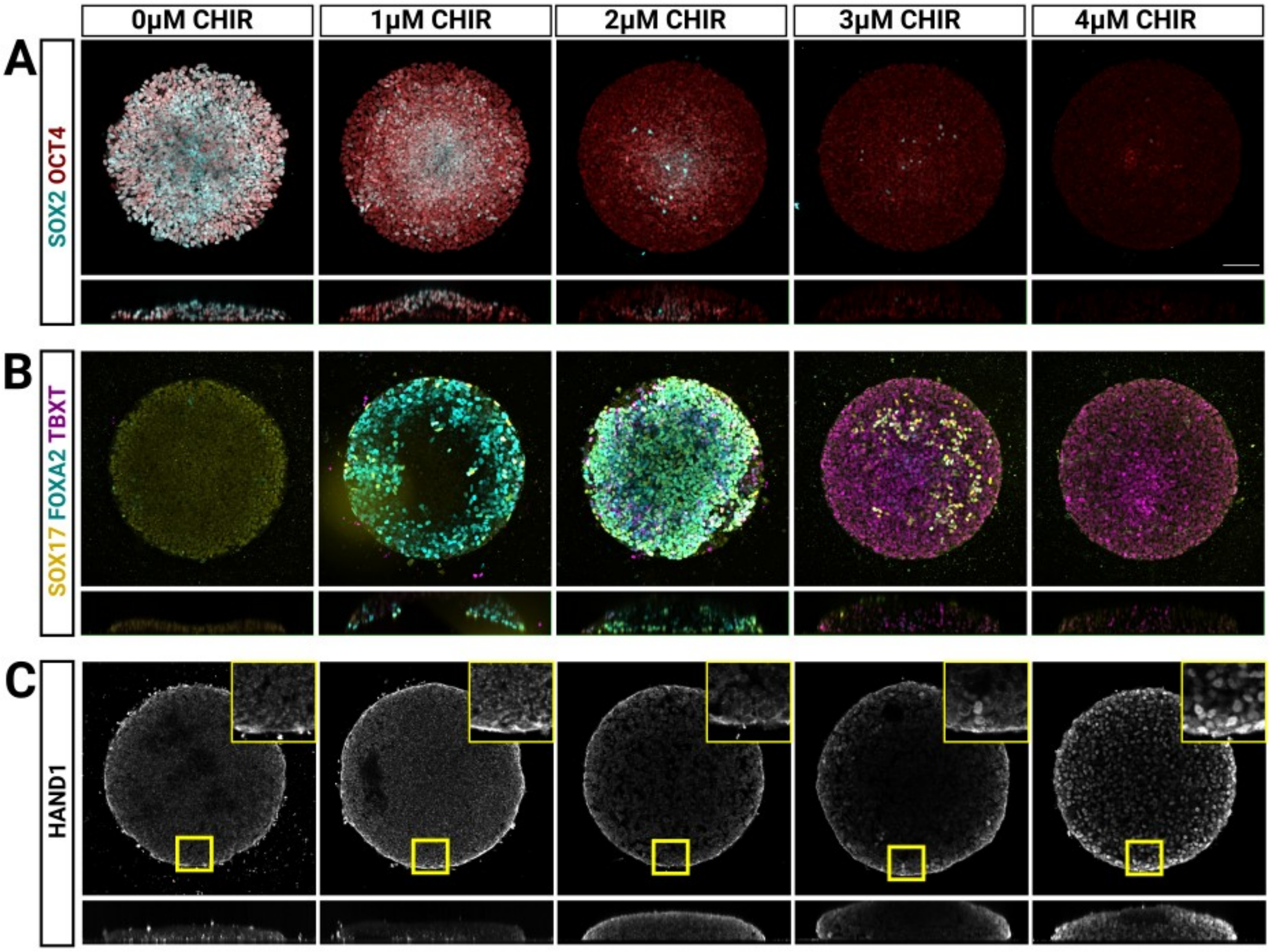

Fig. S6. CHIR dose-response in 500µm colonies 48h post-induction. A and B include representative max projections of confocal z-stacks analysed in Figure 4B while **C** shows one selected z-slice across representative confocal z-stacks to better show the nuclear localisation of HAND1.

Development • Supplementary information

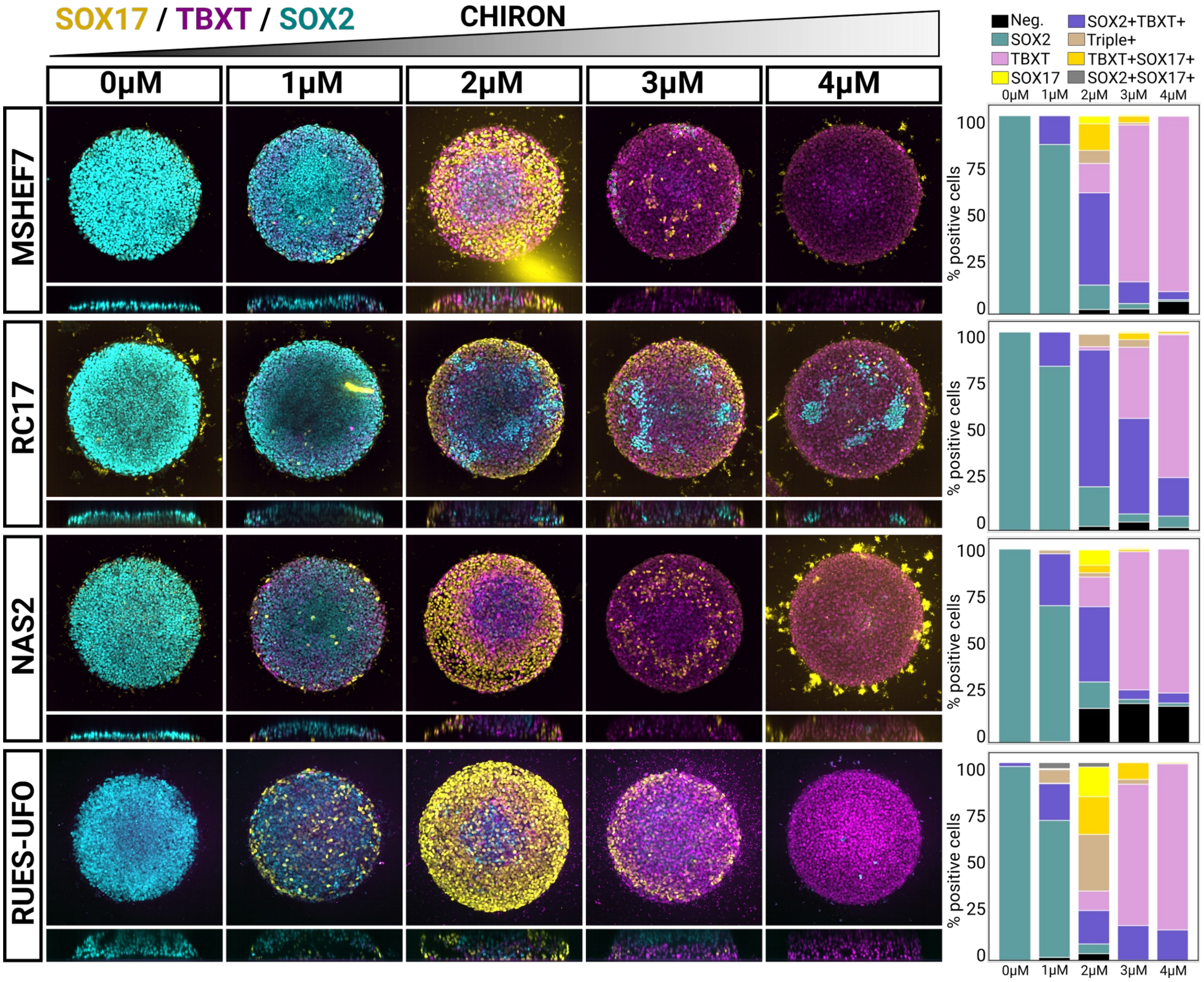

Fig. S7. CHIR dose response in 500μm colonies fixed at 48h across different human pluripotent cell lines. Images show the max projections of confocal z-stacks and a z-projection underneath. Stacked bar plot showing the relative proportions of individual cell populations for each CHIR concentration and each cell line is provided on the right hand side. As in supplementary figure 1, MShef7, RC17 and NAS2 cells were stained via immunofluorescence for all markers while RUES-UFO cells were immunostained only for LMBR (other channels show the fluorescence of the reporters: SOX17-tdTomato, SOX2-mCitrine, TBXT-mCerulean). Notice how the SOX17 signal peaks at 2μM in all cell lines, while higher CHIR concentrations lead the vast majority of the cells to become positive only for TBXT. We observed that the behaviour of MShef7 and NAS2 cells on micropatterns aligned almost exactly. Interestingly, RUES-UFO generated a much higher abundance of SOX17+ cells, perhaps because their endogenous signalling profile is biased towards NODAL activity. We also observed a less well-defined domain of SOX2+ cells at 2μM in the RC17 line. Patches of SOX2+ cells were also maintained at 3 and 4μM CHIR in this cell line. We have not further investigated why different cell line behaves slightly differently but it may be insightful to do so in the future.

Development • Supplementary information

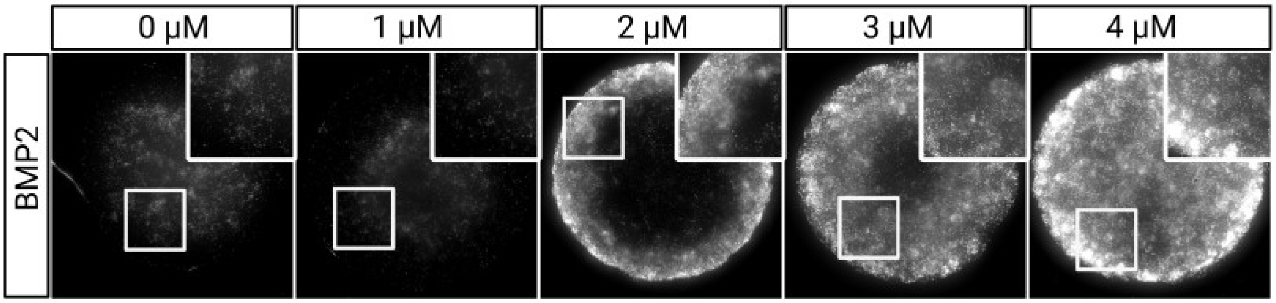

Fig. S8. Representative widefield images of FISH staining against BMP2 transcripts in 500µm colonies 48h post-induction treated with 20ng/ml FGF2 and a range of CHIR concentrations indicated at the top of the images.

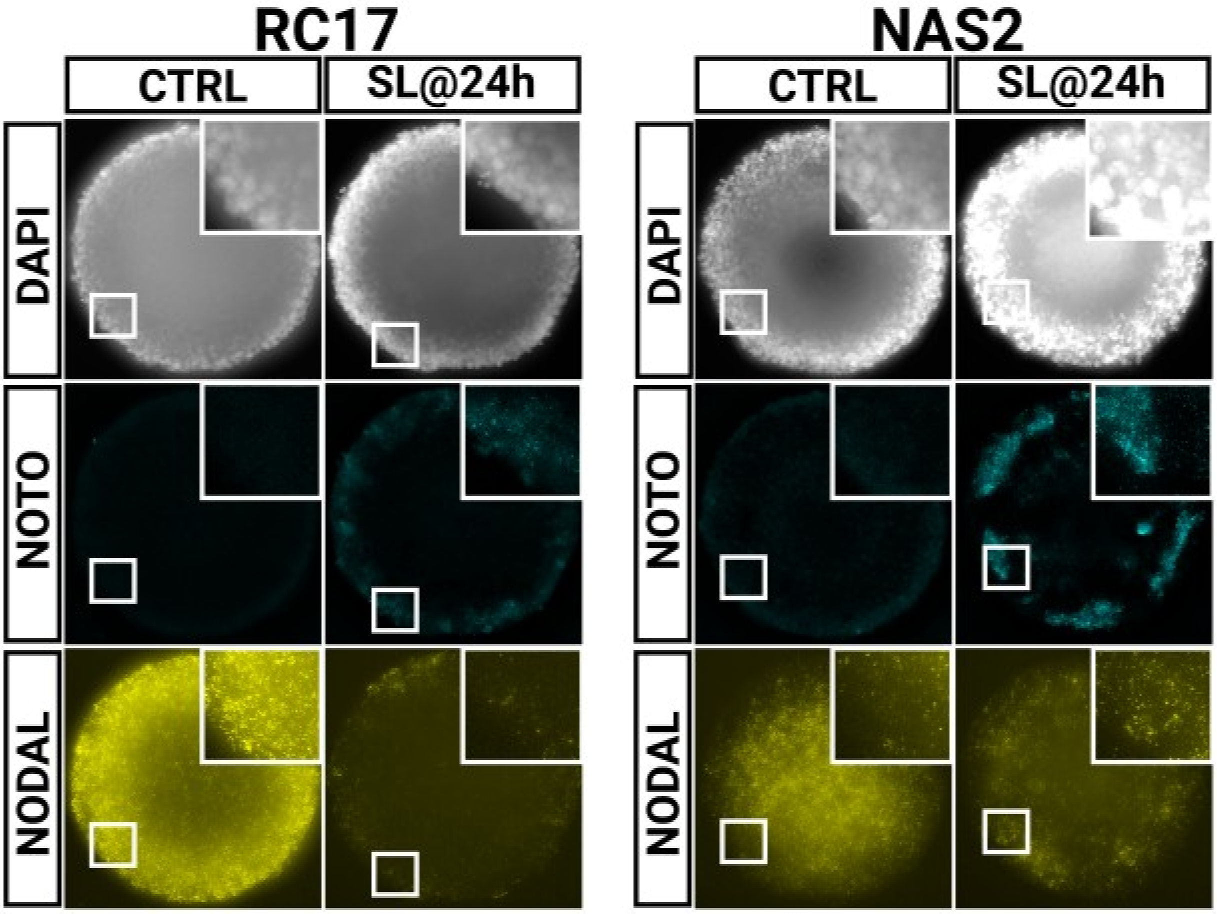

Fig. S9. Representative widefield images of FISH staining against NOTO and NODAL transcripts in 500µm colonies at 48h of NotoPs induction (2µM CHIR, 20ng/ml FGF2 throughout and 10µM SB and 0.1µM LDN added at 24h). The data shows NOTO+ cells emerging at the periphery of colonies of 2 different cell lines. Please note that this staining is not possible in the RUES-UFO cell line due to the fluorescence of the reporter.

Development • Supplementary information

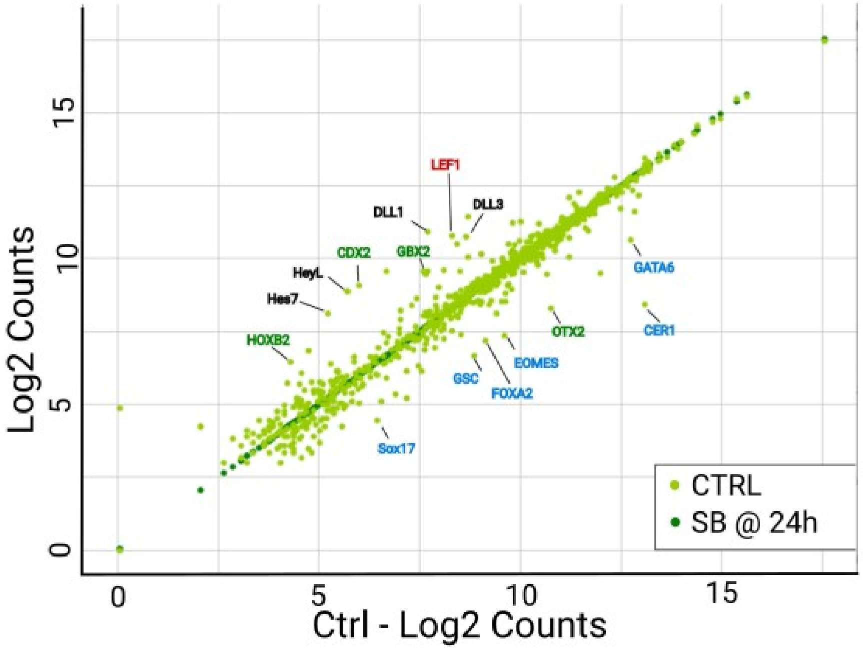

Fig. S10. Nanostring analysis of hESC grown on micropatterns in the presence of 2µM CHIR and 20ng/ml FGF2 for 2 days with or without 10µM SB added at 24h.

Development • Supplementary information

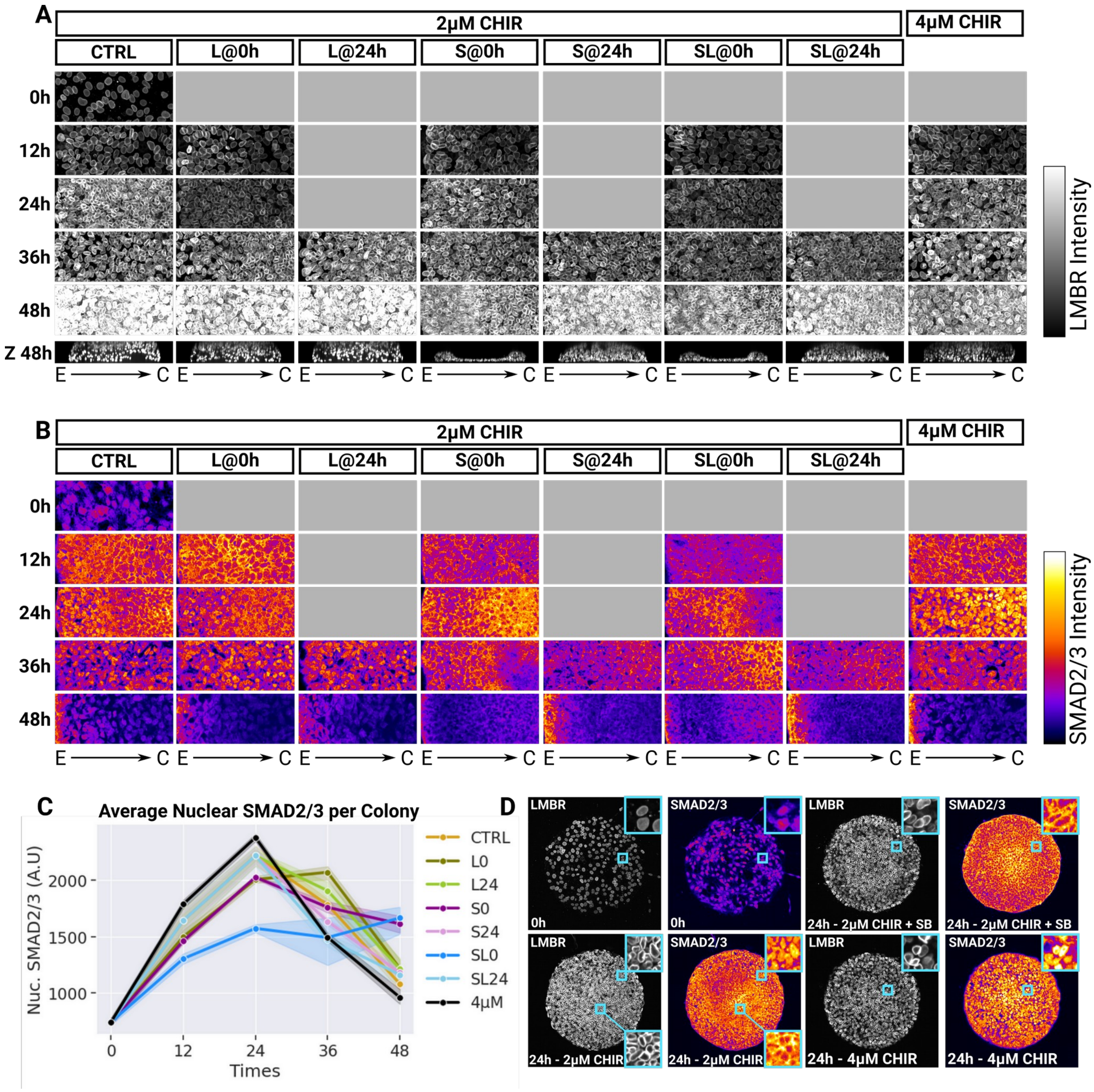
A
2μM CHIR
4μM CHIR
CTRL
L@0h
L@24h
S@0h
S@24h
SL@0h
SL@24h
0h
12h
24h
36h
48h
Z 48h
E → C
LMBR Intensity
B
2μM CHIR
4μM CHIR
CTRL
L@0h
L@24h
S@0h
S@24h
SL@0h
SL@24h
0h
12h
24h
36h
48h
E → C
SMAD2/3 Intensity
C
Average Nuclear SMAD2/3 per Colony
Nuc. SMAD2/3 (A.U)
2000
1500
1000
0
12
24
36
48
Times
CTRL
L0
L24
S0
S24
SL0
SL24
4μM
D
LMBR
SMAD2/3
0h
24h - 2μM CHIR + SB
24h - 2μM CHIR
24h - 4μM CHIR

Fig. S11. Time course analysis of SMAD2/3 expression in 500µm hESC colonies. Images are taken from the same experiment shown in Fig 7 and show representative image crops as illustrated in Fig 5A. The positioning of the colony edge (E) and centre (C) is indicated. **A** Max intensity projection of the LMBR signal and z-axis projection (bottom row) showing the colony morphology. **B** Representative single confocal planes showing SMAD2/3 signal. Notice how SB treatment results in exclusion of the SMAD2/3 signal from the nucleus. **C** Line plot showing the temporal profiles or average nuclear SMAD2/3 intensity within entire colonies. The shaded area indicate the 95% confidence interval of mean nuclear intensity across colonies. Images on the right. **D** Representative confocal images of selected conditions showing the specificity of the SMAD2/3 signal. Notice how SMAD2/3 is found nuclear at the periphery of the colonies with 2µM CHIR but remain cytoplasmic at the centre of the same colony or in the colony treated with 10µM SB. S: 10µM SB, L: 100nM LDN, SL: combination of SB and LDN treatment.

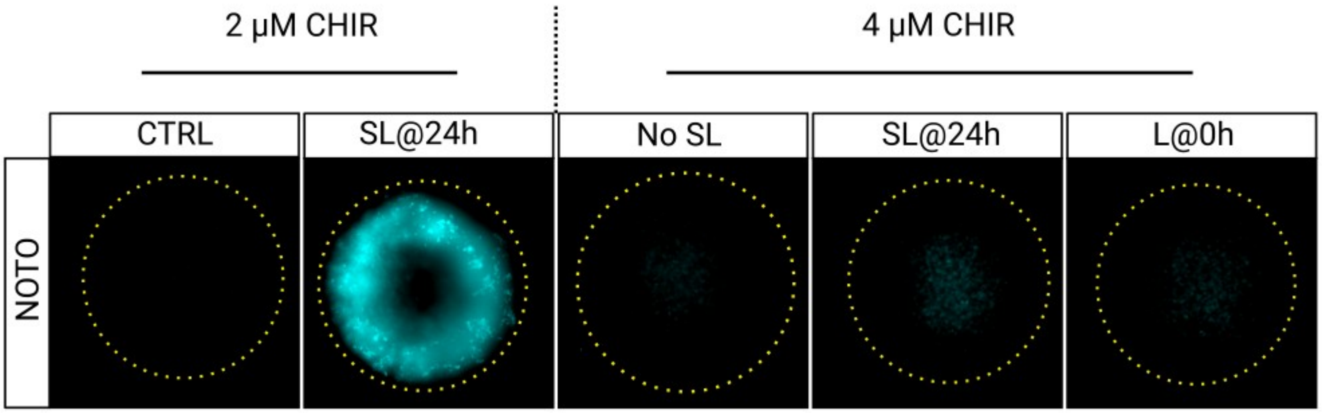

Fig. S12. Representative widefield images of branched-DNA FISH staining against NOTO transcripts in 500μm colonies at 48h of differentiation. SL: 10μM SB and 100nM LDN. L: 100mM LDN only.

Table S1. List of NanoString probes

Available for download at
https://journals.biologists.com/dev/article-lookup/doi/10.1242/dev.202983#supplementary-data

Development • Supplementary information

Table S2. List of primary antibodies used in this work

Primary Antibody	Cat. no, supplier	Dilution
SOX2	14-9811-82, Thermo Fisher Scientific	1:200
TBX6	AF4744, R&D Systems	1:400
PAX6	561462, BD Bioscience	1:300
TBXT	NL2085R Conjugated, R&D Systems	1:300
SOX17	AF1924, R&D Systems	1:200
CDX2	Ab76541, Abcam	1:200
OCT4	AP3724A, Abcepta	1:200
FOXA2	8186, Cell Signalling	1:400
SMAD2/3	610842, BD Bioscience	1:100
SOX9	Ab185230 Conjugated, Abcam	1:250
CDH1	SIGMA, MABT26	1:100
CDH2	BD Bioscience, 610182	1:200

Table S3. List of secondary antibodies used in this work

Secondary Antibody	Cat. no, supplier	Dilution
Donkey-Anti-Goat-405	Abcam, ab175665	1:1000
Donkey-Anti-Mouse-405	Abcam, ab175658	1:1000
Donkey-Anti-Rat-488	Abcam, ab150153	1:1000
Donkey-Anti-Mouse-488	Abcam, ab150105	1:1000
Donkey-Anti-Rabbit-A568	Abcam, ab175470	1:1000
Donkey-Anti-Mouse-568	Abcam, ab175472	1:1000
Donkey-Anti-Rabbit-647	Abcam, ab150075	1:1000
Donkey-Anti-Rat-647	Abcam, ab150155	1:1000
Donkey-Anti-Mouse-647	Abcam, ab150107	1:1000

Table S4. List of FISH probes used in this work

FISH Probe-Channel	Cat.no
NOTO-488	VA4-3101015-VC
CHRD-546	VA1-3005411-VC
NODAL-647	VA6-3167894-VC
LEFTY2-546	VA1-3004742-VC
DKK1-488	VA4-3082344-VC
CER1-488<br>BMP2-647	VA4-3089614-VC<br>VA6-3168104-VC
