# Supplementary material for: In vitro modelling of anterior primitive streak patterning with human pluripotent stem cells identifies the path to notochord progenitors
Source: Development. 2024 Dec 12;151(24):dev202983. doi: 10.1242/dev.202983 (PMC11664173; doi:10.1242/dev.202983)
Supplement: Supplementary file 1 [file develop-151-202983_review_history.pdf]

In vitro modelling of anterior primitive streak patterning with human pluripotent stem cells identifies the path to notochord progenitors

Miguel Robles-Garcia, Chloe Thimonier, Konstantina Angoura, Ewa Ozga, Heather MacPherson and Guillaume Blin
DOI: 10.1242/dev.202983

Editor: Matthias Lutolf

Review timeline

Original Submission:	13 June 2023
Editorial Decision:	17 July 2023
Resubmission:	23 April 2024
Editorial Decision:	28 May 2024
First Revision Received:	23 August 2024
Editorial Decision:	10 October 2024
Second Revision Received:	24 October 2024
Accept:	20 November 2024

Original submission

First decision letter

MS ID#: DEVELOP/2023/202083
MS TITLE: In vitro modelling of anterior primitive streak patterning with hESC reveals the dynamic of WNT and NODAL signalling required to specify notochord progenitors

AUTHORS: Miguel Robles Garcia, Chloe Thimonier, Konstantina Angoura, Ewa Ozga, Heather MacPherson, and Guillaume Blin
ARTICLE TYPE: Research Article

I have now received all the referees reports on the above manuscript, and have reached a decision. The referees' comments are appended below, or you can access them online: please go to BenchPress and click on the 'Manuscripts with Decisions' queue in the Author Area.

As you will see from their reports, the referees recognise the potential of your work, but they also raise significant concerns about it. Given the nature of these concerns, I am afraid I have little choice other than to reject the paper at this stage.

However, having evaluated the paper, I do recognise the potential importance of this work. I would therefore be prepared to consider as a new submission an extension of this study that contains new experiments, data and discussions and that address fully the major concerns of the referees. The work required goes beyond a standard revision of the paper. Please bear in mind that the referees (who may be different from the present reviewers) will assess the novelty of your work in the context of all previous publications, including those published between now and the time of resubmission.

Author's response to the reviewer's comments

© 2024. Published by The Company of Biologists under the terms of the Creative Commons Attribution License (https://creativecommons.org/licenses/by/4.0/).

Response to reviewers

We thank both reviewers for their time and helpful criticisms. We have made substantial changes to our manuscript since our last submission and summarise these below.

1) We overhauled Fig 1 to better document how confinement biases cell fates towards the endodermal and mesodermal lineages. We show in this figure that colonies eventually form organised 3-dimensional structures which are mainly composed of endoderm and mesodermal cells with a core of epithelial SOX2 positive cells. In light of these new data we no longer claim that the cells undergo “limited axial elongation” in our starting conditions and focus on the fact that confinement leads to a distinct outcome than 2D monolayer culture.

2) We provide new data to reinforce the idea that inhibition of endogenous NODAL signalling is necessary for NMP emergence on micropatterns and axial elongation (new Fig 3B and C). We notably show that successful axial elongation is a function of both colony size and extent of NODAL inhibition.

NB: We have removed all mentions of the acronym “hAXIOMs” to avoid any confusion since our study presents data on a range of conditions that do not necessary lead to the formation of an axis.

3) We now include a full time course analysis of NODAL and WNT signalling across all our conditions in dedicated figures (Fig 5 and Fig 7). Overall, our analyses document a complex non-linear dependence between CHIR concentration and endogeneous NODAL signalling dynamics, thus reinforcing our initial claims. Our data also unveil a signalling cross talk between the NODAL and WNT pathways which may reinforce the notochordal lineage (Fig 7). We clarify our thinking about how these signalling dynamics may regulate lineage restrictions in the APS and propose a hypothetical model summarised in Fig 8.

4) We reproduced our finding in two other pluripotent cell lines (Sup Fig. 1 and Sup. Fig. 5) demonstrating the robustness of our findings. We also show that we can efficiently generate NOTO+ cells by growing cells on lines (200µm in width) which maximise the proportions of cells experiencing edge effects and therefore NotoPs (Fig. 6C). To our knowledge, this is the only report showing high abundance of NOTO+ cells in hPSCs-derived colonies in the absence of genetic manipulation and simply via control of the biochemical and mechanical environment of the cells.

5) Finally, our figures include quantifications wherever appropriate and we updated our text to include relevant literature published during the preparation of this revised manuscript.

Reviewer 1 Advance Summary and Potential Significance to Field:

This paper does not inspire confidence since there are a number of significant issues that are unexplored or passed over. The text attempts to put the best light on what they found, but its far from a complete picture. I do not recommend publication.

We appreciate this reviewer’s time and useful criticisms. We apologise for the lack in clarity in the original version of our manuscript and we hope that the substantial revisions that we have now implemented will inspire confidence for publication.

Major issues

1) Fig 1C: Do cells lift off the surface in the center of larger micropatterns (MP)? It sure looks that way. The same phenomena is also visible in Fig 3C 1-10muM SB (and their standard 500 diameter). This is never commented upon. Given the importance the MS attaches to time restricted Nodal signaling, this seems quite relevant to interpretations in Fig 3. If the cells are all attached, then we need see a DAPI stain. Also by 48h the cell density in the center seems like 5-6 nuclei deep, is this a columnar epithelium or really multiple layers. It would be informative to see a side view at earlier times beginning with induction.

We were also a bit puzzled by this observation at first and did not observe this across all our

© 2024. Published by The Company of Biologists under the terms of the Creative Commons Attribution License (https://creativecommons.org/licenses/by/4.0/).

experiments. We believe that the lifting of the colony centre off the micropattern surface occurs during the staining procedure and does not reflect the behaviour of the colony prior fixation. This phenomenon occurred more frequently with larger micropatterns. This may or may not be biologically relevant and therefore we chose not to comment on this in the manuscript. However, **as requested by this reviewer, we now provide a nuclear staining in Supplementary figure 3 across all time points in order to document how the colonies change shape and adopt a 3-dimensional structure over time**. As shown below, we do not observe the formation of a clear columnar epithelium in 500µm colonies but we do observe evidence of complex morphogenetic events taking place in these structures. It will be interesting to study these events in more details in a future study (as commented in our discussion L453-459). Please note that we intend to deposit all our high resolution images (including larger colonies) in a public repository allowing interested readers to browse and explore our images freely.

As discussed further below (point 5), we have now considerably strengthened our analysis of NODAL signalling (now Fig 5). Please note that our analysis was performed in 500µm colonies which remain attached on the micropatterns even after staining.

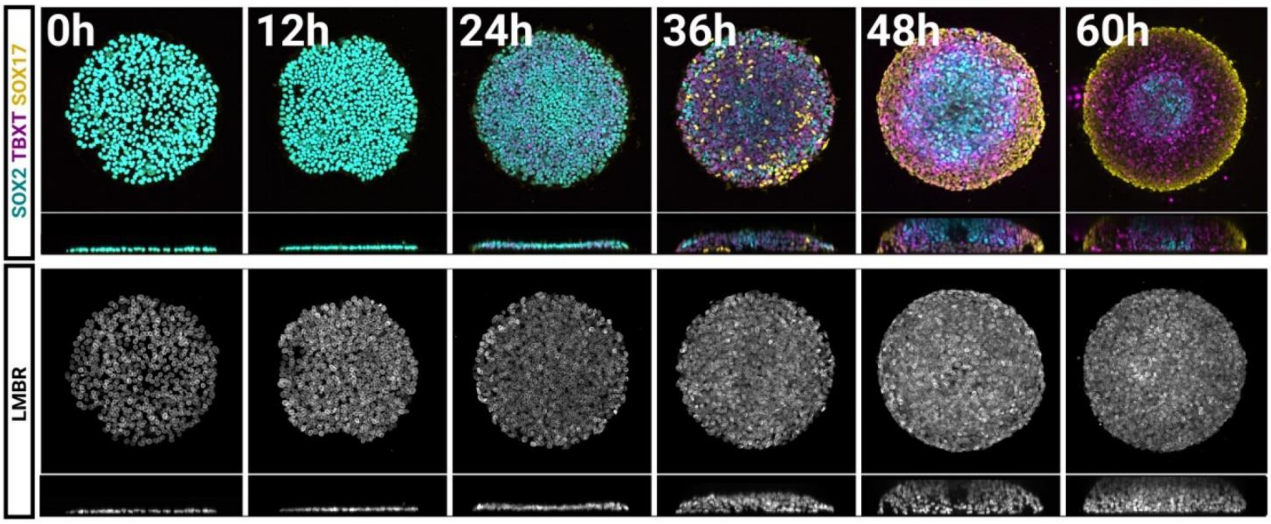

2) Fig 1G: Do cells migrate out of the MP (first visible 42h and substantial 72h). The gene expression seems to break the rotational symmetry of the MP with the Sox2 on one end and the Sox17 on the other. Is this typical? Again never commented.

We have now removed Fig1G as we agree that it did lack clarity. This old figure is pasted below for reference.

We would like to clarify that the cells never left the boundary of the micropatterns. The small scattered objects that appear around the main structure in the images bellow are cell debris excluded from the colony. We also agree that the original widefield immunofluorescence image showing SOX17, SOX2 and TBXT at 72h was confusing (on the right hand side of the figure). The SOX2 positive cells which appeared outside of the micropattern are in fact located above the plane of focus as the colony starts to grow upwards. But again the cells did not exit the confine of the micropattern and SOX17 remained located all around the periphery of the colony.

We now provide clearer high resolution images of colonies stained at day 5 of differentiation in Fig 1F. We also clarified our message in the main text and explain that when endogenous NODAL signalling is left unchecked, confinement biases cell fates towards the endoderm and mesoderm lineages. We also provide stronger evidence that NODAL inhibition is required in order to induce robust uniaxial growth on micropatterns in Fig 3 B and C.

© 2024. Published by The Company of Biologists under the terms of the Creative Commons Attribution License (https://creativecommons.org/licenses/by/4.0/).

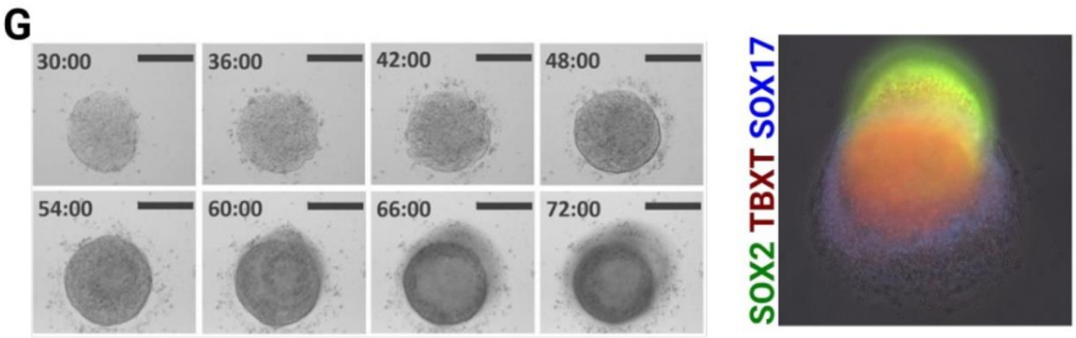

3) Fig 2C: The text says the pSmad23 at 24h is edge, but I see it everywhere (and nicely nuclear). There are a minority of strong staining cells in the outer 1/3, 1/2 of radius. It would be nice to see a histogram of intensities and how many cells are high. The stain looks uniform at 36h and its certainly not background, since its a lot less at 48h.

We have now considerably strengthened our analysis of NODAL (and WNT) signalling, please see our response to point 5 below.

4) L 214 Sox2 clear on at 48h in stains (eg Fig 1C), but its part of a cluster that is OFF at 24h, is this just persistent protein?

We agree that this observation may look surprising but our Nanostring experiment was performed on bulk RNA extracts. We can assume that the total number of SOX2 transcripts declined proportionally to the decrease in the number of cells expressing SOX2. (~ 25% at 48h compared to 100% at 0h - Fig 2B). Furthermore the half-life of SOX2 protein has been reported to be around 8h in mouse embryonic stem cell (Liu et al 2017 NCB doi: 10.1038/ncb3474) we can therefore expect a delay in the downregulation of the protein.

5) Fig 3C: I do not understand the pSmad23 staining in Fig 2C in relation to the NODAL expression in Fig 3B (is this image at 48h like the others? its not stated). Firstly there is black, red, yellow-white in the in-situs, with NODAL-text in red. Is the red to white an intensity scale? The spatial patterns at 1-2muM are virtually complementary and 2muM is the concentration in Fig 2C where the authors would like to say (L277) its edge, but the NODAL expression is center! So perhaps the NODAL expression varies in time dramatically, in which case we need to see that. This is another instance where something odd in the data is passed over.

We apologise for the lack of clarity in our original manuscript and thank the reviewer for the useful suggestion. We now include a full time course analysis of NODAL signalling in the new Fig 5. We also incorporated diagrams and colour scales to improve the clarity of the figure (Fig 5A). Both FISH against NODAL transcripts (5C) and immunofluorescence for SMAD2/3 (5E) were quantified. The average expression across the colony is plotted in Fig 5C and radial profile of intensities are shown as heatmaps below the corresponding images (Fig 5D and E). This figure is now described between L288 and L336.

Overall, our analysis documents a complex non-linear dependence between CHIR concentration and NODAL expression dynamics. In light of these new data we have slightly amended the phrasing of our claim regarding NODAL dynamics and endoderm specification:
We remain descriptive in the result section – L332: “a slow and sustained increase in NODAL expression together with a low expression of LEF1 was found in colonies which formed endoderm and a sharp transient expression of NODAL together with a strong increase in LEF1 was found in colonies where mesoderm was the most abundant”.
Later on in the manuscript we also document a crosstalk between NODAL and WNT signalling (Fig 7). We now clarify our thinking in the discussion - L501: “We propose a model where initial WNT and NODAL signalling combinatorially specify epiblast cells to an early multipotent APS state (co-

© 2024. Published by The Company of Biologists under the terms of the Creative Commons Attribution License (https://creativecommons.org/licenses/by/4.0/).

expressing TBXT and FOXA2) which can commit to either endoderm or notochord. While prolonged NODAL exposure keeps WNT activity low and drives endoderm differentiation, a sharp inhibition of NODAL signalling potentiates WNT activity and redirects the cells to the NotoP fate (Fig 8B)."

We note also that our quantification agrees with our initial claim that nuclear SMAD2/3 tends to be higher at the periphery than in the centre of the colony with 2µM CHIR at 24h (Fig 5E 2µM - radial intensities). But we also acknowledge that the picture is indeed rather complex L321-329.

Overall, we believe that our new data considerably strengthen our claims that small variations in CHIR concentration induce distinct signalling dynamics of endogenous NODAL which correlate with cell fate outcomes. We also note that our data agree and are with complementary to recent results reported in a preprint by another group using a Nodal live reporter and WNT ligands instead of CHIR (Ortiz-Salazar et al 2024 BiorXiv) - discussed L470-475. This further confirms the robustness of our findings.

Please see also our response to reviewer 2 point 6

L 238 "Noto markers incl SLIT2 on at 36h", with some references. But these are mouse, and are they plausibly time matched? The 2023 paper mentions mouse pulposus, which in the intro is described as the fate of notocord. Generally the paper fishes out references for expression of this or that in a variety of species with no critical thought.

We now provide another reference supporting the fact that both SLIT2 and CAV1 are expressed in the human embryonic notochord around week 8 of development (Warin et al iScience 2024 doi: https://doi.org/10.1016/j.isci.2024.109018).
More generally, we tried to better reference human studies wherever possible and clarified in the text which model organisms the studies we reference have been using. These are indicated in a blue font in the manuscript.

We also provide this reviewer with a dot plot obtained from a human embryogenesis reference scRNAseq dataset (Zhao et al 2023 BiorXiv) available as an interactive tool from the Petropoulos website (https://petropoulos-lanner-labs.clintec.ki.se/app/ShinyEmbryoRef). This figure shows the expression pattern of the markers mentioned in our study and corroborate our claims.

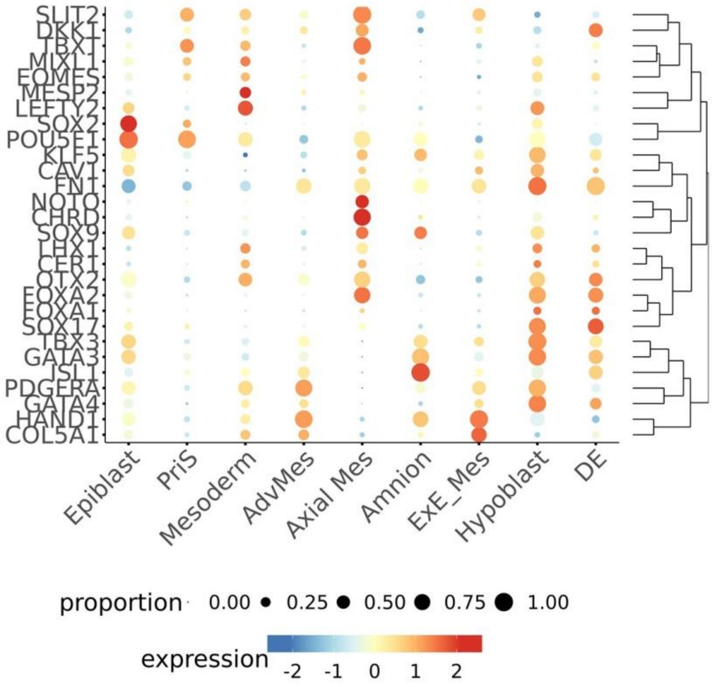

© 2024. Published by The Company of Biologists under the terms of the Creative Commons Attribution License (https://creativecommons.org/licenses/by/4.0/).

6) L 364-382 Comes the bombshell: CHIR only operates in their context to induce endogenous WNT (a IWP2 secretion block, shuts down all down stream fates). But they put a favorable spin on it L381 !. But what are we to make of biological relevance if CHIR and Wnt are so different. Of course CHIR is not effected by the endogenous Wnt inhibitors that should come on in some space+time pattern. So if Wnt is operative, what is the space+time pattern that is conducive to notocord?

Fig 3D, IWP2 exposure: The desired conclusion is 0-24h is the crucial period but what we are shown is 24-48h minor effect, 0-48h big effect and we are supposed to take the difference??

Apologies for the confusion – we did not claim that IWP2 shuts down all downstream fates but that endogenous WNT was important for endodermal specification in this context. We share clearer images of colonies stained at 48h below (not included in the manuscript) to show that IWP2 treatment prevents SOX17 expression and expands the SOX2 domain. We include the 0-24h treatment period as well as a TBXT staining to show that there is little or no effect of IWP2 treatment on TBXT expression. It is possible that IWP2 treatment delays differentiation. While we think these data are interesting, we decided not to include these findings in the current version of the manuscript as we now think that these observations raise additional questions that would distract the reader too much from the main focus of our manuscript. We hope to elucidate the mechanisms underlying these observations in a follow up study.

NOTE: We have removed unpublished data that had been provided for the referees in confidence.

7) Fig 4; They continue with CHIR treatments and now inhibit Nodal after 24h. In 4E they note strong and uniform upregulation of Chordin.

We apologise for the confusion. Our initial Fig 4E showed a staining for LEF1 (a target gene of the WNT pathway) to demonstrate that NODAL inhibition potentiates WNT activity. We now provide a dedicated figure (Fig 7) to show this effect and include quantifications. Please see also our response to point 5.

Is CHRD expressed in the notocord (as well as the node?) How about NOGGIN?

In mice, the expression of both chordin and noggin transcripts have been shown to overlap in the node and the notochord (Bachiller et al 2000, Nature, https://doi.org/10.1038/35001072). We show here in a human context that CHRD is co-expressed with NOTO when the cells are treated for 48h with CHIR and FGF and with SB and LDN added at 24h (Fig 6A). This provide good evidence that the cells have engaged into the notochord fate.

8) The mouse embryo sections in 4C are very difficult to place, there are no structural landmarks and just localized staining of 3 genes. I presume they are sagittal sections?

This reviewer is correct, the confocal image in (now Fig 6 F) represents a sagittal view of the distal side of an early-bud mouse embryo. This is clarified in the legend. We also annotated the figure to indicate the orientation of the antero-posterior axis as well as the position of the low-Tbra positive primitive streak.

Small Items:

Probably not a good idea to use hESC in title and abstract without a definition.

Thank you, we have now change the title to: “In vitro modelling of anterior primitive streak patterning with human pluripotent stem cells identifies the path to notochord progenitors”.

Fig1H coloring of neural vs mesendo is reversed between the in-vivo cartoon and the MP stains for Sox2, Tbx6

Thank you, we have now tried to keep our colour scheme consistent across the manuscripts and

© 2024. Published by The Company of Biologists under the terms of the Creative Commons Attribution License (https://creativecommons.org/licenses/by/4.0/).

thoroughly proof-read the manuscript to avoid spelling errors and omissions.

Reviewer 2 Advance Summary and Potential Significance to Field:
In this manuscript, the authors use an established hPSC culture technique (micropatterning) to study the molecular mechanisms involved in the complex regulation of cell identity specification and patterning of the Anterior Primitive Streak (APS). Among the mesodermal derivatives of the APS, the notochord has been particularly challenging to derive in vitro from hPSC. The authors claim that their optimized system allows for the derivation of bona fide notochord progenitors (NotoP). They also draw important conclusions regarding APS specification and patterning via the use of this system.

In more detail, the authors show that:

1) The stimulation of micropatterned hPSC with CHIR99021 promotes the formation of cell fate patterns as reported in other articles. A definitive endodermal layer is found at the periphery, and moving inwards a mesodermal layer, a putative neuromuscular progenitor (NMP) layer and a neuroepithelial layer follow. Interestingly, the authors reported that the colonies developed a tridimensional structure reminiscent of axial elongation. For this reason, they call this in vitro model hAXIOMs.Despite the identification at intermediate time points of possible precursors of NotoPs, no NotoPs were detected under this condition at the final time point.

2) The stimulation of the canonical WNT pathway with CHIR99021 activates the expression of NODAL and subsequently the patterned expression of its own inhibitor Cerberus1 (CER1) and of the WNT inhibitor DKK1. This is in line with many reports on very similar culture systems and more in general with reports on hPSC differentiation.Titrating CHIR99021 dose the authors found interesting differences in NODAL expression pattern. These patterns seem to correlate with the localization of different cell fates along the colonies axis.

3) Dampening the cell response to endogenously produced NODAL, the authors were able to highlight the requirement of high TFGb pathway activity for the acquisition of endodermal fate, and of an intermediate activity for mesodermal and NMP fate.

4) The timed inhibition/reduction of TGFb and BMP pathways activity allows for APS cells in the system to progress towards a NotoPs state (cells expressing CHORDIN and NOTO).

The authors make use of a hPSC culture system already reported in many studies, but the work of the authors place a novel focus on the patterning of APS. In particular, the authors show the generation of notochord progenitors, a cell population that has been very rarely seen and characterized in hPSC models. They also use the system to try to disentangle the signaling dynamics that cause the observed cell fate pattern.

For these reasons, I think that the manuscript is suited for this journal and of interest to the journal's audience. Nonetheless, I feel many important points have not been addressed satisfactory. I suggest a round of revision. Below I report some suggestions for strengthening the authors' hypothesis.

We thank the reviewer for their thorough revision of the manuscript and helpful constructive suggestions.

Reviewer 2 Comments for the Author:

MAJOR COMMENTS
1) The authors performed all the experiments with a single hESC cell line (MasterShef7).
The key experiments should be repeated on ideally other two lines. **At minimum the protocol for the derivation of NotoPs should be reproducible in other lines.** It is also possible that different lines require different concentrations of CHIR99021 and SB431542 compared to MasterShef7. That will be also interesting to report.

© 2024. Published by The Company of Biologists under the terms of the Creative Commons Attribution License (https://creativecommons.org/licenses/by/4.0/).

We thank the reviewer for this suggestion. We have now reproduced our results with two other cell lines RC17 (hESC line) and NAS2 (hIPSC line). For both cell lines, we show endoderm emergence on micropatterns with 2µM CHIR and 20ng/ml FGF treatment for 48h (Sup Fig 1) and we show NOTO+ cells when SB and LDN are added at 24h (Sup Fig 6). We note that using the same concentrations of CHIR99021 and SB431542 were sufficient to reproduce the results obtained with the MasterSheff7 line.

2) The authors used Nanostring as a way to quantify gene expression for a wide subset of human genes. This technique returns only bulk information. In such a system, the information obtained with scRNAseq would be relevant in disentangling the relationships between different cell types and in getting more insights on the dynamics of cell fate specification. Moreover, **scRNAseq data can be analyzed via RNA velocity or similar methods to understand the developmental trajectories of the different cell types.**
I think this type of data would greatly enrich the work of the authors.

We agree with the reviewer here that scRNAseq data would be very helpful to determine cell identities and that RNA velocity could be useful to infer putative lineage trajectories. We also think that lineage tracing should be done to definitively validate our model shown in Fig 8. However we also think that these experiments should be the focus of a follow-up article and we hope to obtain the resources to perform these costly analyses in the near future. In the meantime, we have incorporated new Nanostring data in Fig 4C to further document changes in RNA transcript levels across CHIR conditions and validated HAND1 expression at the protein level via immunofluorescence (Fig 1F and Sup Fig 4C). We hope the reviewer will agree that our study already provides a breadth of novel data and information sufficient on their own to justify a publication in Development.

3) The authors report that their model recapitulates aspects of axial elongation. In Fig.1I they show that the center of the colony seems to elongate. They call their model hAXIOMs in virtue of these phenomena. Unluckily, the characterization of this phenotype is minimal. I would like to see more detailed pictures of this elongated structure with the positioning of the different cell types.

As mentioned earlier, we have now revised our claims and removed mentions of the hAXIOMS acronym.
1) Fig 1 is now focused on documenting how confinement leads to a distinct outcome than 2D monolayer culture and redirects cell fates towards endoderm. We provide a new side by side comparison of monolayer versus micropatterned cultures in Fig 1B.We also include new staining and 3D confocal images of the colonies at day 5 to document how colonies eventually develop (Fig 1F). These images show how colonies reproducibly develop into organised 3D structures, composed mainly of endoderm and mesodermal cells with a core of epithelial SOX2 positive cells. These images also show how the cells generate their own extra cellular matrix, including fibronectin and collagen V. The text was substantially revised to reflect these changes L146-159. Please also note that we will deposit all our high resolution images in a public repository allowing interested readers to browse and explore our images freely.

4) The molecular characterization of the signaling pathway activity needs to be extended. The pictures of SMAD2/3 nuclear translocation of Fig.2C are not so clear. More pictures with higher magnification are needed. A quantification of the pathway activity is also needed. The authors should consider to check for pSMAD3 or pSMAD2 staining as a way to monitor TGFb pathway activity.

We thank the reviewer for the suggestions. Unfortunately, in spite of several attempts, we were unable to obtain reliable phospho SMAD2 or 3 staining in our cultures. However, we considerably expanded our analysis of NODAL transcripts and nuclear SMAD2/3 levels. This is now presented in a new dedicated figure where our analysis covers the range of CHIR concentrations that we tested (Fig 5). Please see also our response to point 6 below.

5) What is the time point for the NODAL staining in Fig. 3B? If the time point is the same of the cell

© 2024. Published by The Company of Biologists under the terms of the Creative Commons Attribution License (https://creativecommons.org/licenses/by/4.0/).

fate staining in Fig. 3A, I find it hard to reconcile the results. The signaling events that contributed to the cell fate pattern would be the ones of a previous time point. Please comment on this point.

We apologise for the lack of clarity in our initial manuscript. The staining presented in our original version was taken at 48h and we agree with the reviewer that the signalling events that contributed to cell fate patterning would be the one at previous time points.

We address this point in our response to point 6.

6) The authors discuss how different CHIR99021 doses result in different patterning outcomes in virtue of a different patterning of NODAL expression and likely TGFb pathway activity. First, it would be nice to check TGFb pathway activity (SMAD2/3 nuclear translocation or pSMAD2/3 signal) upon the treatment with different CHIR99021 doses. Does it match the pattern of NODAL expression? Second, the authors claim that "strong sustained NODAL specifies endoderm". But observing the results for 1uM and 2um CHIR99021 in Fig. 3Ai and Fig. 3B, I can see that the 1uM condition has intense NODAL in the periphery and no NODAL in the center of the colony. This results (Fig. 3Ai) in very few SOX17+ cells at the extreme periphery. On the other hand, a milder expression of NODAL in the periphery upon 2uM CHIR99021 stimulation results in a large peripheral area of SOX17+ cells. That area seems to match the area of mild NODAL expression in the periphery.
How do the authors reconcile these results in their model?

We agree that clarity in the text and additional data were needed to address these issues. (As this point is similar to point 5 from reviewer 1, part of our answer to reviewer 1 is duplicated here in purple but please see also our response to reviewer 1 point 5)

We now include a full time course analysis of both WNT and NODAL signalling across the range of CHIR doses in Fig 5. We also incorporated diagrams and colour scales to improve the clarity of the figure (Fig 5A). Both FISH against NODAL transcripts (5C) and immunofluorescence for SMAD2/3 (5E) were quantified. The average expression across the colony is plotted in Fig 5C and radial profile of intensities are shown as heatmaps below the corresponding images (Fig 5D and E). This figure is now described between L288 and L336.

Looking at the profiles of NODAL (Fig 5C ii) and SMAD2/3 (Fig 5C iii) we can see that 1µM CHIR than at 2µM CHIR lead to a sustained increase in NODAL expression and longer maintenance of nuclear SMAD2/3. This correlates with endoderm emergence in these colonies. On the other hand, higher CHIR concentration lead to sharp increase followed by a rapid downregulation of NODAL expression.

In light of these new data we have changed our phrasing regarding NODAL dynamics and endoderm specification. In particular, we no longer claim that a "strong NODAL activity is needed for endoderm emergence" but instead that a **sustained increase in NODAL expression** correlates with endoderm emergence.

L332: "a slow and sustained increase in NODAL expression together with a low expression of LEF1 was found in colonies which formed endoderm. On the other hand, a sharp transient expression of NODAL together with a strong increase in LEF1 was found in colonies where mesoderm was the most abundant".

We note also that our quantification agrees with our initial claim that nuclear SMAD2/3 tends to be higher at the periphery than in the centre of the colony with 2µM CHIR **at 24h** (Fig 5E 2µM - radial intensities). But we also acknowledge that the picture is indeed rather complex and discuss this matter in details L321-329.

Overall, we believe that our new data clarify the dependence of endoderm emergence to a sustained exposure to NODAL signalling.
We also note that our data agree and are complementary with findings recently reported in a preprint by another group using a Nodal live reporter line and WNT ligands instead of CHIR (Ortiz-Salazar et al 2024 BiorXiv) - discussed L470-475. This further confirms the robustness of our

© 2024. Published by The Company of Biologists under the terms of the Creative Commons Attribution License (https://creativecommons.org/licenses/by/4.0/).

findings.

MINOR COMMENTS

1) The authors report that SOX17+ cells form a sparse epithelial layer lining the entire bottom of the colony. Although this is not the main focus of this manuscript, I find this result very interesting. It would be great to have more detailed pictures of this phenomenon (the ones reported are at too low magnification to appreciate it clearly). Moreover, a quantification of the number of SOX17+ cells located below the colony across multiple samples/colonies would highlight the robustness of this phenotype.

In confidence to this reviewer, we are currently preparing a manuscript describing how tissue topology influences the migratory behaviour of endodermal cells emerging in this system. As this is a minor aspect of the present article we did not include further images in our figures. However, we will provide our high resolution images in a public repository.

2) On line 148 correct “320uM” to “320um”.

Than you, the manuscript has been extensively revised and thoroughly proof read to avoid spelling errors and omissions.

Resubmission

First decision letter

MS ID#: DEVELOP/2024/202983

MS TITLE: In vitro modelling of anterior primitive streak patterning with human pluripotent stem cells identifies the path to notochord progenitors

AUTHORS: Miguel Robles-Garcia, Chloe Thimonier, Konstantina Angoura, Ewa Ozga, Heather MacPherson, and Guillaume Blin

I have now received all the referees' reports on the above manuscript, and have reached a decision. The referees' comments are appended below, or you can access them online: please go to BenchPress and click on the 'Manuscripts with Decisions' queue in the Author Area.

As you will see, the referees express considerable interest in your work, but have some significant criticisms and recommend a substantial revision of your manuscript before we can consider publication. If you are able to revise the manuscript along the lines suggested, which may involve further experiments, I will be happy receive a revised version of the manuscript. Your revised paper will be re-reviewed by one or more of the original referees, and acceptance of your manuscript will depend on your addressing satisfactorily the reviewers' major concerns. Please also note that Development will normally permit only one round of major revision. If it would be helpful, you are welcome to contact us to discuss your revision in greater detail. Please send us a point-by-point response indicating your plans for addressing the referees’ comments, and we will look over this and provide further guidance.

Please attend to all of the reviewers' comments and ensure that you clearly highlight all changes made in the revised manuscript. Please avoid using 'Tracked changes' in Word files as these are lost in PDF conversion. I should be grateful if you would also provide a point-by-point response detailing how you have dealt with the points raised by the reviewers in the 'Response to Reviewers' box. If you do not agree with any of their criticisms or suggestions please explain clearly why this is so.

Reviewer 1

© 2024. Published by The Company of Biologists under the terms of the Creative Commons Attribution License (https://creativecommons.org/licenses/by/4.0/).

Advance summary and potential significance to field

-

Comments for the author

The authors have made substantial improvement to the work and I see it now suitable for publication.

Below I have some comments and suggestions that the authors can address at their discretion. These points will not affect the main messages of the authors' manuscript:

1) Line 102 "SOX2-only domain in the center and a SOX17 domain at the periphery (Fig. 1B and 1C)"
From the pictures the cells seem to be almost all SOX17/TBXT positive. Maybe reflect this in the text.

2) Fig. 1E; I see 6 curves/lines in the plot. Is the brown line for FOXA/SOX17? In the legend is indicated as yellow, but yellow is a flat line.
Correct this plot.

3) Fig. 2B; I suggest placing the FOXA2 bar on top of all the other in the "% positive cells" plot. FOXA2-only cells appear in the most external portion of the colony. Putting the bar on top would reflect this spatial organization also in the plot. Easier to read.

4) Line 192-193 "FOXA2-only cells at the periphery suggesting that some of these cells were on their way to form endoderm (Fig 2B and Sup Fig 3)" and Fig. 2B
It seems that the FOXA2/TBXT cells are not giving rise directly to FOXA2 positive cells at the periphery. FOXA2/TBXT cells are located at intermediate radial position at time point 24h and 36h and the more peripheral cells are negative for both FOXA2 and TBXT at that time. In this peripheral position then we see the expression of FOXA2 at later time points (48h, 60hr). There is no time-point in which the cells at the periphery lose expression of TBXT and only keep FOXA2. Are FOXA2/TBXT positive cells really the progenitors of the FOXA2 positive cells we see at the periphery at later time points? Please comment!

5) Line 222-223 "indicating that at least a fraction of the endoderm emerging in micropattern colonies may adopt an hypoblast identity."
This hypothesis could match with the fact that the peripheral SOX17 cells do not seem to come from cells that were previously co-expressing TBXT-FOXA2 in that same peripheral position (= they are not derivative of the embryonic mesendoderm/primitive streak) [see comment for figure 2b].

6) Line 328-329 "implies that other mechanisms are also at play and it will be interesting to elucidate this in the future."
Very interesting that SMAD2/3 activity is not following the temporal pattern of NODAL. In fig. 5e. NODAL peaks after SMAD2/3 is already out of the nucleus. This could be in line with activation of TGFb-SMAD2/3 inhibitory mechanisms (e.g. inhibitory SMADs expression). Could this type of cell-autonomous mechanisms be part of the dynamics observed by the authors. Maybe also different time-scale for the expression of different target genes (such as LEFTY2 and CER1) or differences in the degradation of the products (mRNA or protein) of these target genes could play a role in this picture. The author could add a comment on this to the text if they see it fit.

<u>Reviewer 2</u>

Advance summary and potential significance to field

This study sets out to probe signalling requirements for APS fates, node and NotoPs. While the treatments and findings are of interest, the current text has many reasoning jumps which make it difficult to follow - partially because of the many conditions and fates obtained which are only

© 2024. Published by The Company of Biologists under the terms of the Creative Commons Attribution License (https://creativecommons.org/licenses/by/4.0/).

casually discussed. Data displays lack clarity and are often confusing. I do not recommend publication of the current format but do encourage authors to clarify some analyses/ images and have a more focused message. Please see below specific comments.

Comments for the author

Major points:
- On Fig.1B the authors show the variablilty of their assay in one experiment - which is great. However, we observe some colonies have wide range of SOX2 and almost no endoderm whilst others have a large SOX17 ring. It would be great if quantifications would show this variability and for us to know whether this is a feature across experiments (is c=25 from a single experiment? I would rather have a smaller number of representative colonies across different experiments). Unsure what is the purpose of using a single-cell/ nuclei segmentation tool if we are only shown an average radial profile. MIP in Fig.1C and tiny side-view are not helping. Zoomed inset appears to show cells are positive for all markers! Is this also MIP? Single-plane across multiple z could be better.

Figure 2 is interesting as the authors dissect which genes might be underlying endoderm induction in the previous pattern. The conclusion is "NODAL and WNT signalling correlate with the loss of axial cell fates". However several inconsistencies can be found:

- the strong claim that TBXT+FOXA2+ is highest at 36h is odd since it is also very present at 48h in the quantifications, images at 48h don't seem to match quantification.

- TBXT+SOX2+ population is also claimed to be the highest at 36h coming from periphery at 24h. However these timepoints do not appear to differ that much and is unclear TBXT induction at 24h is indeed happening at the edge/periphery of the colony and not inside an inner circle that expands further inside.

- Not sure where to find WNT evidence for this 24/36h correlation with axial fates. How do the NODAL/LEFTY domains spatially and temporally match the TBXT/SOX2 or TBXT/FOXA2 domains?

Fig2B - presenting a 60h time-course on a single disc split in 6 portions is ambitious but more problematic is that variablility of the pattern in each time-point appears too high to make a link between populations e.g. SOX17 appears to be induced both inside and at the edge of the colonies at 36h but then restricted to just the edge. TBXT appears excluded from the most inner part of the patterns at 48h but not at 36 or 60h. These aren't discussed or linked with the signals presented in following Figures.

Elongation in Fig.3C is interesting but feels poorly characterized. Gastruloids show elongating pole characterized by TBXT+ cells whereas here, TBXT+ NMPs seem to be located in the bottom of the colonies' original circle. Please expand or do not include.

Figure 5 does not inspire much confidence. SMAD2/3 is nuclear in hESCs (e.g. Vallier et al (2005)) but this is not seen in the control panel of Figure 5E at 0h. LEFTY2 and CER1 expression is pointed to panels 5E and B which is wrong. Fig 5F and 5G are never mentioned in the text. More importantly, the NODAL dynamics shown do not correspond to SMAD2/3 readout which after 1-2uM CHIR appears to be remain nuclear in all the colony until 24/36h.

Until Figure 5 the paper deals with NODAL and endoderm differentiation but in Figure 6 we are, rather abruptly, told NODAL signalling is "inadequate for NotoPs emergence" and needs to tested via "inhibitors added either throughout differentiation or at 24h". This reads very adhoc as the only mention of NotoPs we had in results was that they aren't found in any of the experiments so far.

Finally, I feel the result that the result that TGFbeta inhibition potentiates WNT is quite important and would be great to dissect whether this is mainly from the NODAL or from the BMP branches given the previous finding that LDN resulted in an increase of NODAL.

Other points:

Fig1A is very confusing. Unclear about the orientation of PS in saggital view.

© 2024. Published by The Company of Biologists under the terms of the Creative Commons Attribution License (https://creativecommons.org/licenses/by/4.0/).

Appears rotated and in either case wrong (e.g. is this at midline? Where is the paraxial mesoderm/ notochord in rostral side?). Dorsal view appears almost as a prospective fate map but this is not made clear, from the description:"putative organisation and hierarchies of cell fates". What is the reference for this organisation in human?

SOX1 would be better neural as PAX6 could also reflect a more dorsal or anterior domain.

TBXT/SOX2 or TBXT+/SOX2+ is confusing. Either TBXT+SOX2+ or another format to denote double positives not the the forward dash, commonly be used to state alternatives.

Fig2A - colours of genes/ pathways is very difficult to follow. E.g. 3 shades of red... Wnts appear pink but label shows red/peach. Also some confusing assignments, e.g. MESP1 as a LPM marker?

Rescue is an odd choice of word in "Nodal inhibition rescues NMP emergence" as the authors show double-positive cells have emerged before.

The lack of a connecting thread between conditions make the study hard to follow.

In Fig.3 we are now presented with a 3 day protocol. What was the rationale for this ? In Fig.1 the authors chose a 2 days protocol when in the reference they provide of Frith and Tsakiridis(2019) was 3.

The colonies do not appear attached in Fig 3. Is this significant? Fixation artifact?

First revision

Author response to reviewers' comments

Reviewer 1 Comments for the Author:

The authors have made substantial improvement to the work and I see it now suitable for publication. Below I have some comments and suggestions that the authors can address at their discretion. These points will not affect the main messages of the authors' manuscript:

We are grateful for the positive and helpful comments. We provide responses to individual points below.

1) Line 102 “SOX2-only domain in the center and a SOX17 domain at the periphery (Fig. 1B and 1C)” From the pictures the cells seem to be almost all SOX17/TBXT positive. Maybe reflect this in the text.

This is a good point. In fact, we see a mixture of SOX2-only cells and SOX2+TBXT+ cells in the center. We changed our wording L98 “SOX2-only cells were found in the center and at the top of the colony while SOX2+TBXT+ cells were most abundant around 100μm from the colony border”

2) Fig. 1E; I see 6 curves/lines in the plot. Is the brown line for FOXA/SOX17? In the legend is indicated as yellow, but yellow is a flat line. Correct this plot.

Thank you for spotting this issue. We corrected the plot in Fig 1E

3) Fig. 2B; I suggest placing the FOXA2 bar on top of all the other in the "% positive cells" plot. FOXA2-only cells appear in the most external portion of the colony. Putting the bar on top would reflect this spatial organization also in the plot. Easier to read.

© 2024. Published by The Company of Biologists under the terms of the Creative Commons Attribution License (https://creativecommons.org/licenses/by/4.0/).

We have improved Fig 2B (Fig 2 B-H) to address points 4 and 5 of this reviewer and points 4-7 from reviewer 2. We have considered reformatting the stacked bar plots as suggested but the different cell populations overlap in space. This makes it difficult to accurately represent the positioning of the different cell populations on the stacked bar plot. Instead, we provide clearer radial profiles in Fig 2 D and 2G using the same color code as in the bar plot. We hope this will be satisfactory to this reviewer.

4) Line 192-193 “FOXA2-only cells at the periphery suggesting that some of these cells were on their way to form endoderm (Fig 2B and Sup Fig 3)” and Fig. 2B

It seems that the FOXA2/TBXT cells are not giving rise directly to FOXA2 positive cells at the periphery. FOXA2/TBXT cells are located at intermediate radial position at time point 24h and 36h and the more peripheral cells are negative for both FOXA2 and TBXT at that time. In this peripheral position then we see the expression of FOXA2 at later time points (48h, 60hr). There is no time-point in which the cells at the periphery lose expression of TBXT and only keep FOXA2. Are FOXA2/TBXT positive cells really the progenitors of the FOXA2 positive cells we see at the periphery at later time points? Please comment!

We agree that this is an important point. As mentioned above, we have modified Fig2B and decided to repeat our time course experiment in order to add SOX17 to our FOXA2 and TBXT co-staining. The results are shown in Fig 2E-G.
We observe that the SOX17+ cells which first appear at 36h are also positive for TBXT and FOXA2 (See Fig 2E, zoomed insets and F). At 48h, the number of FOXA2+TBXT+ decreases while the number of triple+ cells increases. We also observe FOXA2+SOX17+ (TBXT-) appearing at 48h for the first time. This trend is confirmed at 60h with an even higher proportion of FOXA2+SOX17+ and fewer of the FOXA2+TBXT+ or Triple+ populations.
We think that these observations fit the idea that FOXA2+TBXT+ cells are the progenitors of at least some of the FOXA2+SOX17+ cells at 48h and 60h, although we cannot conclude that this is the case without a reporter line. We updated our text L185-191

5) Line 222-223 “indicating that at least a fraction of the endoderm emerging in micropattern colonies may adopt an hypoblast identity.”

This hypothesis could match with the fact that the peripheral SOX17 cells do not seem to come from cells that were previously co-expressing TBXT-FOXA2 in that same peripheral position (= they are not derivative of the embryonic mesendoderm/primitive streak) [see comment for figure 2b].

This is another interesting point, thank you. In our new Fig 2E-G, we do note the presence of cells positive for FOXA2 only. These cells emerge at 24h around the central region and are then progressively shifted towards a domain located around 50µm from the border of the colony (Fig 2G). We are unsure about their identity, but these cells may contribute to forming DE or hypoblast-like cells in the colonies. We indeed observe signs of hypoblast identity in our cultures (Sup Fig. 2A and Sup.
Fig. 4C). This observation would fit recent reports in mice and pig embryos of DE/hypoblast fated FOXA2+TBXT- cells emerging anterior from the FOXA2+TBXT+ domain (Simpsons 2024, Scheibner 2021) We commented on this observation in the discussion L486-491.

6) Line 328-329 “implies that other mechanisms are also at play and it will be interesting to elucidate this in the future.”

Very interesting that SMAD2/3 activity is not following the temporal pattern of NODAL. In fig. 5e. NODAL peaks after SMAD2/3 is already out of the nucleus. This could be in line with activation of TGFb-SMAD2/3 inhibitory mechanisms (e.g. inhibitory SMADs expression). Could this type of cell- autonomous mechanisms be part of the dynamics observed by the authors. Maybe also different time- scale for the expression of different target genes (such as LEFTY2 and CER1) or differences in the degradation of the products (mRNA or protein) of these target genes could play a role in this picture. The author could add a comment on this to the text if

© 2024. Published by The Company of Biologists under the terms of the Creative Commons Attribution License (https://creativecommons.org/licenses/by/4.0/).

they see it fit.

Thank you for the suggestion, we added a sentence in the result section to further highlight these observations: L310: “Somewhat consistently with the spatio-temporal dynamics of NODAL expression, some cells with nuclear SMAD2/3 persisted until 36h at 2μM CHIR and until 48h at the periphery of 1μM treated colonies (Fig. 5E). However, nuclear SMAD2/3 did not strictly follow NODAL expression. For example, nuclear SMAD2/3 increased sharply as early as 6h post-CHIR treatment - before the peak of NODAL expression (Fig. 5C-E) - and was heterogeneous inside the domain of NODAL expression (Fig. 5D-E).”

Reviewer 2 Advance Summary and Potential Significance to Field:

This study sets out to probe signalling requirements for APS fates, node and NotoPs. While the treatments and findings are of interest, the current text has many reasoning jumps which make it difficult to follow - partially because of the many conditions and fates obtained which are only casually discussed. Data displays lack clarity and are often confusing. I do not recommend publication of the current format but do encourage authors to clarify some analyses/ images and have a more focused message. Please see below specific comments.

We thank this reviewer for their helpful constructive criticism and interest in our work. We address each point below and agree that this helped improve our manuscript.

Reviewer 2 Comments for the Author:

Major points:

1) On Fig.1B the authors show the variablilty of their assay in one experiment - which is great. However, we observe some colonies have wide range of SOX2 and almost no endoderm whilst others have a large SOX17 ring. It would be great if quantifications would show this variability and for us to know whether this is a feature across experiments (is c=25 from a single experiment? I would rather have a smaller number of representative colonies across different experiments).

Thank you for this comment; we added the number of times each experiment was repeated in all the legends of the main figures.
Regarding Fig 1B, we observe this level of variability (or we’d like to say “reproducibility”) across all our experiments. We think this variability comes from differences in seeding density across the well at 0h. We commented on this in the figure legend.
Fig 1B is a representative experiment from at least 6 independent experiments (see also the 48h time point in Fig 2B, the control conditions in Fig 3A and 6D or the 2μM condition in Fig 4A and Sup Fig 6).
To better document variability across experiments, we include radial profiles for several of these experiments across the manuscript. Please note that the shaded area around the lines in the quantifications indicates the 95% confidence interval (calculated across all the colonies of the same well) and is an accurate representation of the variability.

2) Unsure what is the purpose of using a single-cell/ nuclei segmentation tool if we are only shown an average radial profile.

Please see our comment to the previous point – Single cell quantification allows us to determine the percentage of cell types in each bin across the radial distance. This would not be possible without single cell resolution.

3) MIP (max intensity projection) in Fig.1C and tiny side-view are not helping. Zoomed inset appears to show cells are positive for all markers! Is this also MIP? Single-plane across multiple z could be better.

© 2024. Published by The Company of Biologists under the terms of the Creative Commons Attribution License (https://creativecommons.org/licenses/by/4.0/).

Thank you for the suggestion. We have kept the max intensity projection in the main figure but replaced the zoomed insets with single planes (explained in the legend). We agree that this now better shows the diversity of marker expression profiles. We also provide a new supplementary figure 1 showing single planes across the depth of the colonies shown in Fig 1 C and Supplementary fig 3 showing single planes across the depth of the colony shown in Fig 1 E.

4) Figure 2 is interesting as the authors dissect which genes might be underlying endoderm induction in the previous pattern. The conclusion is "NODAL and WNT signalling correlate with the loss of axial cell fates". However several inconsistencies can be found:

the strong claim that TBXT+FOXA2+ is highest at 36h is odd since it is also very present at 48h in the quantifications, images at 48h don't seem to match quantification.

Our main point here was that the percentage of TBXT+FOXA2+ (the double positive population) is highest at 36h and then decreases to the benefit of a FOXA2+ but TBXT- population. This could be seen in the bar chart of the original Fig 2B provided below for reference.

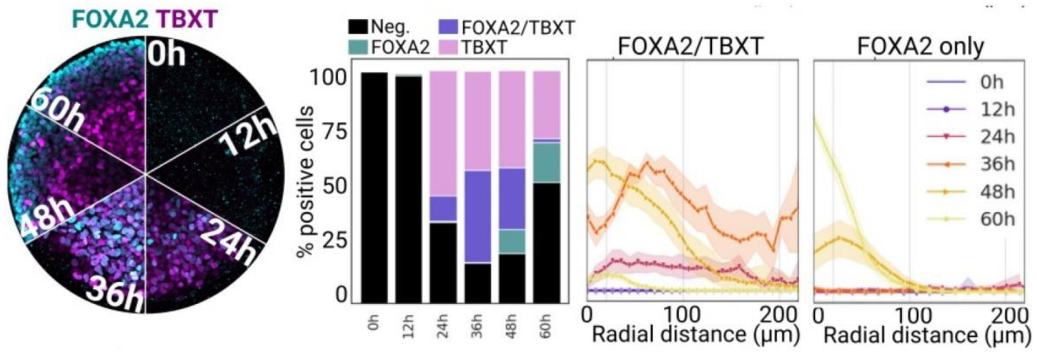

However, we agree that this point needed clarification. We now provide a revised Fig 2B where we replaced the original disc splits with 4-quadrants montages showing each individual channel and time point separately as well as single-plane zoomed insets. We also provide radial profiles of all populations for every time point and decided to repeat our time course experiment in order to add SOX17 to our FOXA2 and TBXT co-staining.

We clarify our observations in the main text L185: “While TBXT+SOX2+ cells (Fig 2B-D) and FOXA2+TBXT+ cells (Fig 2 E-G) appeared at 24h at the periphery of the colonies and increased in proportion until 36h, these populations were progressively lost at later time points in favour of cells expressing other marker combinations. Notably, we observed the emergence of SOX2+TBXT+SOX17+ and FOXA2+TBXT+SOX17+ triple positive cells at 36h indicating that TBXT+SOX2+ and FOXA2+TBXT+ cells found before 36h are neither NMPs nor Notochord but are instead likely to represent the precursors of the mesoderm and endoderm”

5) TBXT+SOX2+ population is also claimed to be the highest at 36h coming from periphery at 24h. However these timepoints do not appear to differ that much and is unclear TBXT induction at 24h is indeed happening at the edge/periphery of the colony and not inside an inner circle that expands further inside.

We agree that this was unclear in the former version of the figure. We provide new images and quantifications in Fig. 2B-G. We can now clearly see that TBXT+SOX2+ (2C-D) and TBXT+FOXA2+ (2F-G) cells appear first at the periphery of the colony at 24h and that their positioning is progressively shifted inwards (2D and 2G)

6) Not sure where to find WNT evidence for this 24/36h correlation with axial fates.

© 2024. Published by The Company of Biologists under the terms of the Creative Commons Attribution License (https://creativecommons.org/licenses/by/4.0/).

We removed mention of WNT from the section heading - now “Dynamics of NODAL signaling correlates with the loss of axial cell fates and the emergence of endoderm and mesoderm”

7) How do the NODAL/LEFTY domains spatially and temporally match the TBXT/SOX2 or TBXT/FOXA2 domains?

Fig2B - presenting a 60h time-course on a single disc split in 6 portions is ambitious but more problematic is that variablility of the pattern in each time-point appears too high to make a link between populations e.g. SOX17 appears to be induced both inside and at the edge of the colonies at 36h but then restricted to just the edge. TBXT appears excluded from the most inner part of the patterns at 48h but not at 36 or 60h.

These aren't discussed or linked with the signals presented in following Figures.

We agree that these are important points and that they were not sufficiently well explained in the previous version of the manuscript. As mentioned in our response to point 4) we now provide an improved Fig 2B-H which includes radial profiles for every time point and for all marker combinations. This enables us to draw clearer conclusions. We revised our Fig 8 to clarify our message and added the following comments in the main text:

L304 : “the spatio-temporal pattern of NODAL expression in 2µM colonies correlated nicely with the pattern of FOXA2 and TBXT expression found earlier (Fig 2 E-G), further reinforcing the notion that endogenous NODAL dictates cell fate patterning in micropatterned colonies”

L324: “Together, our results demonstrate that small variations in CHIR concentration induce distinct spatio-temporal dynamics of endogenous NODAL signalling in micropatterned colonies. At high CHIR concentration (3 and 4µM), NODAL expression is rapidly extinguished and a strong WNT signalling activity induces mesodermal TBXT single positive cells across the entire colony. On the other hand, 1 and 2µM CHIR treatment induce low to moderate levels of LEF1 and an inward wave of NODAL expression initiated from the boundary which persists until 48h. The existence of such a wave, indicates that cells located at the periphery experience an earlier, and therefore prolonged, exposure to NODAL signalling compared to the cells at the centre. This spatio-temporal pattern correlates with the positioning of FOXA2+TBXT+ cells at 24h and the later emergence of endodermal SOX17+ cells in these colonies (Fig. 2E-G, Fig. 4A). SOX17 expression is likely the result of continuous exposure of FOXA2+TBXT+ cells to NODAL after 24h.”

L451:” existence of the NODAL wave means that the cells are subjected to distinct timing and duration of NODAL depending on their position along the colony radius (Fig 8A). In particular, early and sustained production of NODAL at the periphery (Fig 5D) defined a domain of endodermal differentiation marked by SOX17 expression (Fig. 1B, 2B-H and 4)”

L461: “while SOX17+ cells emerged preferentially in a domain overlapping the 24h NODAL-expressing domain (Fig 5D), they did so in a scattered manner at 36h before regrouping together at the periphery (Fig 2B and E). This apparent stochastic emergence followed by a sorting behaviour is reminiscent of observations in mouse ESC colonies (Pour et al., 2022), human gastruloids (Moris et al., 2020; Vianello and Lutolf, 2020) and embryos (Burtscher and Lickert, 2009; Scheibner et al., 2021; Simpson et al., 2024) and It will be interesting to determine the mechanisms ensuring the robust outcome of this process.”

8) Elongation in Fig.3C is interesting but feels poorly characterized. Gastruloids show elongating pole characterized by TBXT+ cells whereas here, TBXT+ NMPs seem to be located in the bottom of the colonies' original circle. Please expand or do not include.

We decided to keep this figure because we think it reinforces our claim that elongation-competent NMPs emerge in our colonies.

We specify in the legend that images provided in Fig 3C are representative of 3 independent experiments and now provide a new Supplementary Fig 5. showing a quantification of the

© 2024. Published by The Company of Biologists under the terms of the Creative Commons Attribution License (https://creativecommons.org/licenses/by/4.0/).

percentage of elongating colonies in each condition.

We also clarified in the text that NMP-like cells were indeed found at the bottom of the colony. L249: “Interestingly, SOX2+TBXT+ cells were located where the cells initially touched the pattern (Fig 3C). The predictable location of TBXT-expressing cells in this system is consistent with previous work showing that cell-substrate contact dictates symmetry breaking in 3D aggregates (Sagy et al., 2019).”

9) Figure 5 does not inspire much confidence. SMAD2/3 is nuclear in hESCs (e.g. Vallier et al (2005)) but this is not seen in the control panel of Figure 5E at 0h.

We do not think that the conditions used in Vallier 2005 and ours are directly comparable. Vallier et al used a distinct medium composition: KO-DMEM + 20% Serum replacer and grew the cells on MEF feeder layers at a low density. In our 0h time point, the cells are in mTESR plus medium and confined on micropatterns. Cell density has been shown to modulate the subcellular localisation of SMAD2/3 – see for example fig1 of Varelas et al 2001 (https://doi.org/10.1016/j.devcel.2010.11.012).

These differences in growing culture conditions and in particular cell density probably explain our results. We have reproduced this result at least 3 times and are confident that only very low SMAD2/3 expression can be detected in pluripotent hESC on micropatterns at the beginning of our experiments.

10) LEFTY2 and CER1 expression is pointed to panels 5E and B which is wrong. Fig 5F and 5G are never mentioned in the text.

Thank you, this has been corrected in the text (L319 and 320)

11) More importantly, the NODAL dynamics shown do not correspond to SMAD2/3 readout which after 1-2uM CHIR appears to be remain nuclear in all the colony until 24/36h.

The fact that nuclear SMAD23 does not strictly follow NODAL expression is correct and we commented on this observation in our text L310: “Somewhat consistently with the spatio-temporal dynamics of NODAL expression, some cells with nuclear SMAD2/3 persisted until 36h at 2μM CHIR and until 48h at the periphery of 1μM treated colonies (Fig. 5E). However, nuclear SMAD2/3 did not strictly follow NODAL expression. For example nuclear SMAD2/3 increased sharply as early as 6h post-CHIR treatment - before the peak of NODAL expression (Fig. 5C-E) - and was heterogeneous inside the domain of NODAL expression (Fig. 5D-E).”

We are however confident that our SMAD2/3 can be trusted as we observe the exclusion of SMAD2/3 from the nuclei of the cells upon SB treatment (See Sup fig 11)

12) Until Figure 5 the paper deals with NODAL and endoderm differentiation but in Figure 6 we are, rather abruptly, told NODAL signalling is "inadequate for NotoPs emergence" and needs to tested via "inhibitors added either throughout differentiation or at 24h". This reads very adhoc as the only mention of NotoPs we had in results was that they aren't found in any of the experiments so far.

We concede that our narrative transition between section 5 and 6 in the text needed clarification. We rewrote the last paragraph of section 5 (L324 -336) and explained the rationale that led us to test NODAL inhibition at 24h: “SOX17 expression is likely the result of continuous exposure of FOXA2+TBXT+ cells to NODAL after 24h. These observations raise the possibility that redirecting FOXA2+TBXT+ cells towards the notochord could be achieved if continuous exposure to NODAL is prevented”.

We would like to mention that our narrative follows quite closely our journey to find the

© 2024. Published by The Company of Biologists under the terms of the Creative Commons Attribution License (https://creativecommons.org/licenses/by/4.0/).

conditions for NotoPs emergence on micropatterns and highlights a role for serendipity in revealing how combinatorial WNT and NODAL singalling distinguish the notochord fate from other related cell fates (Summarised in Fig 8). We mentioned NotoPs from the very beginning of our result section (L86) and explain why we chose to test specific conditions in relation to our pursuit of NotoPs – Please see in order L158-162, L218-221, L253-254, L334-336, L340-341. We hope this reviewer will agree that our article forms a well rounded story in spite of the many tested conditions.

14) Finally, I feel the result that TGFbeta inhibition potentiates WNT is quite important and would be great to dissect whether this is mainly from the NODAL or from the BMP branches given the previous finding that LDN resulted in an increase of NODAL.

This is a very good point. We have expanded Fig7 and now include LEF1 staining over time for all conditions tested in Fig6. Our results clearly show that NODAL rather than BMP signalling inhibition potentiates WNT signalling.

We also included a SMAD2/3 staining in Sup fig 11 showing how SB treatment results in SMAD2/3 exclusion from the nucleus, further reinforcing confidence in our SMAD23 staining in Fig 5

Other points:

Fig1A is very confusing. Unclear about the orientation of PS in saggital view.
Appears rotated and in either case wrong (e.g. is this at midline? Where is the paraxial mesoderm/ notochord in rostral side?). Dorsal view appears almost as a prospective fate map but this is not made clear, from the description:"putative organisation and hierarchies of cell fates". What is the reference for this organisation in human?

Thank you for the useful feedback. We modified this diagram to make it clearer. For example, we corrected the positioning of NotoPs (in green) so they only appear on the ventral side of the node (based on lineage tracing in mice – see Cambray and Wilson 2002). We also outlined the node with a dashed blue line and indicated the positioning of the PS in the sagittal view to clarify the orientation of the sagittal view.

* The sagittal view indeed shows the domain surrounding the node along the midline. This is now mentioned in the legend of the figure.
* This diagram represents the embryo at the end of primary gastrulation and just prior axial elongation - before the notochord starts emerging from the node. For this reason, we did not represent notochord nor paraxial mesoderm on the rostral side. We also removed the blue color from the anterior epiblast which initially indicated the neurectoderm fate since, at this stage, the anterior epiblast is still pluripotent.
* To construct this diagram, we used references from mouse work as indicated in the text and also referred to observations made in CS7 human embryos: Tyser et al 2021, Bakker et al. 2016 and De Bree 2018.

SOX1 would be better neural as PAX6 could also reflect a more dorsal or anterior domain.

We agree, this is a good suggestion, but we have not explored SOX1 expression in our colonies because we could not find a reliable SOX1 antibody. We also removed mention of PAX6.

TBXT/SOX2 or TBXT+/SOX2+ is confusing. Either TBXT+SOX2+ or another format to denote double positives not the the forward dash, commonly be used to state alternatives.

Thank you for the suggestion, we changed our notation throughout the text.

Fig2A - colours of genes/ pathways is very difficult to follow. E.g. 3 shades of red... Wnts appear pink but label shows red/peach. Also some confusing assignments, e.g. MESP1 as a LPM marker?

We agree. To reduce the number of colours used in this figure, we kept a colour scheme for

© 2024. Published by The Company of Biologists under the terms of the Creative Commons Attribution License (https://creativecommons.org/licenses/by/4.0/).

signalling pathways but used a code made of symbols for fate markers. We also revised our assignments to (cautiously) raise the hypothesis that hypoblast / Yolk sac like cells as well as extraembryonic mesoderm may be specified in our colonies (L199-206).
Regarding MESP1: Mesp1-Cre lineage tracing experiments in mice have shown that MESP1 labels Extraembryonic mesoderm and anterior mesoderm including lateral plate mesoderm (Saga et al., 1999; Lescroart et al., 2018; Probst 2021).

Rescue is an odd choice of word in "Nodal inhibition rescues NMP emergence" as the authors show double-positive cells have emerged before.

Fig 2B-G is useful to clarify this point. The domain of SOX2+TBXT+ in 2B-D, overlaps with the TBXT+FOXA2+ domain in fig 2E-G. We infer that the SOX2+TBXT+ population is in fact also positive for FOXA2 (unfortunately we were not able to perform a SOX2, TBXT, FOXA2 co-staining due to antibody incompatibility). NMPs never express FOXA2 and we think that SOX2+TBXT+ cells in this context are not NMPs but precursors of mesoderm and endoderm. We clarify this in the text line 193-194.
We do not exclude that some NMPs are specified in the control with 2μM CHIR but these are a minority of the cells and are not maintained at 60h, i.e they differentiate into mesodermal TBXT+ only cells.
Nevertheless, to address this point, we rephrased our heading to "Constant NODAL inhibition safeguards NMPs but fails to drive NotoPs"

The lack of a connecting thread between conditions make the study hard to follow. In Fig.3 we are now presented with a 3 day protocol. What was the rationale for this ?

In the previous figure (Fig 2) we showed that TBXT+SOX2+ cells were progressively lost and absent at 60h (day 2.5). In Fig.3, we wanted to test whether NODAL inhibition can prevent the loss of NMPs. We therefore chose to fix and analyse the cells at day 3, a stage when SOX2+TBXT+ cells are definitively lost in the control. We show in Fig 3. that NODAL inhibition does indeed prevent this loss.

To clarify this in the text we added the following sentence L 244: "We showed earlier that SOX2+TBXT+ cells were almost completely lost without SB treatment (Fig 2C). We thus checked whether SOX2+TBXT+ NMPs were still present in colonies with NODAL inhibition at day 3 and 6."

In Fig.1 the authors chose a 2 days protocol when in the reference they provide of Frith and Tsakiridis(2019) was 3.

This is correct, however SOX2+TBXT+ cells were also reported as early as day2 of CHIR/FGF treatment (Fig 5B Gouti et al 2014). We decided to focus on the 48h time point in our system when the colonies are still thin enough and easy to image and quantify.

The colonies do not appear attached in Fig 3. Is this significant? Fixation artifact?

Some colonies became loosely attached during the staining procedure, this is visible in Fig3 as noted by this reviewer. We comment on this in the legend of Fig 3 and Sup Fig 1

<u>Second decision letter</u>

MS ID#: DEVELOP/2024/202983

MS TITLE: In vitro modelling of anterior primitive streak patterning with human pluripotent stem cells identifies the path to notochord progenitors

AUTHORS: Miguel Robles-Garcia, Chloe Thimonier, Konstantina Angoura, Ewa Ozga, Heather MacPherson, and Guillaume Blin

© 2024. Published by The Company of Biologists under the terms of the Creative Commons Attribution License (https://creativecommons.org/licenses/by/4.0/).

I have now received all the referees reports on the above manuscript, and have reached a decision. The referees' comments are appended below, or you can access them online: please go to BenchPress and click on the 'Manuscripts with Decisions' queue in the Author Area.

The overall evaluation is positive and we would like to publish a revised manuscript in Development, provided that the referees' comments can be satisfactorily addressed. Please attend to all of the reviewers' comments in your revised manuscript and detail them in your point-by-point response. If you do not agree with any of their criticisms or suggestions explain clearly why this is so. If it would be helpful, you are welcome to contact us to discuss your revision in greater detail. Please send us a point-by-point response indicating your plans for addressing the referees' comments, and we will look over this and provide further guidance.

Reviewer 1

Advance summary and potential significance to field

-

Comments for the author

I am in favor of the publication of the manuscript.
Congrats to the authors for all their efforts!

Reviewer 2

Advance summary and potential significance to field

The authors have clarified many points and overall improved the manuscript.
Supplementary Figures 1 and 2 are make their main treatment more clear and highlight its reproducibility across cell lines. However, I still maintain that some concepts are mentioned carelessly (see below) and the number of different treatments appear disjoint making the manuscript difficult to follow. The main issues that must be addressed before I can recommend publication are:

Comments for the author

1) As mentioned before, the connection to the embryo is still not clear. In particular with regards to the rostrocaudal position. The diagram presented in Fig.1A denotes an early, full primitive streak stage embryo, which fits with an exploration of APS. However, the first sentence of the Results section states "NotoPs emerge near NMPs within a small, confined region of the embryo (Cambray and Wilson, 2007).". The NMPs are a posterior, Cdx2+, population that emerges later in development and indeed the citation refers to an older, E8.5 embryo with already a considerable number of somites (not reflecting APS or Fig 1A).

This misconception is then carried throughout the text with statements such as "NMP derivation medium ...could elicit the self-organisation of hESC into distinct domains of APS" or that indeed CDX2 is associated with an anterior, APS marker (Figure2), whilst in NMPs stages CDX2 is a clear marker of posterior progenitor populations.

2) Although this is clarified later in the text, the title of Fig2 as it stands from the begining of the text, doesn't match the evidence. "Dynamics of NODAL signalling correlate with the loss of axial cell fates and the emergence of mesoderm and endoderm". Endoderm starts spotty at the edge at 36h (Fig2B) and NODAL expression starts at 12h and peaks at 24h. If anything, emergence of endoderm "correlates" with OTX2 and cluster 3 and 6.
3) SMAD2/3 stainings and quantifications are still not very convincing. I would suggest looking at nucler/cytoplasm ratio as it is common in the field. It is true that Vallier 2005 conditions aren't compatible but that is also the case for the reference given which uses other cell lines. I suggest citing https://www.nature.com/articles/ncb1748 using H9s as well as showing in supplement a

© 2024. Published by The Company of Biologists under the terms of the Creative Commons Attribution License (https://creativecommons.org/licenses/by/4.0/).

more clear picture of this density effect at ESC stage - which should be straightforward as the experiment was reproduced already 3 times.

Below I have some comments and suggestions that the authors can address at their own discretion.

1) Following my comment regarding single-cell segmentation. Percentage of cell types across radial distance is great but it ignores z-plane. As Supp.Fig.1A induction appears to show, SOX17 is actually quite wide-spread at the bottom of the colonies. This might be missed in a radial plot as the absolute number of cells varies throughtout the colony (in this case, more cells at the center of the dome).

2) "MESP1 labels Extraembryonic mesoderm and anterior mesoderm including lateral plate mesoderm". As stated, this makes MESP1 a pan-marker of anterior mesoderm and not a marker for LPM.

3) I would also change the TBXT/SOX2 nomenclature in Figures to match the text.

Second revision

Author response to reviewers' comments

Reviewer 1 Comments for the Author:
I am in favor of the publication of the manuscript.
Congrats to the authors for all their efforts!

Many thanks, this is much appreciated!

Reviewer 2 Advance Summary and Potential Significance to Field:
The authors have clarified many points and overall improved the manuscript. Supplementary Figures 1 and 2 are make their main treatment more clear and highlight its reproducibility across cell lines.
However, I still maintain that some concepts are mentioned carelessly (see below) and the number of different treatments appear disjoint making the manuscript difficult to follow. The main issues that must be addressed before I can recommend publication are:

We appreciate the reviewers constructive comments. We provide a response to individual points below and highlighted our changes in yellow in the manuscript. We hope that our revisions clarify the last remaining issues.

Reviewer 2 Comments for the Author:

1) As mentioned before, the connection to the embryo is still not clear. In particular with regards to the rostrocaudal position. The diagram presented in Fig.1A denotes an early, full primitive streak stage embryo, which fits with an exploration of APS. However, the first sentence of the Results section states "NotoPs emerge near NMPs within a small, confined region of the embryo (Cambray and Wilson, 2007).". The NMPs are a posterior, Cdx2+, population that emerges later in development and indeed the citation refers to an older, E8.5 embryo with already a considerable number of somites (not reflecting APS or Fig 1A).

We thank the reviewer for further explaining the source of the confusion in our manuscript. To clarify our thinking, we now cite a more appropriate reference: Forlani et al 2003 (L56 and 82). Please see the lineage tracing performed by these authors in Fig 7, in particular the blue empty circle and brown circles (NM fated and notochord fated cells respectively). The results show that Notochord and NM- fated cells can be found as early as the late streak stage in the mouse embryo just adjacent to the node. Using retrospective clonal analysis in mice, others have also shown that NM-fated cells emerge around mid-gastrulation and before the onset of axial elongation

© 2024. Published by The Company of Biologists under the terms of the Creative Commons Attribution License (https://creativecommons.org/licenses/by/4.0/).

(Tzouanacou et al 2009, Fig 3). Furthermore, cells with a neuro-mesoderm competent phenotype (Sox2+Tbxt+) are present at late bud to early head fold stage (Dias et al 2020; Wymeersch 2016).

We rephrased our sentence: "*In mice, NotoPs emerge near NM-fated cells during gastrulation within a small, confined region of the embryo*", and revised our nomenclature throughout the manuscript (highlighted in yellow). We hope this reviewer will agree that these revisions clarify the link we make with the embryo and that our claims are well-supported by the above references.

This misconception is then carried throughout the text with statements such as "NMP derivation medium ...could elicit the self-organisation of hESC into distinct domains of APS" or that indeed CDX2 is associated with an anterior, APS marker (Figure2), whilst in NMPs stages CDX2 is a clear marker of posterior progenitor populations.

We agree that CDX2 is a posterior marker. We added the correct symbols in Fig 2 to associate CDX2 with NM and mesoderm. We would like to clarify that what we call the APS is still part of the primitive streak and is localised on the posterior side of the embryo relative to the node - We added APS and PPS (posterior primitive streak) labels on our diagram in Fig 1A to help the reader understand this point. We also note that in early bud stage mouse embryos, prior to axial elongation, CDX2 labels the entire streak, including the APS (see for example fig.1 of F. Beck https://doi.org/10.1042/BST0380353). This justifies why we maintained the APS symbol next to the CDX2 gene.

2) Although this is clarified later in the text, the title of Fig2 as it stands from the begining of the text, doesn't match the evidence. "Dynamics of NODAL signalling correlate with the loss of axial cell fates and the emergence of mesoderm and endoderm". Endoderm starts spotty at the edge at 36h (Fig2B) and NODAL expression starts at 12h and peaks at 24h. If anything, emergence of endoderm "correlates" with OTX2 and cluster 3 and 6.

We changed the title of Fig 2 to be more accurate: "NODAL signalling precedes the loss of axial cell fate markers and the emergence of mesoderm and endoderm"

3) SMAD2/3 stainings and quantifications are still not very convincing. I would suggest looking at nucler/cytoplasm ratio as it is common in the field. It is true that Vallier 2005 conditions aren't compatible but that is also the case for the reference given which uses other cell lines. I suggest citing https://www.nature.com/articles/ncb1748 using H9s as well as showing in supplement a more clear picture of this density effect at ESC stage - which should be straightforward as the experiment was reproduced already 3 times.

We agree that quantifying the nuclear/cytoplasm ratio would be ideal. Unfortunately, the high cell density on micropatterns makes it near impossible to accurately segment the cytoplasm of the cells on micropatterns - even manually. The average nuclear intensity is the best we could report here and we think this gives a good indication of the relative levels of active SMAD2/3 signalling per micropattern. We do not make any strong claim in the manuscript about the dynamics of nuclear SMAD2/3 - only that SMAD2/3 levels are largely consistent with NODAL expression given that cells with nuclear SMAD2/3 persisted for a longer period of time at 1 and 2µM CHIR concentrations when endogeneous NODAL expression lasts the longest (see L309-315). Given these considerations, we hope this reviewer will be satisfied with the current quantifications.

Nevertheless, to convince this reviewer about the validity of our SMAD2/3 staining, we added new images in supplementary figure 11 (D) with high resolution insets to show the specificity of our SMAD2/3 signal (reproduced below). Notice how SMAD2/3 is found nuclear at the periphery of the colonies with 2µM CHIR but remains cytoplasmic at the centre of the same colony or in the colony treated with 10µM SB as expected.

© 2024. Published by The Company of Biologists under the terms of the Creative Commons Attribution License (https://creativecommons.org/licenses/by/4.0/).

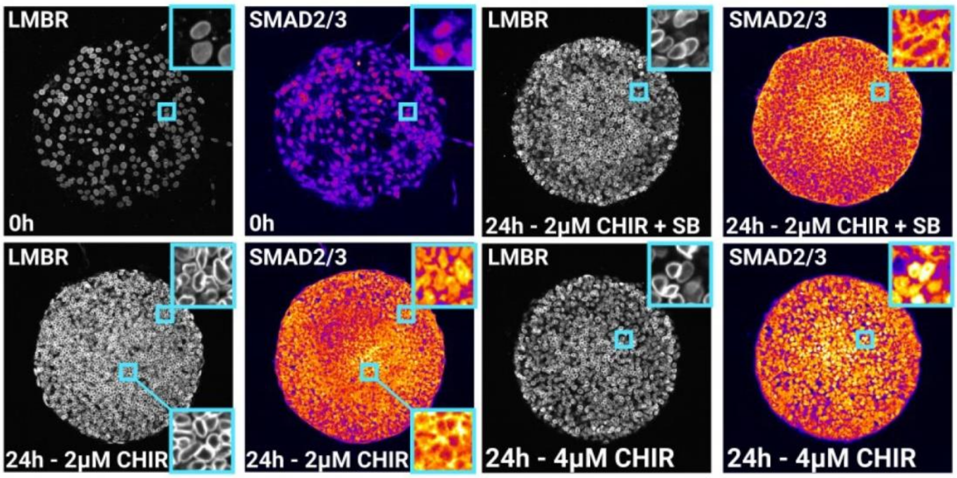

As discussed with this reviewer, we also think that there is an effect of cell density at time 0h on the level of nuclear SMAD2/3, in agreement with previous reports. We provide an example of this observation below. We included the suggested reference line 315.

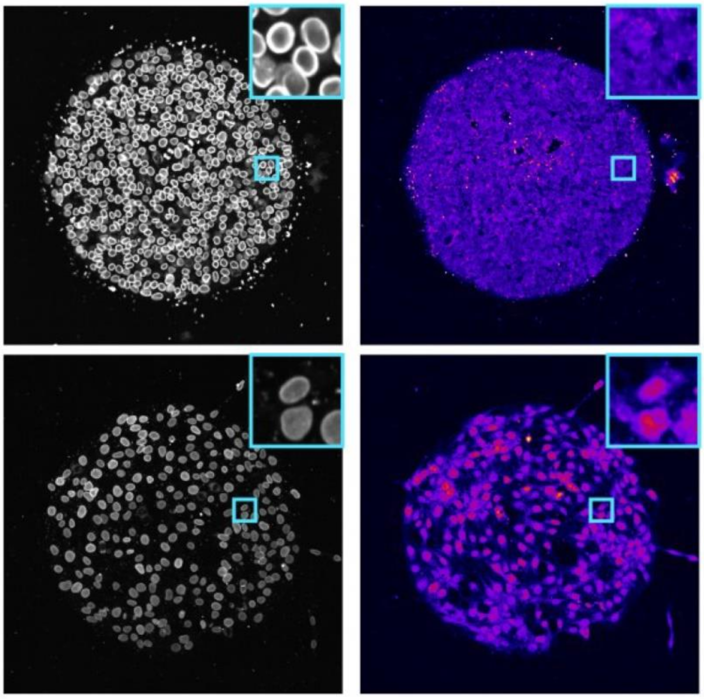

Below I have some comments and suggestions that the authors can address at their own discretion.

1) Following my comment regarding single-cell segmentation. Percentage of cell types across radial distance is great but it ignores z-plane. As Supp.Fig.1A induction appears to show, SOX17 is actually quite wide-spread at the bottom of the colonies. This might be missed in a radial plot as the absolute number of cells varies throughtout the colony (in this case, more cells at the center of the dome).

We agree with the reviewer, we incorporated a new plot in supplementary figure 1A to show the average Z positioning of individual population along the radial distance from the colony edge. We observe that SOX2+ cells are localised at the top of colony while SOX17+ cells

2) "MESP1 labels Extraembryonic mesoderm and anterior mesoderm including lateral plate mesoderm". As stated, this makes MESP1 a pan-marker of anterior mesoderm and not a marker for LPM.

© 2024. Published by The Company of Biologists under the terms of the Creative Commons Attribution License (https://creativecommons.org/licenses/by/4.0/).

We changed our text to “anterior mesoderm” L192.

3) I would also change the TBXT/SOX2 nomenclature in Figures to match the text.

Thanks for noting that the nomenclature was inconsistent in Fig 3 and Fig 4, this has now been corrected

---

Third decision letter

MS ID#: DEVELOP/2024/202983

MS TITLE: In vitro modelling of anterior primitive streak patterning with human pluripotent stem cells identifies the path to notochord progenitors

AUTHORS: Miguel Robles-Garcia, Chloe Thimonier, Konstantina Angoura, Ewa Ozga, Heather MacPherson, and Guillaume Blin
ARTICLE TYPE: Research Article

I am happy to tell you that your manuscript has been accepted for publication in Development, pending our standard publication integrity checks.

Reviewer 1

Advance summary and potential significance to field

See my comments in the previous rounds of revision. The manuscript was alreday good for publication in the previous round of revision in my opinion. Nothing else to add here.

Comments for the author

See my comments in the previous rounds of revision. The manuscript was alreday good for publication in the previous round of revision in my opinion. Nothing else to add here.

Reviewer 2

Advance summary and potential significance to field

First I would like to thank the authors for the additional quantifications conducted. These clarify the conclusions of the manuscript. I still don't think citing Forlani et al. (2003) or simply labeling APS/PPS resolves the issues with Figure 1A but this shouldn't be the reason for not to publish these important micropattern treatments. I am in favor of the publication.

Comments for the author

I do have concerns about the Fig8:

1) 8B how does shades of purple compare, NMC vs NMP?

2) 8C shows Nodal isn't needed for notochord after APS. Unclear what evidence suggests this. Do the authors have any more specific markers for the intermediate APS population (besides lowered SOX2) GSC, LHX1?

Fig2A does not enough quality on the cluster behaviour plots above heatmaps.

© 2024. Published by The Company of Biologists under the terms of the Creative Commons Attribution License (https://creativecommons.org/licenses/by/4.0/).
